# Acceptability of self-sampling and self-testing for infections: a rapid systematic review on public users’ views

**DOI:** 10.1186/s12889-025-21773-w

**Published:** 2025-02-20

**Authors:** Aleksandra J. Borek, Caity Roleston, Runa Lazzarino, Mineli Cooray, Gail Hayward, Nia Roberts, Edward Blandford, Tom Fowler, Sarah Tonkin-Crine

**Affiliations:** 1 Nuffield Department of Primary Care Health Sciences, University of Oxford, Oxford, UK; 2 National Institute for Health and Care Research (NIHR) Health Protection Research Unit in Healthcare Associated Infections and Antimicrobial Resistance, University of Oxford, Oxford, UK; 3 Bodleian Health Care Libraries, University of Oxford, Oxford, UK; 4 UK Health Security Agency, London, UK; 5 William Harvey Research Institute, Queen Mary University of London, London, UK

**Keywords:** Self-testing, Self-sampling, Testing at home, Diagnostics, Infections, Infectious diseases, Pandemic preparedness, Public health, Systematic review, Qualitative synthesis

## Abstract

**Background:**

Self-sampling and self-testing have been increasingly used for sexually transmitted infections (STIs) and quickly became widespread during the COVID-19 pandemic. User acceptability, preferences, and experiences are important factors affecting self-sampling/self-testing uptake. Understanding these factors is key to managing infections and planning responses to health emergencies. This review aimed to identify user views and experiences related to the acceptability, usability, motivations and preferences for self-sampling/self-testing for infections.

**Methods:**

We conducted a rapid systematic review. We searched Medline, EMBASE, PsycINFO, CINAHL, and Web of Science, limiting records to those published in English between 2014 and 2023. We also searched manually for additional peer-reviewed and grey literature. We included reports of public users’ views on self-sampling/self-testing for any symptomatic and asymptomatic infections (except human papillomavirus) with qualitative, mixed-methods or survey data relevant to the review aim. Data were extracted into tables and qualitative findings were coded in NVivo. We synthesised data narratively.

**Results:**

We identified 194 eligible reports, including 64 from Europe (which we prioritised for detailed synthesis) and 130 from outside of Europe. In Europe, the studied infections were respiratory (*n* = 42, including 37 for COVID-19), STIs/HIV/genital infections (*n* = 20), and hepatitis C (*n* = 2). Findings indicate that users found self-sampling/self-testing acceptable across infection/sampling types, populations, settings, and countries. Users wanted self-sampling/self-testing to help determine infection status and protect others. The main benefits were privacy and convenience, helping reduce the potential stigma of STIs/HIV/genital infections, and (for COVID-19) informing behaviour (e.g., socialising, self-isolating) and contributing to research. Easier to perform and less invasive sampling approaches were more acceptable. However, some participants reported challenges to self-sampling/self-testing, such as not understanding instructions, pain/discomfort in collecting samples, and lack of confidence in interpreting results.

**Conclusions:**

This review synthesised evidence on the acceptability of SS/ST and factors affecting it across different infections, sampling approaches, settings, and populations. Evidence shows that most people with experience of self-sampling/self-testing found it acceptable and were willing to accept some discomfort in favour of several perceived benefits. This amenability to self-sampling/self-testing could be leveraged for diagnosing infections and preventing transmission. It can be used to support the viability of new models of clinical care and pandemic preparedness.

**Trial registration:**

The review was pre-registered on PROSPERO (ref. CRD42024507656).

**Supplementary Information:**

The online version contains supplementary material available at 10.1186/s12889-025-21773-w.

## Background

Despite the 20^th^-century optimism that antimicrobial drugs and vaccines could help eradicate infectious diseases, communicable diseases have remained a top public health priority [1, 2]. In 2019, according to the World Health Organisation (WHO), communicable diseases were among the 10 leading causes of death globally [3]. Since the emergence of the Severe Acute Respiratory Syndrome Coronavirus 2 (SARS-CoV-2), the WHO has attributed over 7.1 million deaths to COVID-19, which was the leading cause of death during the pandemic [4]. As treating infectious diseases with antimicrobials inevitably accelerates antimicrobial resistance, infection prevention and control strategies (including diagnostics) are critical for public health. We need effective, timely and acceptable diagnostics to identify those infected to prevent transmission and enable prompt diagnosis and targeted treatment. Self-testing can help in the fight against infections and the ‘silent pandemic’ of antimicrobial resistance.

Self-sampling (SS) and self-testing (ST) are a promising public health strategy to help manage infections. As SS/ST can be performed in the community (e.g., at home) by lay members of the public, they overcome some of the barriers to traditional laboratory or point-of-care testing by healthcare professionals (HCPs), such as the need to access healthcare services. ST has been common for sexually transmitted infections (STIs), in particular human immunodeficiency virus (HIV), to promote more regular testing among high-risk populations. Since 2016, the WHO has recommended ST as a safe, accurate, reliable, convenient and confidential option for HIV testing [5]. During the COVID-19 pandemic SS/ST for SARS-CoV-2 was widely used by the public, with many countries implementing it as a strategy to limit infection spread. This was despite the unavailability and issues with supply chains at the start of COVID-19 and other pandemics/epidemics. Moreover, the normalisation of remote and digitally-enabled primary care [6], ‘virtual wards’ (‘hospitals at home’) [7, 8], and home monitoring equipment (e.g., for managing chronic conditions) [9] have the potential to increase the role of SS/ST. Advances in health technology devices, molecular diagnostic technologies, and rapid testing for early detection of pathogens are also contributing to the popularisation of SS/ST [10, 11]. The types of tests have evolved and their usability has improved over time, making them more likely to be adopted.

Many factors affect the uptake and sustained use of health technologies [12]. The characteristics of the technology itself are important, such as material and knowledge-related features (e.g., accuracy, knowledge needed to use the technology). The diagnostic accuracy of self-collected, compared to professionally collected, samples for influenza [13], upper airway infections [14], chlamydia and gonorrhoea [15], and HIV [16] has been found to be acceptable in meta-analyses. Despite pooled sensitivities exceeding 73% [17–20], more variability was found in SARS-CoV-2 SS/ST, depending on different factors, such as symptom presence [21, 22], when SS/ST was performed [18], and a sampling approach [20].

In addition to performance, whether and how health technology is adopted is influenced by its value proposition, characteristics of the adopters, and organisational and system factors [12]. User views and experiences of the technology, including acceptability, usability and preferences, are critical factors that can impede or drive the adoption of SS/ST. Existing reviews on the acceptability of SS/ST have focused on HIV testing [23, 24], often in low- and middle-income countries [25–27] and high-risk groups [28–30]. Others have focused on human papillomavirus (HPV) SS/ST as part of cancer screening [31–33], including in Muslim women [34], women living with HIV [35], and females in Africa and Latin America [36, 37]. One review explored experiences of internet-based ST for STIs [38]. To our knowledge, no review has synthesised users’ views on SS/ST *across* different infections, sampling approaches and populations. Addressing this gap can inform public health strategies for diagnosing and managing infections and preventing transmission in routine care and health emergencies.

This review aimed to synthesise public users’ experiences and views about the acceptability of SS/ST and identify the perceived barriers to, and enablers of, SS/ST for infections. The key concepts used in this review, clarifying its scope, are defined in Table 1.

**Table 1:** Definitions of the key concepts used in the review

• **Self-sampling (SS)** – individual users collecting their own bodily sample(s) (e.g., blood, saliva, urine) which are then professionally tested; • **Self-testing (ST)** – individuals collecting their own bodily sample(s) and performing the test procedure and interpreting the result; • **Infection** – presence of infection-causing pathogens (e.g., bacteria, viruses, fungi or parasites) in/on individuals’ bodies that can be non-symptomatic or symptomatic and causing an infectious disease (that is a disorder caused by the pathogens); • **Users** – here we define them as members of the general public performing SS/ST; • **Acceptability** – users’ views related to the acceptability of SS/ST, which we broadly defined as including also experiences and satisfaction with performing SS/ST, views on usability of SS/ST devices, willingness to self-sample/self-test, preferences and motivations for SS/ST.	

## Methods

We used rapid systematic review methods and Cochrane recommendations [39–42]. Rapid, or ‘restricted’, reviews employ abbreviated systematic review methods to accelerate knowledge production, with sensitivity to context and practical application [42, 43]. The review was pre-registered on PROSPERO (CRD42024507656) and reported following the PRISMA guideline [44].

### Search strategy

We designed and piloted an electronic search strategy using relevant terms (including MESH terms), mapped onto the PICOS framework [45] (Table 2). We searched Medline, EMBASE and PsycINFO via OvidSP, CINAHL via EBSCOHost and Science Citation Index & Social Science Citation Index via Web of Science, limiting the results to English language and 1 January 2014 to 15 December 2023.

**Table 2:** PICOS-guided search terms and selection criteria

PICOS	Search terms	Inclusion criteria	Exclusion criteria
**Population**	No search terms to keep the search broad	Public users of SS/ST, i.e. individual members of the public, patients, parents/carers (when testing children) with experience of SS/ST	- Professionals - Public without experience of SS/ST
		Note: when studies included mixed samples (e.g., public and professionals, with and without experience of SS/ST), we included studies only if the views of eligible participants were reported separately from ineligible participants or it was indicated which sub-groups the findings/data were relevant to	
**Intervention**	Set of search terms related to SS/ST	- Self-sampling or self-testing - Parents/guardians sampling/testing children	Sampling/testing done by professionals or carers/staff for residents in care homes
**Condition**	No search terms in order to keep the search broad	- Symptomatic and non-symptomatic infections, such as acute respiratory, urinary, genital, skin or sexually transmitted infections - Presence of infectious pathogens or markers of infections	- SS/ST that is not specific to infections (e.g., for unspecified conditions, microbiome testing) - SS/ST for antibodies (if aimed at identifying an immune response (e.g. to a vaccine), not a current infection - SS/ST for HPV (as this is aimed at cancer prevention)
**Outcome**	Search terms related to views, experiences, acceptability and usability	Self-reported users’ views and experiences related to acceptability of SS/ST, preferences and motivations for SS/ST, satisfaction with SS/ST, perceived barriers and facilitators to SS/ST (incl. related to usability, users’ views on performance/accuracy)	Hypothetical views of those without experience of SS/ST
**Study type**	Qualitative, mixed methods and survey studies	Peer-reviewed and non-peer-reviewed qualitative, mixed-methods or survey studies	Articles without empirical data
**Other**	Search limited to citations from 2014	N/A	- Not in English language - Without full text - Published before 2014

We conducted backward and forward citation searches of selected reviews and reference lists of included papers, and searched key terms in Google Scholar. Preprints were included in the main database search. We asked experts through our UK and European professional networks about relevant papers or reports. Finally, we reviewed the UK Government and UK Health Security Agency (UKHSA) online reports on COVID-19/SARS-CoV-2 testing. Supplementary Document 1 reports our search strategy, with further details.

### Study selection

Reports were included if they described public users’ self-reported views and experiences related to the acceptability of SS/ST for (non)symptomatic infections or the presence of infectious pathogens (Table 2). Acceptability covered also motivations for, and satisfaction with, SS/ST and perceived barriers and enablers of SS/ST. We included peer-reviewed and non-peer-reviewed qualitative, mixed methods or survey studies that reported empirical data/findings.

We developed and piloted screening instructions. Two researchers used them to screen titles/abstracts and full texts and used a standardised Excel form to record decisions and comments. As a team, we discussed and resolved discrepancies and uncertainties about eligibility. Twenty-five percent of excluded titles/abstracts and then full texts were double-screened by a second reviewer. Where details to establish eligibility were unclear, we checked them with the study authors.

After screening, due to time restrictions, we prioritised studies conducted in Europe for quality appraisal and detailed data extraction/synthesis because they were considered the most relevant to inform domestic UK policy and practice.

### Data extraction and synthesis

For the prioritised studies conducted in Europe, we used standardised pre-formatted tables to extract study characteristics, infection and SS/ST types, and details of SS/ST procedures (e.g., setting, frequency, instructions). From studies reporting relevant survey results, we extracted relevant measures/items and results. We summarised the results of similar questionnaire items (e.g., acceptability, ease of understanding of SS/ST instructions, ease of performing SS/ST), taking into account different sampling and infection types. From studies that reported relevant qualitative findings, we summarised the main findings related to acceptability, barriers/disadvantages, and enablers/benefits of SS/ST. We further condensed the findings on similar terms/concepts. We then coded qualitative findings in NVivo, using a deductive framework based on the topics identified during the earlier steps. Additionally, we reviewed all studies to extract data specifically on comparisons between groups of participants (e.g., with different demographics, sampling approaches, types of tests etc.) and on those who rejected SS/ST. We synthesised the evidence narratively according to the main topics identified in the data extraction and analysis. The accuracy of the extracted data was double-checked during evidence synthesis, with frequent checks of original reports but without confirming data with study authors.

### Quality assessment

We used the Mixed Methods Appraisal Tool (MMAT) [46] to appraise the quality of peer-reviewed reports from Europe.^1^ A second reviewer independently assessed 25% of studies. We did not exclude studies based on quality. We did not assess the non-peer-reviewed reports because, as grey literature, they lacked enough details to permit quality appraisal.

### Additional post-hoc analysis

Additionally, we examined eligible but non-prioritised studies conducted outside of Europe to explore how our main findings compare with them. We extracted the basic characteristics of these studies into a table, including infection and sampling types, settings of SS/ST, participant type and number, and briefly summarising the key findings related to acceptability. We used another table to list barriers and enablers of SS/ST in non-European studies, comparing them with those reported in European studies.^2^

## Results

### Overview

**Fig. 1:**
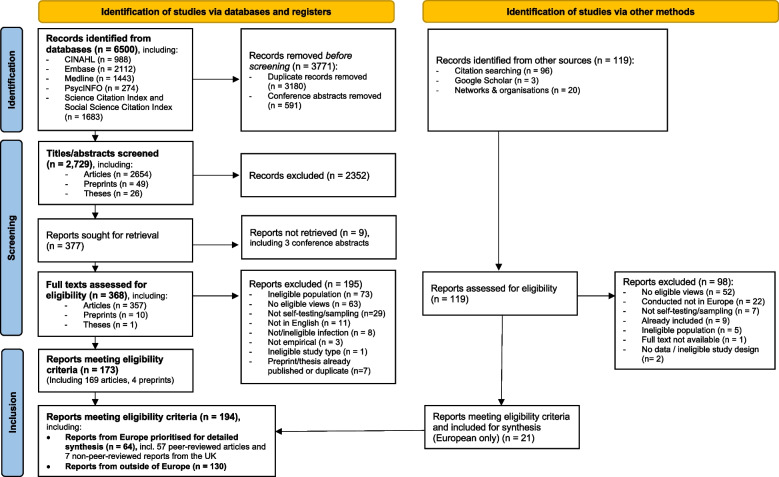
PRISMA study selection flowchart

We identified 6,500 records and, after removing duplicates and conference abstracts, screened 2,729 titles and abstracts, and then 368 full texts. From database searches, 173 reports met the eligibility criteria. From other sources, we identified 21 eligible European reports. This resulted in 194 eligible reports, including 64 from Europe and 130 from outside of Europe (Fig. 1, Table 3).

**Table 3:** Overview of all eligible studies

**Region**	**Study design** (No. reports)	**Infection types**	**No. reports**	**Additional details**
**TOTAL number of eligible reports**			**197**	**176 reports from database searches, 21 from other sources**
Europe *(Studies* *prioritised* *for detailed* *synthesis)*	Qualitative (10)	RTIs	5	All COVID-19
		STIs	5	3 on HIV, 2 on chlamydia
	Mixed-methods (17)	RTIs	10	8 on COVID-19, 1 on respiratory microorganisms, 1 on sore throat pathogens
		STIs	6	5 on HIV, 1 on chlamydia
		Hepatitis C	1	
	Surveys (37)	RTIs	27	24 on COVID-19, 3 on respiratory microorganisms
		STIs/ genital	9	7 on multiple STIs/genital infections, 2 on HIV only
		Hepatitis C	1	
	**Europe – no. reports**		**64**	**57 peer-reviewed articles and 7 non-peer-reviewed UK reports**
Africa	Qualitative (19)	STIs	19	17 on HIV, 2 on STIs
	Mixed-methods (16)	RTIs	1	COVID-19
		STIs / genital	14	11 on HIV, 2 on STIs, 1 on bacterial vaginosis
		Hepatitis C	1	
	Surveys (24)	STIs / genital	21	17 on HIV, 2 on genital schistosomiasis, 1 on HIV and hepatitis B and C, 1 on STIs, 1 on bacterial vaginosis
		Hepatitis C	2	
		Malaria	1	
	**Africa – no. reports**		**59**	**All peer reviewed articles**
North America	Qualitative (4)	RTIs	1	COVID-19
		STIs	3	2 on HIV, 1 on HIV and syphilis
	Mixed-methods (11)	RTIs	4	3 on COVID-19, 1 on influenza
		STIs	7	3 on STIs, 4 on HIV
	Surveys (31)	RTIs	11	10 on COVID-19, 1 on respiratory pathogens
		STIs	18	7 on HIV, 9 on multiple STIs, 1 on trichomonas vaginalis, 1 on STIs & COVID-19
		UTIs	2	
	**North America – no. reports**		**46**	**All peer reviewed articles**
Asia	Qualitative (3)	RTIs	1	COVID-19
		STIs	2	HIV (one reported quantitative results despite reporting qualitative data collection)
	Mixed-methods (4)	STIs	4	HIV
	Surveys (11)	STIs/ genital	9	7 on HIV, 1 on HIV and syphilis, 1 on STIs
		Hepatitis C	2	
	**Asia – no. reports**		**18**	**All peer reviewed articles**
South America	Surveys (5)	RTIs	1	COVID-19
		STIs	4	3 on HIV, 1 on STIs
	**South America – no. reports**		**5**	**All peer reviewed articles**
Australia	Qualitative (1)	STIs	1	HIV
	Surveys (1)	STIs	1	Chlamydia
	**Australia – no. reports**		**2**	**Both peer reviewed articles**

*Abbreviations*: *HIV* human immunodeficiency virus, *RTIs* respiratory tract infections, *STIs* sexually transmitted infections, *UTIs* urinary tract infections

Of the 64 prioritised European studies, 24 were peer-reviewed articles of studies conducted in the UK [3–26], and in 10 other European countries (*n* = 33) [27–59]. Seven were non-peer-reviewed reports of studies or sets of studies conducted in the UK, including a multi-chapter study report [60] and six UKHSA reports [61–66]. Most studies used surveys (*n* = 37), followed by mixed (*n* = 17) and qualitative (*n* = 10) methods. Fifty-three reports comprised relevant quantitative results (from quantitative and mixed-methods studies) and 27 relevant qualitative findings (from qualitative and mixed-methods studies).^3^ The studied infections were: respiratory tract infections (RTIs) (*n* = 42, including 37 studies on COVID-19), STIs/HIV/genital infections^4^ (*n* = 20, including ten on HIV only, three on a range of STIs with/without HIV, three on chlamydia and gonorrhoea, two on chlamydia only, and two on a range of genital infections and microbes [e.g., chlamydia, bacterial vaginosis, yeast infection]), and hepatitis C (*n* = 2). Table 4 summarises the study characteristics; Supplementary Table S1 – details of SS/ST approaches; Supplementary Tables S2-S4 – findings of quantitative, qualitative and non-peer-reviewed reports.

**Table 4:** Characteristics of the synthesised 64 reports from Europe

**First Author, year (reference), country, study name**	**Infection type**	**Study design** (data synthesised)^*^	**Participants**	**Data collection methods & time**
Agusti 2022 [62], Spain, TESTA’T COVID Project	RTI (SARS-CoV-2)	Survey	*N* = 196 education professionals recruited from 23 schools	Online questionnaire, 15 December 2021 to 15 February 2022
Ahmed-Little 2016 [49], UK: England	HIV	MM (survey, free text comments)	*N* = 2447 (of 5179 who requested ST kits) completed questionnaires; 756/2447 provided free-text comments; aged 16 + and identified as high risk of HIV	Paper questionnaire, with free-text comments, June 2011—December 2012
Aiano 2021 [78], UK: England	RTI (SARS-CoV-2)	Survey (data on children’s SS)	*N* = 9,592 participants from 86 primary schools involved in testing, incl. *n* = 116 children who completed surveys on SS	Questionnaires, June – July 2020
Atchison 2021 [68], UK: England, REACT study	RTI (SARS-CoV-2)	MM	*N* = 25 interviews (reported in the supplementary material); *N* = 8754 adult users completed surveys for 1-type kits and *N* = 2957 for 2-type kits; a random sample of 30,000 households invited	Interviews, online survey, May 2020
Baetselier 2019 [69], Belgium, Colli-Pee study	STIs (range)	Survey	*N* = 164 respondents, Men who have Sex with Men (MSM) at high risk of HIV recruited	Questionnaire, time n/r
Bauld 2023 [64], UK: Scotland	RTI (SARS-CoV-2)	MM	*N* = 300 pilot survey; *N* = 1750 main survey; *N* = 48 interviews; staff and students at the University of Edinburgh	Questionnaire & semi-structured interviews, 04/2021–02/2022
Blake 2020 [66], UK: England	RTI (SARS-CoV-2)	MM	*N* = 285, incl. 215 undergraduate students and 70 staff from the University of Nottingham	Online questionnaire; interviews and focus groups, Sept. – Oct. 2020
Brown 2019 [50], UK: Scotland	STI (Chlamydia)	MM (interviews)	*N* = 20 women aged 16–25 attending sexual health clinic in Edinburgh	Interviews, December 2018—February 2019
Camus 2021 [94], France	STIs (range) and other genital infections (bacterial vaginosis, yeast infection, group B streptococcus)	Survey	*N* = 1027 respondents (out of 1028 participants), women recruited from 11 clinical centres, aged 18–65 if they presented vaginal/cervical sampling indications for screening for specified infections (bacterial vaginosis, yeast infection, STI, group B streptococcus)	Questionnaire, participants were recruited October 2015—March 2018
Castell 2014 [57], Germany	STIs (range) and other genital infections (bacterial vaginosis, human papillomavirus)	Survey	*N* = 162 pregnant women and within 30 days of childbirth recruited from a larger study	Questionnaire, September—December 2012
Colom-Cadena 2022 [59], Spain	RTI (SARS-CoV-2)	Survey	*N* = 346, incl. *n* = 305 students aged 9 + from 2 schools and 4 summer camps, *n* = 41 staff from the summer camps	Questionnaires, April to August 2021
Davies 2021 [92], UK: England, REACT-2 study	RTI (SARS-CoV-2)	Survey	*N* = 5328 non-healthcare key workers (the police or fire service)	Questionnaire, participants recruited 1 June—10 July 2020
Denford 2021 [79], UK: England	RTI (SARS-CoV-2)	Qualitative	*N* = 35 participants, incl. 17 who declined ST, recruited from the larger study and were purposively sampled	Interviews, December 2020 to January 2021
Fajardo 2022 [70], Georgia	Hepatitis C	MM (interviews)	*N* = 40, 18 + years, did not know their hepatitis C serostatus or tested negative > 1 year ago, reported use of un-prescribed intravenous drugs or had 1 + anal sex episode with another man within the past year	Qualitative cognitive and in-depth interviews, December 2019—June 2020
Flipse 2022 [96], Netherlands	RTI (respiratory microorganisms)	Survey	*N* = 63, technicians of the Laboratory of Clinical Microbiology and Infectious Diseases and their family members showing RTI symptoms	Questionnaire, April 2019 to March 2020
Grandahl 2020 [58], Sweden	STIs (chlamydia, gonorrhoea)	Survey	*N* = 1,785 users of the public healthcare website	Questionnaire, December 2018—July 2019
Grandahl 2020 [86], Sweden	STIs (chlamydia, gonorrhoea)	Qualitative	*N* = 20, 15 + years who had ordered a self-test from the national eHealth website, sampled for a range of ages and sociodemographic areas	Telephone interviews, 2019
German 2023 [95] & Nikolaou 2023 [65], UK: England, FAMILY Micro	RTIs (respiratory micro-organisms)	MM	40 healthy families with 2 adults (aged 18–60) and 1–3 children (28 days—17 years); 12 interviews (convenience sampling) with participants from different households; questionnaires completed by members of the 33 families completing the study	Questionnaire, online interviews, March and September 2021
Gillam 2021 [60], UK: England	RTI (SARS-CoV-2)	MM (Survey)	*N* = 458 adults who lived/worked on the Norwich Research Park	Questionnaire, dates n/r
Haussig 2019 [61], Germany	RTIs (range)	Survey	*N* = 103: 73 adults & 30 children recruited through GrippeWeb online platform, and 11 employees of the Robert Koch Institute were invited	Questionnaires, January to July 2016
Hirst 2021 [67] & Wanat 2021 [89], UK: England, FACTS study	RTI (SARS-CoV-2)	MM (Survey)	University of Oxford students and staff, *N* = 213 survey sample, *N* = 18 interview sample	Questionnaire, 1 December 2020—7 January 2021
		MM (interviews)		Interviews, 11 December 2020—18 January 2021
Hoehl 2021 [108], Germany (research letter)	RTI (SARS-CoV-2)	Survey	*N* = 635 (of 711 participants) submitted records, school teachers in two rural districts and one city in Hessen	Survey, time n/r
Iruzubieta 2021 [93], Spain	RTI (SARS-CoV-2)	Survey	*N* = 1,022 selected using stratified sampling according to geographic area, age, and gender from the ETHON cohort from Cantabria, Spain	Online survey, 27 April to 29 May 2020
Jing 2021 [99], UK: Northern Ireland	RTI (SARS-CoV-2)	Survey	*N* = 1539 answered usability questions, recruited through university and media, randomly selected representative sample	Questionnaire, September 2020
Jing 2022 [106], UK: Northern Ireland	RTI (SARS-CoV-2)	Survey	*N* = 264, recruited via emails to the Pandemic Database, sampled for a range of age, gender and education for a representative sample	Questionnaire, October to December 2020
Jones 2021 [85], UK: England	RTI (SARS-CoV-2)	Qualitative	*N* = 30 students at Durham University	Semi-structured online interviews, 5—12 March 2021
Lafort 2023 [80], Belgium	RTI (SARS-CoV-2)	Survey	*N* = 27,397 survey 1 and *N* = 22,354 survey 2, adult Belgian residents (general population)	Two routine national surveys, December 2021
Leenen 2020 [77], Netherlands	STIs (range)	Survey	MSM attending HIV treatment clinic in Maastricht	Online questionnaire, March to May 2018
Lindner 2021 [100], Germany	RTI (SARS-CoV-2)	Survey	*N* = 146 adults with symptoms of COVID-19 from the ambulatory COVID-19 testing facility in Berlin	Questionnaire, 30 November to 11 December 2020
Loos 2016 [53], Belgium, Swab2know study	HIV	MM (qualitative)	*N* = 41 Sub-Saharan African migrants in Antwerp from selected community settings / organisations	Observations, unstructured interviews, Dec. 2012—June 2013
Lown 2023 [51], UK: England, Scores and Swabs to Self-Assess Sore Throat	RTI (Sore throat)	MM	*N* = 42 (32 adults, 10 parents/carers of children) survey, N = 38 interviews; recruited through general practices and social media	Questionnaire, structured interviews, time n/r
Marinos 2022 [81], Greece	RTI (SARS-CoV-2)	Survey	*N* = 1000 convenient sample of students (13–18 years old) selected from schools in Athens, Greece	Questionnaire, 20 September—20 October 2021
Martin 2021 [48], UK: England	RTI (SARS-CoV-2)	Survey	*N* = 524 adult contacts of confirmed SARS-CoV-2	Questionnaire, 11 and 23 December 2020 and 4 to 12 January 2021
Møller 2022 [98], Denmark	RTI (SARS-CoV-2)	Survey	*N* = 890,827 participants included in the analysis, 18 + years, had made an appointment for PCR testing at the COVID-19 test centre in Aarhus	Questionnaire, 21 January 2021 to 4 April 2021
Nash 2021 [55], Italy	HIV	Survey	*N* = 28 participants recruited through clients of two Italian NGOs, 18 + years and never having used an HIV ST before	Questionnaire, time n/r
Powell 2016 [54], UK: England	STI (Chlamydia)	Qualitative	*N* = 19 university students of whom 11 had self-tested. Recruited from those who used a ST for chlamydia or whose partner used one	Semi-structured interviews, time n/r
Prazuck 2016 [102], France	HIV	Survey	*N* = 411, incl. 264 (208 used the test) in 1st and 147 in 2nd sub-study; recruited during screening programs by AIDS prevention associations and a testing centre at Bichat Hospital, Paris	Self-administered questionnaires, April to July 2014
Prazuck 2021 [47], France	RTI (SARS-CoV-2)	Survey	*N* = 141, recruited from two nuclear plants, hospital vaccination clinic and non-medical hospital staff in Central France	Questionnaire, 20 March to 5 May 2020
Prazuck 2022 [101], France	RTI (SARS-Cov-2)	Survey	*N* = 108, incl. 68 adults, parents/carers of 24 children (3–11 years), and 16 teenagers (12–15 years), recruited from COVID-19 hospital testing unit, medical analysis laboratory and hospital’s infectious diseases department	Questionnaire, data collection time n/r, study approved in October 2020
Prinsenberg 2022 [84], Netherlands, NoMoreC testing	Hepatitis C	Survey	*N* = 54 (from 152 who ordered tests), MSM recruited through a C-test service website, offline by the NoMoreC volunteers, and flyers at a specialist pharmacy in Amsterdam	Online questionnaire, 22 February 2018—31 December 2020
Rohr 2022 [82], Germany	RTI (SARS-CoV-2)	Qualitative	*N* = 67, incl. 37 ST users, aged 7 + selected via civil registration services, sampled purposively (age, sex, educational background, study arm, test/ questionnaire uptake or rejection)	Semi-structured in-depth interviews (online or telephone), December 2020 to February 2021
Schuit 2022 [107], Netherlands	RTI (SARS-CoV-2)	Survey	*N* = 5,766 (out of 6,497 with COVID-19 symptoms), aged 16 + presenting consecutively at 3 public health service COVID-19 test sites	Online questionnaire, 21/12/2021 to 10/02/2022
Schuit 2022 [97], Netherlands	RTI (SARS-CoV-2)	Survey	*N* = 2,950, aged 16 + recruited consecutively at three public health service COVID-19 test sites	Online questionnaire, 9—26 September 2021
Seguin 2018 [73], UK: England, HAUS study (non-peer-reviewed)	HIV	MM (survey—chapter 7, interviews—chapter 9)	*N* = 62 completed questionnaire (out of 66 who returned samples), *N* = 21 interviewed, Black African recruited via general practices and community organisations in London	Brief acceptability questionnaire, telephone interviews, April and July 2016
Sweeney-Reed 2021 [83], Germany	RTI (SARS-CoV-2)	Survey (questionnaires 3 and 4 only)	*N* = 235 completed questionnaire 4 on test method, *N* = 290 completed acceptance questionnaire 3; pupils attending a primary school (aged 6–10 years) and a secondary school (10–18 years) in Magdeburg	Questionnaires, December 2020 to June 2020
Tonen-Wolyec 2020 [103], France	RTI (SARS-CoV-2)	Survey	*N* = 167 enrolled; *n* = 83 in a usability sub-study 2, *n* = 84 in test results interpretation sub-study 3, *n* = 83 in satisfaction after ST, *n* = 84 after test interpretation in sub-study 4; aged 18 + , recruited door-to-door in 15 neighbourhoods in Strasbourg and its suburbs, France	Paper-based, face-to-face, semi-structured and self-administered questionnaires, April to May 2020
Tonen-Wolyec 2021 [63], France	RTI (SARS-CoV-2)	Survey	*N* = 106, adults using laboratory testing service for COVID-19	Paper-based questionnaires, April – May 2021
UKHSA 2022 [104], UK (non-peer-reviewed report)	RTI (SARS-CoV-2)	MM (Quant. scores & qual. comments)	*N* = 8 Blind and Partially Sighted (BPS) people recruited through the BPS Stakeholder Forum, selected with diverse demographic characteristics and conditions	User feedback, incl. quantitative scores of difficulty and free comments, time n/r
UKHSA 2022 [105], UK (non-per-reviewed report)	RTI (SARS-CoV-2)	MM (Quant. scores & qual. comments)	*N* = 29 BPS people recruited by the Royal National Institute of Blind People (1^st^ sub-study, interviews), *N* = 29 (interviews) and *N* = 69 (survey) (2^nd^ sub-study)	Interviews, questionnaire, time n/r
UKHSA 2023 [74], UK (non-peer-reviewed report)	RTI (SARS-CoV-2)	MM (surveys, focus groups)	General public in the UK, data extracted was based on regular surveys of ~ 2000 people aged 16 + and ~ 1000 people; qual. data extracted was based on 4 focus groups (number of participants n/r)	Fortnightly surveys between February 2021 and February 2023, focus groups (time n/r)
UKHSA 2023 [75], UK (non-peer-reviewed report)	RTI (SARS-CoV-2)	MM (survey, interviews)	(1) teachers and pupils of Special Educational Needs and Disability (SEND) school (Pilot *n* = 2983; survey teachers *n* = 173; students *n* = 35; parents/guardians *n* = 7; Interviews parents *n* = 17; head teachers *n* = 4); (2) prison: staff and prisoners (survey *n* = 37); (3) residential adult social care home (not eligible)	(1) SEND: participant questionnaire (staff & students) & interviews with staff and parents; (2) prison: survey & interviews with staff; time n/r
UKHSA 2023 [109], UK (non-peer-reviewed report)	RTI (SARS-CoV-2)	Survey	Number of survey participants unclear	Questionnaires, 8/06—27/07/2020; 16/10 – 4/11/2020
UKHSA 2024 [76], UK (non-peer-reviewed report)	RTI (SARS-CoV-2)	Survey	(1) HEI: students living away from home intending to return home over winter break (survey *n* = 2214, 71% of them reported that they had tested during study period); (2) schools & colleges: survey of parents and students (*n* = 1,364 of those who had tested in the preceding 7 days and had no positive result and *n* = 145 who had not taken the test)	(1): HEI: survey; 27/11 – 20/12/2020; (2) schools & colleges: parents & students survey; 103 – 4/04/2021
van Loo 2017 [71], Netherlands	STIs and HIV	Survey	*N* = 183 MSM, visiting the STI clinic of the public health service South Limburg, and *N* = 34 HIV and HBV positive patients visiting the outpatient clinics in the Maastricht University Medical Centre	Questionnaire, January 2012 to April 2015
Wachinger 2021 [87], Germany	RTI (SARS-CoV-2)	Qualitative	*N* = 26, incl. 6 school stakeholders and staff, 10 pupils and 10 parents; school staff participating in voluntary screening and pupils and their parents from a primary school in a peri-urban area	Qualitative in-depth interviews, 22 March to 21 May 2021
Weidlich 2023 [72], Germany	STIs (Chlamydia and gonorrhoea)	Survey	*N* = 236 MSM reporting > 2 condomless anal intercourses with ≥ 2 male sex partners in the last 24 weeks recruited during regular sexual health check-up visits from medical centres in Berlin, Cologne, and Munich	Online questionnaire, April 2021 to July 2022
Witzel 2019 [56], UK: England and Wales, SELPHI study	HIV	MM	*N* = 375 participants completed 3-month survey on acceptability, *N* = 10 interviewees, sampled purposefully from the trial; Cisgender and transgender men and transgender women who have anal sex with men, recruited through networking applications and Facebook	Online questionnaire, semi-structured remote interviews, time n/r
Witzel 2020 [88], UK: England and Wales, SELPHI		Qualitative	*N* = 37 interviewees, sampled purposefully from the trial, recruitment as above	Online and face-to-face interviews, May 2017 (pilot), January—October 2018 (during main trial)
Witzel 2020 [90], UK: England and Wales, SELPHI				
Witzel 2021 [52], UK: England and Wales, SELPHI		MM	*N* = 118 trans participants recruited from the trial (recruitment as above), *n* = 39 completed acceptability survey, *n* = 20 interviewees, purposefully sampled from the trial	Online questionnaire; online, phone or in-person interviews, April and October 2019
Nicholls 2022 [91], UK: England and Wales, SELPHI		Qualitative	*N* = 29 interviewees, sampled from the trial (recruited as above)	Remote (online/ instant messenger) interviews, April and October 2020
Würstle 2021 [114], Germany	RTI (SARS-CoV-2)	Survey	*N* = 63 adults hospitalized (not necessarily for COVID-19) at the University Hospital of the Technical University of Munich, recruited from the hospital; positive SARS-CoV-2 PCR test in the last 48 h	Questionnaire, April to October 2020

^*^Data type is indicated if only some data reported in the paper was relevant to this review and extracted

Abbreviations used in the table: *HIV * human immunodeficiency virus, *MM * mixed-methods, *MSM * men who have sex with men, *n/r *not reported, *PCR* polymerase chain reaction, *RTI * respiratory tract infection, *SS * self-sampling, *ST *self-testing, *STI *sexually transmitted infection

### Study quality

Among peer-reviewed European studies, most surveys (except two [47, 48]) were assessed as employing a sampling strategy consonant with the research questions. Lower quality of surveys related to nonresponse bias (assessed as high/unclear in 23 studies) and representativeness of the sample to the target population (unclear/not representative in 19 studies). Most mixed-methods studies were assessed as providing an adequate rationale for using mixed methods (except [49–52]), integrating the different components of the study to answer the research questions (except [52]), and adhering to the quality criteria of each tradition of the methods involved (except [49, 51, 53]). All qualitative studies had good methodological quality. Only one study [54] was assessed as lacking coherence between qualitative data sources, collection, analysis, and interpretation. Supplementary Table S5 reports the studies’ quality appraisal.

### Acceptability

Across sampling, test, infection, population and study types, we found that users considered SS/ST acceptable, with the majority (71.4–100%) of survey respondents indicating acceptability and a good overall experience [47, 49, 52, 55–67]. Most (71.4–99%) indicated a willingness to perform SS/ST in the future [51, 55, 59, 61, 63, 66, 68–72]. The high levels of acceptability were also evident in non-peer-reviewed reports of SS/ST for HIV [73] and SARS-CoV-2/COVID-19 among the general public [74] and in educational, special educational needs and disabilities (SEND) and prison settings [75, 76].

Studies (across study and infection types) that reported the uptake of SS/ST found that only a minority of participants rejected SS/ST [49, 50, 53, 56, 58, 65, 66, 68, 77–84].

### Enablers of and barriers to self-sampling/self-testing

Table 5 summarises the identified enablers of and barriers to SS/ST. They are discussed in more detail below where we report the findings organised by the main topics identified during analysis.

**Table 5:** Enablers and barriers to self-sampling/self-testing reported in Europe

**Enablers & benefits of ST/SS**	**RTI**	**STI**	**HCV**
	**(Reference numbers to included studies)**		
Motivation to determine one’s infection status, gain reassurance/ peace of mind/ sense of safety, and to inform one’s behaviour	[48, 62, 64, 66, 67, 74, 76, 79, 80, 82, 83, 85, 87, 89]	[53, 54, 58, 86, 88, 90]	[84]
Motivation to prevent infection transmission and protect others	[48, 59, 64, 66, 67, 74, 76, 79, 82, 83, 85, 87, 89]	[56, 86]	
A part of good citizenship, compliance with government guidelines/requirements, wanting to contribute to efforts to control the virus, and promote normalisation of SS/ST	[59, 64, 66, 83, 85]	[56]	
Convenience, practicality, ease of access, flexibility (related to timing, lifestyle, and options for returning samples)	[48, 64–66, 82, 85, 89, 105]	[49, 53–56, 58, 73, 77, 86, 88, 90]	[70, 84]
Interest in new health technology, scientific curiosity	[66, 82]	[52, 56, 88, 90]	
Motivation to contribute to research and/or public health initiatives	[64, 66, 82, 83, 87, 89]	[56]	
Experience of and familiarity with SS/ST	[51, 61, 74, 85, 87, 89, 95]	[52, 88]	
Control, autonomy over one’s health and when/where to test	[59]	[52, 54, 88, 90, 91]	
Privacy, confidentiality, a way of avoiding health services and the need for a physical examination, and avoiding associated judgment and stigma	[98]	[49, 52, 54–56, 69, 73, 86, 88, 90, 91]	[70]
No or low cost	[59, 82, 85]	[53, 54, 86, 90]	[84]
Accessibility, proximity of testing sites, without having to pre-book appointments	[64, 66, 85]	[54, 73]	
Ease of sample self-collection, ease of use the SS/ST device	[47, 48, 51, 59–68, 75, 80, 82, 83, 85, 89, 93, 95, 98–101, 103, 104, 106, 108, 114]	[49, 52–58, 69, 71–73, 77, 86, 88, 91, 102]	[70, 84]
Trust in test accuracy/ result validity	[48, 59, 62, 64, 67, 74, 82, 83, 85, 89]	[55]	
Trust in/credibility of study institution or test provider	[82]	[73]	
Clear instructions, understanding instructions	[47, 51, 60, 61, 66, 68, 75, 82, 89, 93, 97, 99, 101, 103–107, 109]	[49, 52, 56, 69, 71, 73, 77, 86, 88, 102]	[84]
Low discomfort/ invasiveness, no pain	[51, 61, 64, 65, 87, 95, 107]	[49, 53, 71, 73, 86]	
Rapid results	[59, 60, 62, 64, 82, 83, 85, 98]	[49, 53, 56, 71, 73, 86, 90]	
Easy access to results (digitally, sent home)	[82]	[73, 86]	
Ease of reading and understanding ST result, confidence in interpreting the result	[47, 48, 59, 62, 63, 67, 68, 76, 92, 97, 99, 101, 103, 106, 107]	[52, 56, 102]	
**Barriers & challenges to SS/ST**	**RTI**	**STI**	**HCV**
	**(Reference numbers to included studies)**		
Stigma associated with the infection			[84]
Low or no perceived risk of infection	[80]	[53, 73, 77, 86]	
Worry about potential positive result or not wanting to find out the result of SS/ST	[68]	[49, 73]	
Lack of time for SS/ST		[48, 51, 58, 59, 65, 67, 76, 82, 85, 89, 104]	[84]
Cost (prohibitive)	[80]	[54, 55]	[70]
Complaints about testing sites	Lack of privacy of booths & inconvenient opening times [64]; no walk-in options [85]		
Concerns about safety/ risks of infection transmission at testing sites	[64, 85, 89]		
Concerns/questions & wanting information about test accuracy/reliability	[48, 51, 59, 67, 76, 82, 85, 89, 104]	[54, 72, 86, 88, 91]	[70]
Difficulties with instructions	[48, 51, 60, 82, 100, 107]	[55, 58, 69, 71, 72, 77, 86, 88, 102]	[70, 84]
Difficulties collecting (sufficient) samples, difficult or inconvenient procedures, - when collecting samples from children	[51, 64, 66, 68, 81, 82, 89, 92, 100, 103–105, 107], [65, 95]	[49, 50, 54, 56, 69, 72, 73, 88, 102, 114]	[70, 84]
Problems with/ using the equipment	By BPS people [104, 105]	[49, 52, 56, 69, 73, 88]	
Concern/ uncertainty if SS/ST performed (in)correctly	[51, 67, 79, 82, 89, 104, 105, 107, 114]	[49, 50, 52, 54, 86, 88, 91]	
Challenges, concerns and inconvenience related to returning samples and timing of collecting samples	[60, 66, 82], for BPS people [104, 105], inflexible/ inconvenient timing [75]	[71, 73, 86]	
Discomfort, pain when collecting samples/ unpleasant sampling	[48, 51, 61, 64, 66, 67, 82, 85, 87, 89, 98, 105, 114], in children and young people: [76, 109]	[49, 50, 57, 71, 72]	
Other negative emotions associated with performing SS/ST	Fear of needles [66], disgust [82, 95], fear [81], scared because packaging included biohazard symbol [82], child’s distress when being swabbed [109]	Fear of needles [88], and fear of needles reported by those who did not return samples [73], embarrassment [50]	
Anxiety when performing ST without supervision/ advice, disconnected from care pathway	[59, 105]	[50, 54–56, 86, 88, 91]	
Anxiety waiting for results, perceived long time for results	[60, 64, 82]	[56, 58, 86, 88]	
Lack of clarify about how results would be communicated	[66, 75, 82]		[70]
Having to interpret ST results, difficulties interpreting the results	[59, 92, 104, 107]		[70]
Uncertainty about what to do when testing positive	[79, 80, 85]	[86]	

*Abbreviations*: *BPS * blind and partially sighted, *HCV *hepatitis C virus, *RTI * respiratory tract infections, *SS * self-sampling, *ST * self-testing, *STI * sexually transmitted infections

#### Motivations for self-sampling/self-testing

Across sampling/infection types, surveys found that the most frequently endorsed reason for SS/ST was to determine one’s infection status [48, 58, 64, 80, 84]. This was reflected in qualitative studies where SS/ST was seen as providing peace of mind, a sense of safety and reassurance about one’s health status [53, 54, 66, 79, 82, 85–87], and that of the sexual partner in the case of SS/ST for STIs/HIV [86, 88]. ST at home and implementing appropriate behaviours helped protect others and reduce transmission [56, 64, 66, 67, 79, 82, 85–87, 89]. Some perceived SS/ST as a way of acting as good citizens [56], setting an example for others [66], and promoting the acceptability and normalisation of SS/ST [52, 59]. Moreover, compared to other types of testing, SS/ST was seen as more convenient, practical, timely, easily accessible and fitting around people’s lifestyles better [49, 73, 90]. Some were also motivated by curiosity about new SS/ST technology [52, 56, 66, 82, 88, 90], and wanting to contribute to research (particularly during the COVID-19 pandemic) [64, 66, 67, 83, 87, 89]. There was some indication that experience and familiarity might increase the acceptability of SS/ST. For example, non-peer-reviewed surveys showed that ST with lateral flow devices (LFDs) for COVID-19 became more accepted over time [74], and a peer-reviewed study found that a prior experience with COVID-19 ST was perceived as subsequently making SS for sore throat more familiar and easier [51].

Similarly, for COVID-19, the main reasons for testing related to checking if people were infected (mostly, complying with government guidance and/or requirements) while giving people autonomy and control over when and where to test [59, 74, 76]. The acceptability of SS/ST was reinforced by the desire to protect others from SARS-CoV-2 [59, 66, 67, 79] avoid self-isolation (as a contact of a COVID-19 case) [79], and inform social and professional activities [74, 76, 85, 89].

For STIs/HIV/genital infections, the primary benefits of SS/ST were related to privacy and confidentiality. SS/ST was viewed as a valued alternative to being tested by healthcare professionals, avoiding the perceived stigma and judgment for HIV [49, 52, 88, 90, 91], STIs [54, 86], and hepatitis C [70]. Although stigma associated with the infection could also deter people from SS/ST, as reported for hepatitis C [84]. It allowed users to eliminate the need for physical examination by healthcare professionals [54], with anonymity making the testing process for HIV more comfortable and less intimidating [49]. For STIs/HIV, SS/ST was also perceived as increasing a sense of autonomy and feeling in control [52, 54, 88, 90, 91].

In contrast, the main self-reported reasons for not SS/ST (across infections) included: having already tested elsewhere [49, 56, 58, 73, 77, 84], obtaining the self-test for future use [56, 58, 80, 84], low or no perceived risk of infection (STIs [53, 58, 73, 77], COVID-19 [80]), worry about positive result or not wanting to know the result [49, 68, 73], and the lack of time to perform SS/ST [58, 65, 84].

Finally, across infections, studies reported that no or low cost was enabling SS/ST [53, 54, 59, 82, 84–86, 90]; whereas having to pay for or prohibitive cost were reported as barriers to SS/ST [54, 55, 70, 80].

#### Preferences for settings of self-sampling/self-testing

**Fig. 2:**
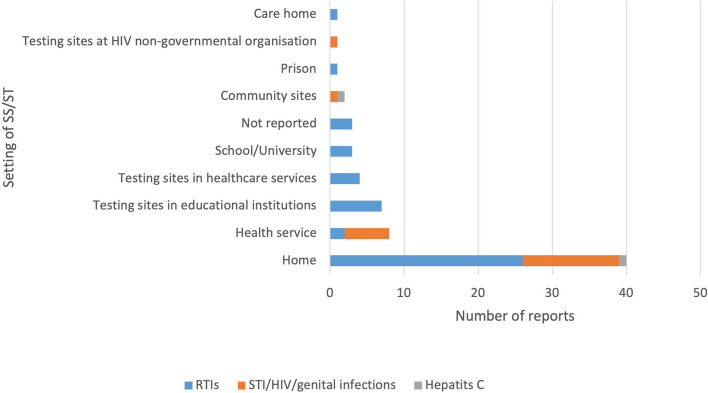
Types of settings of self-sampling/self-testing Note: There were four reports that reported SS/ST in two types of settings and one report with three types of settings; hence, the total number of reports in this figure is higher than the 64 included reports

Most SS/ST was performed at home (Fig. 2). The UKHSA reports focused on SS/ST for COVID-19 in specific settings: schools, colleges and universities [76], SEND schools and a prison [75].

Across sampling/infection types, surveys showed that respondents (60.9–93.2%) preferred SS/ST at home [57, 59, 62, 68, 70, 77, 92, 93]. However, two studies found no clear preference between SS for RTIs at home or school [83], or compared to sampling for genital infections by healthcare professionals in a healthcare setting [94]. In one survey study on HIV and other STIs in the Netherlands, only a minority of respondents preferred for SS at home [71].

In qualitative studies, participants described SS/ST at home as more flexible, time-saving, comfortable, and private than having to visit a clinic or be visited by a clinician to be tested [50, 52–54, 56, 70, 73, 82, 86, 88]. When SS/ST in testing sites, participants appreciated the accessibility and proximity of the sites, and no requirement for booking appointments [54, 64, 66, 73, 85]. However, some negative aspects of testing sites were also reported: the perceived lack of privacy of booths [64], inconvenient opening times [64] or lack of same-day appointments [85]. When SS/ST for SARS-CoV-2, some were concerned about potentially infecting others and wanted reassurance of measures minimising transmission risks [64, 85, 89].

#### Preferences for self-sampling approaches

**Fig. 3:**
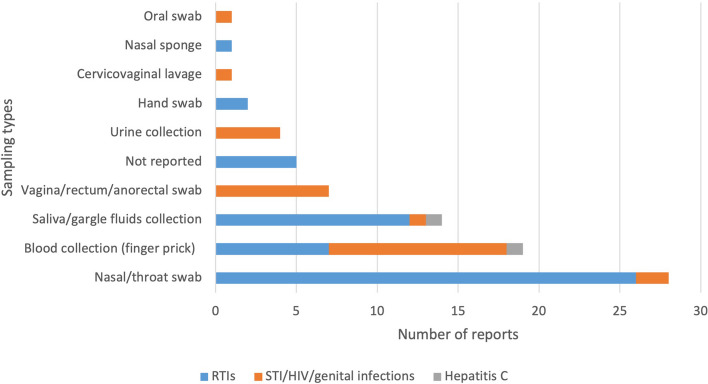
Types of self-sampling approaches. *There were 13 reports that investigated multiple types of sampling approaches; hence, the total number of reports in this figure is higher than the 64 included reports Note: There were four reports that reported SS/ST in two types of settings and one report with three types of settings; hence, the total number of reports in this figure is higher than the 64 included reports

Multiple sampling approaches were studied (Fig. 3). For RTIs, SS involved: self-collecting saliva, gargle fluids or blood from a finger prick, and self-swabbing nose, throat, tongue, tonsils or hands (with wet wipes for hand microbiota). For STIs/HIV/genital infections, self-sampling involved blood, urine or oral fluid samples, and throat, rectal or vaginal self-swabbing. For hepatitis C, SS involved self-collecting an oral fluid sample [70] or a dried blood spot [84].

Studies that explored user views on different self-sampling approaches showed mixed results, mostly indicating preferences for less invasive and easier sampling approaches. For example, hand swabbing with a wet wipe for hand microbiota was preferred over saliva SS and nasal swabbing [95]. SS gargle fluid was preferred to nasopharyngeal swabs^5^ [96] and to SS saliva, nasal, pharyngeal, oropharyngeal or oral swabs [83]. This was due to the ease of sample collection and perceived test validity [83, 97]. Saliva pot was rated as the least ‘unpleasant or uncomfortable’, followed by a saliva sponge, then throat swabs [51]. SS saliva was preferred over nasal and oral swabbing [64], and most surveyed students in SEND schools and respondents in a prison preferred saliva sampling to swabs [75]. Nasal self-swab was preferred over oropharyngeal swab collected by healthcare professionals, and both were preferred over nasopharyngeal self-swabbing [98]. The reasons for preferring nasal self-swabbing included: experiencing it as more ‘pleasant’, shorter response time, easy to perform, and no need to travel to the test centre; in contrast, the professionally-collected throat swab was preferred because of being perceived as more accurate and comfortable than nose sampling, and feeling more comfortable with a professional performing the test [98]. SS saliva and nasal/throat self-swabbing was less challenging than SS blood [53, 56, 73] and genital self-sampling [50, 54]. Oral fluid collection was preferred to blood sampling by health professionals [53], though researchers found that saliva testing led some participants to believe that HIV could be transmitted through saliva [53]. However, overall all SS approaches were generally considered acceptable by adults and children [51, 82, 95].

#### Perceived accuracy of and trust in self-sampling/self-testing technology

Across infections, surveys found that most (68%−96%) respondents believed that SS/ST was accurate and were confident in and trusted the test result [48, 55, 59, 62, 64, 66, 67]. However, qualitative studies highlighted that some participants felt uncertain about the accuracy of the tests and results, particularly for SARS-CoV-2 [67, 79, 82, 89], chlamydia [54], and among those testing positive for HIV [88]. Concerns about test accuracy was also among reasons provided by those who rejected SS/ST [68, 79, 80, 82]. Participants wanted more information about false positive COVID-19 test results [67, 79]. Some perceived the benefits of SS/ST to outweigh questions about false negatives [67, 76]. A UKHSA report on COVID-19 testing in SEND schools found that some interviewees perceived saliva SS to be more accurate than self-swabbing [75]. Participants trusted the blood-based test more than the oral swab for hepatitis C [70]. Moreover, trusting the study’s institution (for COVID-19 SS/ST) [82] and test distributor (for HIV SS/ST) [73] appeared to be enablers of SS/ST.

#### User instructions and training

Many reports (across infection/sampling types) mentioned users receiving written and pictorial instructions, and sometimes online videos, supervision, training or assistance [47, 51, 55, 59, 63, 67, 70, 75, 99–105]. Across infection/sampling types, surveys found that most (82–100%) respondents thought they understood SS/ST instructions [47, 47, 49, 51, 52, 56, 60, 61, 63, 68, 71, 73, 77, 84, 93, 97, 99, 101, 103, 106, 107]. Qualitative studies reported that clear instructions enabled SS/ST [52, 56, 66, 73, 82], and providing pictorial and video instructions [73, 82] and training [67, 89] were appreciated, useful and helped increase self-efficacy. Some participants also reported that practice/repetition helped shift their perceptions of SS/ST from annoying/scary to easy [87, 95]. However, some reported not understanding the instructions for SS/ST for STIs [86] or finding the instructions for HIV SS/ST unclear [88]. Few participants failed to self-sample for COVID-19 due to not understanding the instructions [68, 82]. Two UKHSA reports described developing support for blind and partially sighted (BPS) people when SS for PCR tests [105] and ST with LFDs [104] for COVID-19. BPS people reported positive experiences of receiving verbal instructions and guidance through live video calls and many felt that they would be unable to complete SS/ST without assistance.

#### Ease of use and difficulties obtaining samples

Across infections/sampling types, survey respondents scored the ease of SS/ST highly (42.3–98.5%) [48, 49, 52, 55–57, 59, 60, 62, 63, 68–73, 84, 93, 99–103, 106, 108]. This was reflected in qualitative studies, with participants describing SS/ST and using the equipment as easy and straightforward [51–54, 56, 64, 70, 73, 82, 85, 86, 88, 95]. Even among parents/guardians who collected swabs for COVID-19 testing from their children under 5 years old, most (75–77%) found the mini-tip and standard swabs not challenging to use [109]. However, the success rate for nose and throat swabbing was higher for mini-tip (84%) than standard (68%) swabs [109].

Challenges in performing SS/ST largely related to obtaining sufficient samples for SARS-CoV-2 [68, 81] and STIs [72]. Participants reported difficulties producing enough saliva ‘on demand’ [51, 64], collecting samples from children [65, 95], collecting enough blood for HIV testing [73], and finding a comfortable position for the anorectal swab for chlamydia testing [50]. Some reported overwhelming complexity of COVID-19 SS/ST [82], being unsure if they had touched the surrounding area when self-swabbing a throat [51], and problems with equipment for HIV SS: using vials [73], lancets [56, 88], and correctly inserting the test stick into the pot containing reagent [52]. In some studies, users’ uncertainty about correctly performing the SS/ST was emphasised [49, 86, 91]. In one COVID-19 study, participants reported concern about potentially putting others at risk if they performed the test incorrectly [79]. Despite concerns about the ‘tricky’ ST procedure, users’ still preferred to self-collect blood for HIV testing from a finger prick than through a venepuncture [49]. Difficulties with SS/ST, particularly blood sampling, and damaging the test in the process were among the reasons reported for failing to provide self-collected samples [49, 68, 82, 83].

Other reported challenges related to returning samples for COVID-19 testing [66], concerns that samples for STI/HIV testing may be lost in the post [73, 86], or the inconvenience of having to hand in the samples for chlamydia testing [50]. However, participants were also happy to receive and return throat swabs and saliva samples using postal services [51]. BPS people found the SS/ST for COVID-19 procedures (both PCR [105] and LFD [104]) challenging to perform independently, even with live video assistance. The studies recommended that BPS people use hands-free cameras for video assistance and that the testing kit packaging be improved.

#### Discomfort when self-sampling

Discomfort or pain were reported by some users, although SS/ST were still acceptable. SS/ST was often considered painless and not invasive [53, 64] and generally good [87].

For RTIs, most (82%) adults found nasal self-swabbing as ‘unproblematic’ and 18% as ‘only briefly unpleasant’ [61], and 58% reported no discomfort and 94% no pain [95]. However, when collecting swabs from children, fewer respondents reported it as ‘unproblematic’ and 52% as briefly unpleasant [61]. More parents/guardians reported ‘moderate, severe and extreme discomfort’ and fewer reported ‘no pain’ when collecting samples in children under five compared to children over five [95]. A third of respondents reported swabbing child’s nose only as they became too uncomfortable/distressed to continue to swab their throat [109]. Among students ST for COVID-19, 69.2% reported experiencing fear during the procedure [81]. Discomfort was among the most frequently cited concerns about COVID-19 testing by school and college pupils [76]. For STIs/HIV, surveys found that only 26.3–45.6% of respondents who performed blood-based [49, 71] and 16.7% who performed cervicovaginal SS [57] found it unpleasant.

Qualitative studies on COVID-19 also found that some experienced SS as unpleasant and uncomfortable [51, 66, 67, 87, 89], especially by younger children [95]. Nasal/throat self-swabbing caused feeling of gagging and vomiting [51, 85], self-swabbing a sore throat allowed users to control the swab and gag reflex [51]. Some (across infections) reported other negative feelings: a sense of disgust for saliva sampling [95] and gargle tests [82], fear of needles for blood testing [66, 73, 88], inconvenience when fasting before gargle/saliva sampling [64, 82, 95], or embarrassment at performing an anorectal swab for chlamydia was also reported [50]. Some participants reported the discomfort swabbing anus [50], fear of self-sampling blood [73], and concern over child’s distress along [78] among the reasons for declining SS/ST. Some described a sense of anxiety about SS/ST for chlamydia [50] and HIV [56] without healthcare professional’s supervision and advice and in ways disconnected from official healthcare pathways; this was found to be especially relevant for infrequent HIV testers [88] and when testing positive for chlamydia [54].

#### Obtaining and interpreting test results

Across infections, self-test results were available to users within 10–30 min. Users wanted the results to be communicated quickly [85, 86]. Waiting for COVID-19 ST results was experienced as anxiety-inducing [64, 82], more so than waiting for the HIV ST result in the clinic [56, 88], and the rapidity of the results helped reduce anxiety [56]. For hepatitis C ST, some participants did not understand that they had to time the test and read the results within a reading window [70].

When SS (for COVID-19 and HIV), participants found it convenient to access the results digitally [73, 82] or have them sent home by post [73], but the option of collecting the results from the clinic was also acceptable [53]. However, some were unclear how the results of COVID-19 testing were going to be communicated [66, 75, 82].

Across infections, surveys found that most (88.5–100%) respondents reported perceived ease of and confidence in interpreting the results [47, 59, 63, 67, 68, 92, 99, 101, 103, 106]. Two qualitative reports also mentioned that participants found it easy to read ST results [52, 56]. However, a minority of respondents indicated that the disadvantages of SS/ST for SARS-CoV-2 were that ‘*you have to interpret the result yourself*’ and ‘*not having the emotional and/or logistical support to read the result*’ [59].

#### Acting on self-test results

There was limited evidence on how people behaved (or reported to behave) following SS/ST and the extent to which they followed relevant guidance or requirements. When ST for SARS-CoV-2, some participants were unsure about acting on positive results [85] and confused about whether one should still self-isolate alongside daily ST [79]. A survey of public behaviours, reported by the UKHSA [74], showed that at most 49% of the surveyed people who had taken an LFD COVID-19 test reported/registered the results. The most common reasons for not registering the results were ‘*not feeling the need to*’ and ‘*testing negative*’ [74]. Furthermore, the report showed that a vast majority of those who tested positive for COVID-19 stayed at home after testing (92% of those who registered the result, 87% of those who did not), warned others (e.g., family, friends, employers) and continued testing until receiving a negative result [74]. For chlamydia testing, one study reported that some participants felt unsure what to do if they received positive results and criticised the lack of the possibility of counselling [86].

### Comparison with non-European studies

Of the 130 studies conducted outside of Europe, 59 were from countries in Africa, 46 from North America, 18 from Asia, five from South America, and two from Australia (Table 3). Similarly to European studies, most non-European studies involved surveys (*n* = 72), followed by mixed (*n* = 31) and qualitative (*n* = 27) methods. While most European studies focused on RTIs, the vast majority of non-European studies focused on STIs/HIV/genital infections (*n* = 103), and only 19 on RTIs and five on hepatitis C. Two studies focused on UTIs and one on malaria, which were absent among European studies. Supplementary Table S6 summarises the main characteristics and findings of these studies.

When exploring the main enablers and barriers to SS/ST reported in non-European studies, we found that most factors affecting the acceptability of SS/ST appear to be similar across European and non-European studies (Supplementary Table S7). However, as most non-European studies focused on STIs/HIV, the findings particularly highlighted the benefits of privacy and confidentiality of SS/ST (especially in Africa where the stigma of STIs/HIV remains strong), and their role in promoting empowerment and autonomy in managing one’s health (e.g., when negotiating condom use). Other, unique enablers included social influences (e.g., seeing others testing, impact on relationships by sharing ST experiences, peer support), and an ability to safely dispose of ST kits. Among barriers, non-European studies (particularly in Africa) highlighted a lack of awareness of not only SS/ST but also infections. In addition to concerns about confidentiality (reports globally), they also highlighted mistrust regarding the privacy of test results and protected characteristics. Studies also uniquely discussed a lack of (psychological) support, disconnection from health services, and language barriers. The broader global literature on SS/ST also explored strategies and factors related to the distribution of SS/ST kits, which can act as barriers and facilitators, although outside of our scope.

## Discussion

Overall, we found consistently high levels of acceptability of SS/ST across infection types, sampling/testing approaches, settings, populations, and countries. We identified many perceived benefits of SS/ST that motivated people to perform it and factors that enabled and facilitated SS/ST. However, some participants/respondents also reported challenges to SS/ST. Although these barriers did not seem to impede SS/ST uptake, or did so for a minority of participants, they indicate areas that may need addressing for effective implementation of SS/ST.

Our findings are in line with other reviews on STIs/HIV, which also found high acceptability and similar barriers to and enablers of SS/ST across the world [23–30, 38]. We focused on those with experience of SS/ST, but those who declined SS/ST reported largely similar barriers. Reviewed studies highlighted several perceived benefits of SS/ST, which may have contributed to the high levels of acceptability. For example, people wanted to know whether they were infected or not to inform their behaviours, and SS/ST enabled them to do that in accessible, convenient and private ways. This finding is supported by the theoretical frameworks on patient ST [9, 110] and, more broadly, the uptake of healthcare technologies [12] which argue that the technology – to be used – needs to be (perceived as) beneficial/useful to users and needs engaged and empowered users. It also illustrates the growing role of ST and health technology in self-care and self-management beyond chronic diseases [9]. Moreover, we found that participants’ interest in SS/ST transcended individual benefits by being also socially-motivated; for example, to protect others from the infection, contribute to research and public health initiatives, as ‘good’ citizenship, and due to interest in promoting health innovations. Importantly, we did not find evidence that the acceptability and benefits of SS/ST were restricted to privileged, empowered and autonomous populations. Despite limited evidence on health inequalities, we found that SS/ST was also acceptable and beneficial among typically underserved and stigmatised populations (e.g., prisoners, transgender people, sexual or ethnic minorities, BPS people); especially if SS/ST was provided in more targeted ways to address specific barriers, e.g., to anonymity or usability.

More ambivalent evidence concerned users’ preferences. Less invasive SS seemed preferred (such as gargling preferred over swabbing over blood collection), influenced by the perceived ease of use and discomfort. We found mixed preferences for nose or throat self-swabbing, likely dependent on individual experiences of discomfort. However, these preferences were expressed in the context of high overall acceptability and uptake of all types of SS/ST. Similar levels of discomfort were also reported in the reviewed studies for samples collected by healthcare professionals. The discomfort did not seem to preclude SS/ST [75], except when collecting samples from children under five [109] – suggesting that different preferences did not necessarily lead to different testing behaviours.

There were also mixed views and preferences regarding settings of SS/ST and ways of receiving the results, depending on individual considerations of convenience, flexibility and the importance of support and advice for SS/ST and if testing positive. This suggests a need for multi-faceted delivery approaches to meet the needs of diverse users. A hybrid approach to offering the results (e.g., digitally, by telephone or in-person) might be also appropriate to mitigate against digital exclusion.

Providing clear instructions and training and practice/rehearsal enabled, and increased confidence in, SS/ST. Difficulties understanding the instructions and/or lack of training/practice could pose barriers to correctly performing SS/ST but that was found only for a minority of study participants. For some people (e.g., with visual impairments), additional assistance was needed [104, 105]. Following guidance for the design of SS/ST packs and instructions, such as the one for STIs and blood borne viruses [111], is important to ensure that likely barriers to the correct performance of SS/ST are minimised. Moreover, increasing users’ trust in the accuracy of SS/ST was suggested as important to promoting uptake and adherence to the recommended behaviours following the SS/ST results. We found little information in the synthesised reports on whether participants were informed about test accuracy, despite the evidence suggesting high sensitivity and specificity for most assessed tests [14, 15, 17–20]. Identifying ways to optimise the delivery and design of SS/ST packs (e.g., by using behavioural science and qualitative approaches) could further increase acceptability and uptake [90, 112, 113].

### Implications

The main implication for practice is that SS/ST for infections can be considered a broadly acceptable strategy for a range of respiratory and sexually-transmitted infections. Hence, in principle, it could be considered for implementation as part of routine care or in response to a health emergency. Implementation would require addressing potential barriers and challenges, such as clarifying to users the benefits and accuracy of SS/ST, ensuring low risks of infection transmission in public testing sites, offering clear instructions on how to correctly perform SS/ST and interpret ST results (including user-tested written, pictorial and video formats) with an opportunity to practise, offering different SS/ST settings and ways of providing test results, and providing clear instructions on how to act on results and support for those testing positive. Where possible, less invasive and easier-to-use tests should be offered, especially if their performance is comparable to more invasive (e.g., blood) tests.

In Europe, the main evidence gap concerns SS/ST for UTIs (no studies identified) and RTIs other than SARS-CoV-2. Most RTI studies were on SARS-CoV-2 and conducted during the pandemic (mostly as part of government-mandated free testing programmes) and in limited populations and settings (mostly university and school staff and students). More research on SS/ST for RTIs is needed as motivations for, and acceptability of, SS/ST for RTIs might differ in a non-pandemic context, particularly when government guidance removes the requirement of testing and requires test purchasing. Outside of health emergencies, other factors (such as seasonality, infection type/severity), might be important. Evidence is also limited on costs of, and willingness to pay for, SS/ST and users’ actions following SS/ST, especially in naturalistic settings. Understanding the impacts of SS/ST on users (such as self-care, help-seeking behaviours, and health outcomes) and healthcare professionals and services (such as the impact on demand/workload) is important to ensure that SS/ST does not lead to negative unintended consequences. More evidence would be useful regarding SS/ST and health inequities and comparing views and experiences of SS/ST among different population groups, particularly those under-represented in research.

### Limitations

Our electronic searches, although comprehensive, might have missed some studies, particularly when reports referred only to ‘testing’ (even if self-performed). Due to the rapid/restricted nature of this review, we used strategies to manage the scope and workload that are accepted in rapid/restricted reviews [42]. First, screening and data extraction were conducted by a single researcher, with 25% of excluded citations double-screened and data extracted verified during analysis. It is unlikely that additional studies would lead to significantly different findings in light of considerable consistency across the synthesised reports. Second, we searched for studies from 2014 because SS/ST technology has developed considerably in the last 10 years, and we expected most studies to be published in that period. Third, we also prioritised for detailed data extraction and synthesis a sub-set of eligible studies conducted in Europe. However, as part of due diligence, we examined the non-European studies (albeit on a surface level and without quality appraisal). We found the findings to be likely comparable across the world while identifying the apparent differences – these may relate to variation in the infections studied and/or the national contexts. The quality of the synthesis depends on the methodological and reporting quality of included studies, which vary. However, most studies were thought to be of good quality.

## Conclusions

This review synthesised a large body of evidence on user acceptability of SS/ST, and factors influencing the acceptability and uptake of SS/ST, across different infections, sampling approaches, settings and populations. The reviewed evidence shows that, notwithstanding different enablers and barriers, members of the public who performed self-sampling/self-testing for infections considered it acceptable, easy to perform, and beneficial – irrespective of infection or sampling types. Establishing acceptability and effectiveness are the first steps towards implementing new public health strategies. The consistently high levels of acceptability support the potentially more widespread self-sampling/self-testing interventions in routine healthcare, with a greater focus on self- and home care. It also supports the importance of considering self-sampling/self-testing in planning and preparedness for future pandemics, while recognising the need for devolved delivery of services to reach wider populations.

## Supplementary Information

Supplementary Material 1: Supplementary Document S1. Further details of the search strategy. Supplementary Table S1. Additional details and SS/ST approaches in studies conducted in Europe. Supplementary Table S2. Summary of quantitative findings from peer-reviewed studies conducted in Europe. Supplementary Table S3. Summary of qualitative findings from peer-reviewed studies conducted in Europe. Supplementary Table S4. Summary of findings from non-peer-reviewed reports of studies conducted in the UK. Supplementary Table S5. Quality appraisal of peer-reviewed studies conducted in Europe. Supplementary Table S6. Characteristics of eligible studies conducted outside of Europe. Supplementary Table S7. Summary of enablers and barriers to self-sampling/self-testing reported in studies outside of Europe.

## Data Availability

All data generated or analysed during this study are included in this published article and its supplementary files.

## Abbreviations

BPS: Blind and Partially Sighted
HIV: Human Immunodeficiency Virus
HCPs: Healthcare Professionals
HCV: Hepatitis C virus
HPV: Human papillomavirus
LFDs: Lateral Flow Devices
MMAT: Mixed Methods Appraisal Tool
PCR: Polymerase Chain Reaction
RTIs: Respiratory Tract Infections
SARS-CoV-2: Severe Acute Respiratory Syndrome Coronavirus 2
SEND: Special Educational Needs and Disabilities
SS: Self-Sampling
ST: Self-Testing
STIs: Sexually Transmitted Infections
UK: United Kingdom
UKHSA: UK Health Security Agency
WHO: World Health Organisation

## Footnotes

1 We used the MMAT, instead of three separate quality assessment tools proposed in the protocol, as it can be applied across different study designs.

2 Due to the rapid, restricted nature of this review, we were unable to take the same approach to detailed data extraction, synthesis and quality appraisal as we did for the European studies. We did not assess the quality of the non-European studies. This post-hoc analysis was only exploratory.

3 For brevity, we subsequently refer to qualitative studies and surveys to mean studies that reported relevant qualitative/survey findings, even if some might be mixed-methods studies. Where we don’t specify study/data type, we mean that the synthesised evidence comes from across study/data types.

4 For brevity, where we refer to STIs we mean a range of STIs, genital infections and HIV. Where relevant to the specific type of infection, we refer to that infection (e.g., HIV).

5 Nose and throat self-swabbing differs from deeper nasopharyngeal swabbing usually done by healthcare professionals. In this study, the authors specifically investigated self-swabbing the oropharynx or nasopharynx and compared it with samples collected by healthcare professionals.
