# Supplementary material for: Acceptability of self-sampling and self-testing for infections: a rapid systematic review on public users’ views
Source: BMC Public Health. 2025 Feb 20;25:695. doi: 10.1186/s12889-025-21773-w (PMC11841015; doi:10.1186/s12889-025-21773-w)
Supplement: Supplementary file 1 — Supplementary Material 1: Supplementary Document S1. Further details of the search strategy. Supplementary Table S1. Additional details and SS/ST approaches in studies conducted in Europe. Supplementary Table S2. Summary of quantitative findings from peer-reviewed studies conducted in Europe. Supplementary Table S3. Summary of qualitative findings from peer-reviewed studies conducted in Europe. Supplementary Table S4. Summary of findings from non-peer-reviewed reports of studies conducted in the UK. Supplementary Table S5. Quality appraisal of peer-reviewed studies conducted in Europe. Supplementary Table S6. Characteristics of eligible studies conducted outside of Europe. Supplementary Table S7. Summary of enablers and barriers to self-sampling/self-testing reported in studies outside of Europe. [file 12889_2025_21773_MOESM1_ESM.pdf]

Acceptability of self-sampling and self-testing for infections: A rapid systematic review on public users' views

Supplementary Files

Contents

List of abbreviations ... **2**

Supplementary Document S1: Further details of the search strategy ... **3**

Electronic database searches: ... 3

Other online searches of literature ... 7

Searching for literature via expert networks ... 8

Supplementary Table S1: Additional details and self-sampling/self-testing approaches in studies conducted in Europe ... **9**

Part A – UK studies ... 9

Part B – Other European studies ... 16

Supplementary Table S2: Summary of quantitative findings from peer-reviewed studies conducted in Europe ... **26**

Supplementary Table S3: Summary of qualitative findings from peer-reviewed studies conducted in Europe ... **33**

Supplementary Table S4: Summary of findings from included non-peer-reviewed reports ... **42**

Supplementary Table S5: Quality appraisal of peer-reviewed studies conducted in Europe ... **46**

Part A – Quantitative descriptive studies ... 46

Part B – Mixed-methods studies ... 48

Part C – Qualitative studies ... 49

Supplementary Table S6: Key characteristics of eligible studies conducted outside of Europe ... **51**

Supplementary Table S7: Summary of enablers and barriers to self-sampling/self-testing reported in studies outside of Europe ... **74**

Part A - Enablers ... 74

Part B - Barriers ... 77

References ... **81**

List of abbreviations

AMR – Antimicrobial Stewardship,
BPS – Blind and Partially Sighted,
CT – Chlamydia Trachomatis,
DBS – Dry-blood spot,
LFIA – Lateral Flow Immunoassays,
LFD – Lateral Flow Device,
LFT – Lateral Flow Test,
HBV – Hepatitis B Virus,
HBC – Hepatitis C Virus,
HEI – Higher Education Institution,
HIV – Human Immunodeficiency Virus,
HPRU – Health Protection Research Unit,
MM – Mixed Methods,
MSM – Men who have Sex with Men,
NG – Neisseria Gonorrhoeae,
MG – Mycoplasma Genitalium,
NIHR – National Institute for Health and Social Care Research,
N/R – Not Reported,
PCR – Polymerase Chain Reaction,
PWID – People Who Inject Drugs,
RADTs – Rapid Antigen Detection Tests,
RTI – Respiratory Tract Infection(s),
RT-PCR – Reverse Transcription Polymerase Chain Reaction,
SEND – Special Educational Needs and Disabilities,
SS – Self-sampling,
ST – Self-testing/Self-test,
STI – Sexually Transmitted Infection(s),
TG – Transgender,
TV – Trichomonas Vaginalis,
UK – United Kingdom,
UKHSA – UK Health Security Agency

Supplementary Document S1: Further details of the search strategy

We searched for literature (including preprints and theses) in the following ways:

Electronic database searches:

We searched the following databases from inception to 15/12/2023:

1) Medline (OvidSP) [1946-present],
2) Embase (OvidSP) [1974-present],
3) PsycINFO (OvidSP) [1806-present],
4) CINAHL (EBSCOHost) [1982-present],
5) Science Citation Index & Social Science Citation Index (Web of Science Core Collection) [1900-present].

The search strategy contained title, abstract, author keyword and subject headings for our key concepts - self-testing, acceptability and qualitative or survey research methods.

The searches were limited to 2014 onwards, no language limits were applied.

The search strategy was developed in Medline and adapted to the other databases with the aid of Polyglot (https://sr-accelerator.com/#/polyglot).

Results were de-duplicated in Deduplicator (https://sr-accelerator.com/#/deduplicator).

Results were exported to Microsoft Excel for screening.

SEARCH STRATEGY USED IN MEDLINE

1	self-testing/
2	Self Care/ and Specimen Handling/
3	Self Care/ and COVID-19 Testing/
4	COVID-19 Testing/ and Specimen Handling/
5	COVID-19 Testing/ and exp Cell phone/
6	Specimen Handling/ and exp Cell phone/
7	(self* adj (test* or sampl*)).ti,ab,kf.
8	((home or "at home" or home based) adj (test* or sampl*)).ti,ab,kf.
9	((self* adj collect*) and (sampl* or specimen* or biospecimen* or swab*)).ti,ab,kf.
10	((home* adj collect*) and (sampl* or specimen* or biospecimen* or swab*)).ti,ab,kf.
11	((home* or self*) adj5 lateral flow).ti,ab,kf.
12	1 or 2 or 3 or 4 or 5 or 6 or 7 or 8 or 9 or 10 or 11
13	exp Attitude to Health/
14	Emotions/
15	px.fs.
16	(acceptance or acceptab* or feasib* or usable or usabil* or complian* or noncomplian* or adhere* or nonadhere*).ti,ab,kf.
17	(barrier* or obstac* or challeng* or enabl* or facilitat* or empower* or engage*).ti,ab,kf.

18	(experience? or attitud* or view* or perception? or perspective? or understanding or comprehen* or feeling* or emotion*).ti,ab,kf.
19	13 or 14 or 15 or 16 or 17 or 18
20	Surveys and Questionnaires/
21	focus groups/ or interviews as topic/
22	grounded theory/ or exp qualitative research/
23	qualitative.ti,kf. or (qualitative adj3 (research or stud* or data or analys* or interpret*)).ab.
24	(focus group? or interview* or survey* or questionnaire*).ti,ab,kf.
25	(grounded theory or ethnogra* or hermeneutic* or phenomenolog* or themes or thematic or mixed method* or multi* method*).ti,ab,kf.
26	20 or 21 or 22 or 23 or 24 or 25
27	12 and 19 and 26
28	limit 27 to yr="2014 -Current"

<u>SEARCH STRATEGY USED IN EMBASE:</u>

1	self-testing/
2	(Self Care/ or Home Quarantine/) and (*sampling/ or airway sampling/ or specimen handling/ or exp swabbing/ or urine sampling/)
3	(Self Care/ or Home Quarantine/) and COVID-19 Testing/
4	COVID-19 Testing/ and (*sampling/ or airway sampling/ or specimen handling/ or exp swabbing/)
5	COVID-19 Testing/ and exp mobile phone/
6	(*sampling/ or airway sampling/ or specimen handling/ or exp swabbing/ or urine sampling/) and exp mobile phone/
7	(self* adj (test* or sampl*)).ti,ab,kf.
8	((home or "at home" or home based) adj (test* or sampl*)).ti,ab,kf.
9	((self* adj collect*) and (sampl* or specimen* or biospecimen* or swab*)).ti,ab,kf.
10	((home* adj collect*) and (sampl* or specimen* or biospecimen* or swab*)).ti,ab,kf.
11	((home* or self*) adj5 lateral flow).ti,ab,kf.
12	1 or 2 or 3 or 4 or 5 or 6 or 7 or 8 or 9 or 10 or 11
13	attitude to health/ or attitude to illness/ or exp patient attitude/
14	Emotion/ or emotionality/
15	program acceptability/
16	(acceptance or acceptab* or feasib* or usable or usabil* or complian* or noncomplian* or adhere* or nonadhere*).ti,ab,kf.
17	(barrier* or obstac* or challeng* or enabl* or facilitat* or empower* or engage*).ti,ab,kf.
18	(experience? or attitud* or view* or perception? or perspective? or understanding or comprehen* or feeling* or emotion*).ti,ab,kf.
19	13 or 14 or 15 or 16 or 17 or 18
20	exp questionnaire/ or open ended questionnaire/ or exp structured questionnaire/
21	exp interview/ or exp observational method/ or multimethod study/
22	grounded theory/ or exp qualitative research/ or thematic analysis/
23	qualitative.ti,kf. or (qualitative adj3 (research or stud* or data or analys* or interpret*)).ab.

24	(focus group? or interview* or survey* or questionnaire*).ti,ab,kf.
25	(grounded theory or ethnogra* or hermeneutic* or phenomenolog* or themes or thematic or mixed method* or multi* method*).ti,ab,kf.
26	20 or 21 or 22 or 23 or 24 or 25
27	12 and 19 and 26
28	limit 27 to yr="2014 -Current"

SEARCH STRATEGY USED IN PSYCINFO:

1	(self* adj (test* or sampl*)).ti,ab,id.
2	((home or "at home" or home based) adj (test* or sampl*)).ti,ab,id.
3	((self* adj collect*) and (sampl* or specimen* or biospecimen* or swab*)).ti,ab,id.
4	((home* adj collect*) and (sampl* or specimen* or biospecimen* or swab*)).ti,ab,id.
5	((home* or self*) adj5 lateral flow).ti,ab,id.
6	1 or 2 or 3 or 4 or 5
7	attitudes/ or exp health attitudes/ or exp compliance/
8	exp Emotions/
9	technology acceptance/
10	(acceptance or acceptab* or feasib* or usable or usabil* or complian* or noncomplian* or adhere* or nonadhere*).ti,ab,id.
11	(barrier* or obstac* or challeng* or enabl* or facilitat* or empower* or engage*).ti,ab,id.
12	(experience? or attitud* or view* or perception? or perspective? or understanding or comprehen* or feeling* or emotion*).ti,ab,id.
13	7 or 8 or 9 or 10 or 11 or 12
14	surveys/ or questionnaires/
15	mixed methods research/
16	exp qualitative methods/
17	(focus group or interview or metasynthesis or qualitative study).md.
18	qualitative.ti,id. or (qualitative adj3 (research or stud* or data or analys* or interpret*)).ab.
19	(focus group? or interview* or survey* or questionnaire*).ti,ab,id.
20	(grounded theory or ethnogra* or hermeneutic* or phenomenolog* or themes or thematic or mixed method* or multiple method*).ti,ab,id.
21	14 or 15 or 16 or 17 or 18 or 19 or 20
22	6 and 13 and 21
23	limit 22 to yr="2014 -Current"

SEARCH STRATEGY USED IN CINAHL:

S16	S14 OR S15

S15	(TI qualitative) OR ((AB qualitative) N3 ((AB research) OR (AB stud*) OR (AB data) OR (AB analys*) OR (AB interpret*))) OR ((TI "focus group#" OR AB "focus group#" OR SU "focus group#") OR (TI interview* OR AB interview* OR SU interview*) OR (TI survey* OR AB survey* OR SU survey*) OR (TI questionnaire* OR AB questionnaire* OR SU questionnaire*)) OR ((TI "grounded theory" OR AB "grounded theory" OR SU "grounded theory") OR (TI ethnogra* OR AB ethnogra* OR SU ethnogra*) OR (TI hermeneutic* OR AB hermeneutic* OR SU hermeneutic*) OR (TI phenomenolog* OR AB phenomenolog* OR SU phenomenolog*) OR (TI themes OR AB themes OR SU themes) OR (TI thematic OR AB thematic OR SU thematic) OR (TI "mixed method*" OR AB "mixed method*" OR SU "mixed method*") OR (TI "multiple method*" OR AB "multiple method*" OR SU "multiple method*"))
S14	(MH "Qualitative Studies+") OR (MH "Focus Groups") OR (MH "Interviews+") OR (MH "Observational Methods+") OR (MH "Surveys") OR (MH "Thematic Analysis") OR (MH "Meta Synthesis")
S13	S9 OR S10 OR S11 OR S12
S12	((TI acceptance OR AB acceptance OR SU acceptance) OR (TI acceptab* OR AB acceptab* OR SU acceptab*) OR (TI feasib* OR AB feasib* OR SU feasib*) OR (TI usable OR AB usable OR SU usable) OR (TI usabil* OR AB usabil* OR SU usabil*) OR (TI complian* OR AB complian* OR SU complian*) OR (TI noncomplian* OR AB noncomplian* OR SU noncomplian*) OR (TI adhere* OR AB adhere* OR SU adhere*) OR (TI nonadhere* OR AB nonadhere* OR SU nonadhere*)) OR ((TI barrier* OR AB barrier* OR SU barrier*) OR (TI obstac* OR AB obstac* OR SU obstac*) OR (TI challeng* OR AB challeng* OR SU challeng*) OR (TI enabl* OR AB enabl* OR SU enabl*) OR (TI facilitat* OR AB facilitat* OR SU facilitat*) OR (TI empower* OR AB empower* OR SU empower*) OR (TI engage* OR AB engage* OR SU engage*)) OR ((TI experience# OR AB experience# OR SU experience#) OR (TI attitud* OR AB attitud* OR SU attitud*) OR (TI view* OR AB view* OR SU view*) OR (TI perception# OR AB perception# OR SU perception#) OR (TI perspective# OR AB perspective# OR SU perspective#) OR (TI understanding OR AB understanding OR SU understanding) OR (TI comprehen* OR AB comprehen* OR SU comprehen*) OR (TI feeling* OR AB feeling* OR SU feeling*) OR (TI emotion* OR AB emotion* OR SU emotion*))
S11	MW "pf"
S10	(MH "Emotions")
S9	(MH "Attitude to Health+") OR (MH "Attitude") OR (MH "Psychosocial Aspects of Illness")
S8	S1 OR S2 OR S3 OR S4 OR S5 OR S6 OR S7
S7	((TI self* OR AB self* OR SU self*) W1 ((TI test* OR AB test* OR SU test*) OR (TI sampl* OR AB sampl* OR SU sampl*))) OR (((TI home OR AB home OR SU home) OR (TI "at home" OR AB "at home" OR SU "at home") OR (TI "home based" OR AB "home based" OR SU "home based")) W1 ((TI test* OR AB test* OR SU test*) OR (TI sampl* OR AB sampl* OR SU sampl*))) OR (((TI self* OR AB self* OR SU self*) W1 (TI collect* OR AB collect* OR SU collect*)) AND ((TI sampl* OR AB sampl* OR SU sampl*) OR (TI specimen* OR AB specimen* OR SU specimen*) OR (TI biospecimen* OR AB biospecimen* OR SU biospecimen*) OR (TI swab* OR AB swab* OR SU swab*))) OR (((TI home* OR AB home* OR SU home*) W1 (TI collect* OR AB collect* OR SU collect*)) AND ((TI sampl* OR AB sampl* OR SU sampl*) OR (TI specimen* OR AB specimen* OR SU specimen*) OR (TI biospecimen* OR AB biospecimen* OR SU biospecimen*) OR (TI swab* OR AB swab* OR SU swab*))) OR (((MW home*) OR (MW self*)) N5 (MW "lateral flow"))
S6	(MH "Cellular Phone+") AND (MH "Specimen Handling")
S5	(MH "COVID-19 Testing") AND (MH "Cellular Phone+")
S4	(MH "COVID-19 Testing") AND (MH "Specimen Handling")
S3	(MH "Self Care") AND (MH "COVID-19 Testing")
S2	(MH "Self Care") AND (MH "Specimen Handling")
S1	(MH "Self-Testing")

SEARCH STRATEGY USED IN WEB OF SCIENCE:

1	TS=(self* NEAR/0 (test* OR sampl*)) OR TS=((home OR "at home" OR "home based") NEAR/0 (test* OR sampl*)) OR TS=((self* NEAR/0 collect*) AND (sampl* OR specimen* OR biospecimen* OR swab*)) OR TS=((home* NEAR/0 collect*) AND (sampl* OR specimen* OR biospecimen* OR swab*)) Editions: WOS.SCI,WOS.SSCI
2	TS=(acceptance OR acceptab* OR feasib* OR usable OR usabil* OR complian* OR noncomplian* OR adhere* OR nonadhere*) OR TS=(barrier* OR obstac* OR challeng* OR enabl* OR facilitat* OR empower* OR engage*) OR TS=(experience$ OR attitud* OR view* OR perception$ OR perspective$ OR understanding OR comprehen* OR feeling* OR emotion*) Editions: WOS.SCI,WOS.SSCI
3	TI=qualitative OR AB=(qualitative NEAR/3 (research OR stud* OR data OR analys* OR interpret*)) OR TS=("focus group$" OR interview* OR survey* OR questionnaire*) OR TS=("grounded theory" OR ethnogra* OR hermeneutic* OR phenomenolog* OR themes OR thematic OR "mixed method*" OR "multiple method*") Editions: WOS.SCI,WOS.SSCI
4	#3 AND #2 AND #1 Editions: WOS.SCI,WOS.SSCI
5	#3 AND #2 AND #1 and 2023 or 2022 or 2021 or 2020 or 2019 or 2018 or 2017 or 2016 or 2015 or 2014 (Publication Years) Editions: WOS.SCI,WOS.SSCI
6	#2 AND #1 Editions: WOS.SCI,WOS.SSCI
7	#2 AND #1 and 2023 or 2022 or 2021 or 2020 or 2019 or 2018 or 2017 or 2016 or 2015 or 2014 (Publication Years) Editions: WOS.SCI,WOS.SSCI
8	#2 AND #1 and 2023 or 2022 or 2021 or 2020 or 2019 or 2018 or 2017 or 2016 or 2015 or 2014 (Publication Years) and Review Article (Document Types) Editions: WOS.SCI,WOS.SSCI

Other online searches of literature

We conducted **backward citation searches** by reviewing reference lists of included papers and of identified relevant recent reviews. The reviews included:

Anand, Apoorva, Fiorella Vialard, Aliasgar Esmail, Faiz Ahmad Khan, Patrick O'Byrne, Jean-Pierre Routy, Keertan Dheda, and Nitika Pant Pai. 'Self-Tests for COVID-19: What Is the Evidence? A Living Systematic Review and Meta-Analysis (2020–2023)'. *PLOS Global Public Health* 4, no. 2 (7 February 2024): e0002336. https://doi.org/10.1371/journal.pgph.0002336.

Harrison, Ciara, Daniel E. Lindholm, Andrew C. Steer, and Joshua Osowicki. 'A Systematic Review and Meta-Analysis of Upper Airway Swab Collection for Detection of Viral and Bacterial Pathogens by Individuals or Caregivers Compared to Health Care Workers'. *Journal of Clinical Microbiology* 59, no. 7 (18 June 2021): 10.1128/jcm.02304-20. https://doi.org/10.1128/jcm.02304-20.

Iyamu, Ihoghosa, Rodrigo Sierra-Rosales, Claudia S. Estcourt, Amy Salmon, Mieke Koehoorn, and Mark Gilbert. 'Differential Uptake and Effects of Digital Sexually Transmitted and Bloodborne Infection Testing Interventions among Equity-Seeking Groups: A Scoping Review'. *Sexually Transmitted Infections* 99, no. 8 (1 December 2023): 554–60. https://doi.org/10.1136/sextrans-2023-055749.

Kpokiri, Eneyi E, Gifty Marley, Weiming Tang, Noah Fongwen, Dan Wu, Sima Berendes, Bhavana Ambil, et al. 'Diagnostic Infectious Diseases Testing Outside Clinics: A Global Systematic Review and Meta-Analysis'. *Open Forum Infectious Diseases* 7, no. 10 (1 October 2020): ofaa360. https://doi.org/10.1093/ofid/ofaa360.

Kularadhan, Varsicka, Joscelyn Gan, Eric P. F. Chow, Christopher K. Fairley, and Jason J. Ong. 'HIV and STI Testing Preferences for Men Who Have Sex with Men in High-Income Countries: A Scoping Review'. *International Journal of Environmental Research and Public Health* 19, no. 5 (January 2022): 3002. https://doi.org/10.3390/ijerph19053002.

McGuire, Madison, Anna de Waal, Angela Karellis, Ricky Janssen, Nora Engel, Rangarajan Sampath, Sergio Carmona, Alice Anne Zwerling, Marta Fernandez Suarez, and Nitika Pant Pai. 'HIV Self-Testing with Digital Supports as the New Paradigm: A Systematic Review of Global Evidence (2010–2021)'. *eClinicalMedicine* 39 (1 September 2021). https://doi.org/10.1016/j.eclinm.2021.101059.

Peeling, Rosanna, and Samuel K. Sia. 'Lessons from COVID-19 for Improving Diagnostic Access in Future Pandemics'. *Lab on a Chip* 23, no. 5 (2023): 1376–88. https://doi.org/10.1039/D2LC00662F.

Perazzo, Hugo, Rodolfo Castro, Cristiane Villela-Nogueira, Marcos Torres, Samara Luiza Silva, Sandra W. Cardoso, Beatriz Grinsztejn, and Valdilea G. Veloso. 'Acceptability and Usability of Oral Fluid HCV Self-Testing for Hepatitis C Diagnosis: A Systematic Review and Meta-Analysis'. *Journal of Viral Hepatitis* 30, no. 11 (2023): 838–47. https://doi.org/10.1111/jvh.13876.

Qin, Yilu, Larry Han, Andrew Babbitt, Jennifer S. Walker, Fengying Liu, Harsha Thirumurthy, Weiming Tang, and Joseph D. Tucker. 'Experiences Using and Organizing HIV Self-Testing'. *AIDS* 32, no. 3 (28 January 2018): 371. https://doi.org/10.1097/QAD.0000000000001705.

Kurniawan, Samuel Johnson, Maria Mardalena Martini Kaisar, Helen Kristin, and Soegianto Ali. 'Comparable Performance of Antigen-Detecting Rapid Test by Healthcare Worker-Collected and Self-Collected Swabs for SARS-CoV-2 Diagnostic: A Systematic Review and Meta-Analysis'. *Reviews in Medical Virology* 34, no. 1 (2024): e2492. https://doi.org/10.1002/rmv.2492.

Spence, Tommer, Inès Kander, Julia Walsh, Frances Griffiths, and Jonathan Ross. 'Perceptions and Experiences of Internet-Based Testing for Sexually Transmitted Infections: Systematic Review and Synthesis of Qualitative Research'. *Journal of Medical Internet Research* 22, no. 8 (26 August 2020): e17667. https://doi.org/10.2196/17667.

Tsang, Nicole Ngai Yung, Hau Chi So, Ka Yan Ng, Benjamin J. Cowling, Gabriel M. Leung, and Dennis Kai Ming Ip. 'Diagnostic Performance of Different Sampling Approaches for SARS-CoV-2 RT-PCR Testing: A Systematic Review and Meta-Analysis'. *The Lancet Infectious Diseases* 21, no. 9 (1 September 2021): 1233–45. https://doi.org/10.1016/S1473-3099(21)00146-8.

We conducted forward citation searches of above reviews by using the 'cited by' function in Google Scholar.

We searched key terms (self-testing, testing at home, testing for infections) in Google Scholar and screened the first 10 pages of search results.

We reviewed the UK Government and UK Health Security Agency online reports on COVID-19/SARS-CoV-2 testing listed as a collection (COVID-19: testing initiative evaluation programme) available on https://www.gov.uk/government/collections/covid-19-testing-initiative-evaluation-programme.

Searching for literature via expert networks

We asked about potentially eligible studies experts in diagnostics and infectious diseases

- via direct contacts and networks of the members of the review team (UK),
- NIHR Health Protection Research Unit (HPRU) in AMR and cross-HPRU behavioural science network (UK),
- Society for Academic Primary Care (UK),
- General Practice Research on Infections Network (European network).

Supplementary Table S1: Additional details and self-sampling/self-testing approaches in studies conducted in Europe

Part A – UK studies

UK STUDIES						
**Author, year (reference), country, study design, infection**	**Details of the self-sampling (SS) & self-testing (ST)**	**Setting**	**User instructions, training & supervision**	**SS/ST number & frequency**	**Details related to SS/ST results**	**User instructions & actions after SS/ST**
Ahmed-Little 2016 (1), UK, MM, STI (HIV)	SS: Dry-blood-spot (DBS) testing via finger prick	Home	N/R but can be assumed unsupervised	Once	Participants could choose to have results returned via text message using an automated system, phone call from a clinician or a letter sent by first class.	Any positive/ reactive results were referred urgently to local sexual health services for confirmation via blood serum testing and specialist management.
Aiano 2021 (2), UK, MM, RTI (SARS-CoV-2)	SS: Oral fluid swabs (children); nose, throat and oral fluid swabs (adults); SARS-CoV-2 reverse transcription polymerase chain reaction (RT-PCR) test	Schools	Unclear but online videos for children SS oral fluid were referenced	Weekly nose & throat swabs for a min. 4 weeks, one oral fluid SS at the end of term	UKHSA received electronic notifications of confirmed COVID-19 cases	N/R
Atchison 2021 (3), UK, MM, RTI (SARS-CoV-2)	ST: Blood sample from a finger prick; 2 different SARS-CoV-2 antibodies lateral flow immunoassays (LFIA) self-tests:<br>- LFIA1 - Guangzhou Wondfo Biotech Co Ltd)	Home	Instruction booklet that contained a link to an instructional video online	Once	For LFIA1, there were 3 possible test outcomes (negative, positive, invalid), while LFIA2 had 5 possible test outcomes (negative, IgG positive, IgM positive, IgG and IgM positive, invalid)	Participants asked to upload a photograph of test result which was reviewed by a clinician blinded to participant's

	was a cassette-based system containing a “control” indicator line and a “test” indicator line (for detection of combined IgM and IgG antibodies<br>- LFIA2 - Fortress Orient Gene Biotech Co Ltd) was a cassette-based system containing a “control” indicator line and separate indicator lines for IgM and IgG				10-15 minutes	interpretation; advised to continue to follow the current government advice, irrespective of test result
Bauld 2023 (4), UK, MM, RTI (SARS-CoV-2)	SS: Saliva-based SARS-CoV-2 PCR test	Testing centre on university campus	N/R	Twice per week	Results were available within 24 hours by logging into a secure online portal	Those testing positive were encouraged to complete a PCR test from the NHS and self-isolate
Blake 2020 (5), UK, MM, RTI (SARS-CoV-2)	SS for SARS-CoV-2:<br>- PCR test using swabs and saliva,<br>- Antibody tests using finger-prick dried blood sample	University campus	Participants deposited samples at dedicated collection points on campus	12 PCR tests offered weekly (10 swab, 2 saliva); 6 antibody tests offered alternate weeks	Negative test results were emailed; those with positive tests were personally advised of their result by a clinical virologist, and a central university team was notified so that the student could be safely cared for. Public Health England and a University Central Team were also notified	N/R
Brown 2019 (6), UK, MM, STI (Chlamydia)	SS: Throat and rectal swab for Chlamydia	Sexual health centre	N/R	Once	N/R	N/R

Davies 2021 (7),<br>UK,<br>Survey,<br>RTI (SARS-CoV-2)	ST: SARS-CoV-2 LFIA self-test using a nasopharyngeal swab<br>SS: Nasopharyngeal swab & self-collected saliva (2 mL, using Oragene kit [DNA Genotek, Ottawa, Ontario, Canada] and drool technique) analysed by RT-PCR (Also nurse-collected finger-prick blood analysed for antibodies)	Clinic (not specified) & outside in a sheltered area or private vehicle	Written and visual instruction provided; no direct instruction or supervision was provided from staff at the clinic facility;<br>Participants placed the sample in the 'drop box' at reception	Once	ST: 10-15 minutes for the LFIA self-test results<br><br>SS: Participants were contacted (e-mail or phone) with the results within 3 days	If positive, participants were provided with the latest government instructions on self-isolation and household isolation
Denford 2021 (8),<br>UK,<br>Qualitative,<br>RTI (SARS-CoV-2)	ST: Lateral flow device, antigen Covid-19 test (unclear sampling type)	Home	Instructions N/R.<br>Unsupervised	Daily for up to 6 days	N/R	If each test was negative people could continue to leave their home within local guidelines.
German 2023 (9) & Nikolaou 2023 (10),<br>UK,<br>MM,<br>RTI (Respiratory microorganisms)	SS:<br>- Saliva (either spitting into a tube or chewing on specialised swab),<br>- nasal lining swab (SAM, NasosorptionTM FX-i, Mucosal Diagnostics),<br>- hand swab (wet wipe, placed in home freezer and collected)	Home	Trained how to perform each SS type by member of the research team, also provided with laminated step-by-step written and photographic instructions for sampling;<br>Unsupervised;<br>Participants stored samples in home freezer until collected and sent to laboratory	Every two weeks for 6 months	Laboratory testing	N/R
Gillam 2021 (11),<br>UK,<br>MM,<br>RTI (SARS-CoV-2)	SS: Nasal swab (Copan Liquid Amies Elution Swabs (Eswabs)) for SARS-CoV-2 PCR test	University site and home	Unsupervised;<br>Participants returned the swabs in pre-booked return slots	Pre-trial: 2 tests;<br>Trial: 4 tests over 2 weeks	Laboratory testing;<br>Negative or inconclusive results were posted on participants' online accounts; positive results were communicated to participants	Participants with positive results were encouraged to inform their GP

					and NHS track and Trace by telephone	
Hirst 2021 (12) & Wanat 2021 (13), UK, MM, RTI (SARS-CoV-2)	ST: Nasal & tonsil swab for lateral flow tests (LFT); the Innova Rapid SARS-CoV-2 Antigen Test Kit (Innova Medical Group)	University site and home	Electronic training materials and instructions were available; Trainers supervised participants' first test during face-to-face or online training	Weekly	Participants interpreted results as negative, positive, or inconclusive (failed test); 30 minutes	Participants were asked to upload a photograph of their result and upload their health status daily to track any COVID19 related symptoms If COVID-19 symptoms developed or a positive result, participants were instructed to self-isolate and book an RT-PCR test
Jing 2021 (14), UK, Survey, RTI (SARS-CoV-2)	ST: Finger-prick blood sample for SARS-CoV-2 IgG antibodies AbC-19 Rapid Test (Abingdon Health)	In-car setting at university car park	Written instructions and YouTube video prior to testing; ST was overseen by the researchers	Once	Viewing window with the control line (C-line) and test line (T-Line); 20 minutes	N/R
Jing 2022 (15), UK, Survey, RTI (SARS-CoV-2)	ST: Finger-prick blood sample for SARS-CoV-2 IgG antibodies AbC-19 Rapid Test (Abingdon Health)	Home	Written instructions and YouTube video prior to testing; Unsupervised	Once	Participants interpreted simulated test results and choose the reading that most closely matched their interpretation from the four options provided: positive, negative, failed/invalid and unsure	Upload photograph of their completed test and a photograph of the simulated result
Jones 2021 (16), UK, Qualitative, RTI (SARS-CoV-2)	SS: SARS-CoV-2 LFT (unclear sampling type)	Testing site	N/R	2 LFTs to be taken 7 days apart	N/R	N/R

Lown 2023 (17),<br>UK,<br>MM,<br>RTI (sore throat)	SS: A test kit included 2 throat swabs (bacterial and viral) and 2 saliva collection kits (saliva pot and saliva sponge); rapid antigen detection tests	Home	A web-based tool was available to help with self-assessment of sore throat and taking throat swabs and saliva samples, including a video for the swab tests.<br><br>Supervised: Participants had a video call during which they were observed (without intervention) performing the swab and saliva tests.	Once with each SS method	N/R	N/R
Martin 2021 (18),<br>UK,<br>Survey<br>RTI (SARS-CoV-2)	SS: unclear sampling type for SARS-CoV-2	Home	Unsupervised	Daily for 6 days	Laboratory testing	Participants asked to upload a photograph of a completed test
Powell 2016 (19),<br>UK,<br>Qualitative,<br>STI (Chlamydia)	SS: urine sample or vulvo-vaginal swabs	Usually at home, some in health clinics	N/R	N/R	Results usually communicated by text or email	N/R
Seguin 2018 (20),<br>UK,<br>MM,<br>STI (HIV)	SS: finger-prick blood sample; HIV antibodies test; TINY collection device in conjunction with the ROCHE HIV Combi assay (Roche Diagnostics GmbH, Mannheim, Germany)	Home	Instruction sheet with information on how to collect the sample; a link to a video demonstration;<br>Participants asked to post the sample to the laboratory, received information on labelling and postage, how the result would be communicated and why participants may be contacted;<br>Distributors briefly explained the processes involved with kit use;	Once	3 possible test results: (1) HIV not detected, (2) reactive sample or (3) insufficient sample.<br><br>Anonymised results communicated to the researcher from the laboratory on the day of analysis; researchers linked results with participants and contacted them (by text message or telephone for reactive results)	Duty of care for participants described but unclear what exactly participants were supposed to do after the test

			Unsupervised			
UKHSA 2022 (21),<br>UK,<br>MM,<br>RTI (SARS-CoV-2)	ST: Nasal swab with pre-filled sample extraction tubes (test kit widely available for home ST at the time)	Home	Instructions provided to blind and partially sighted (BPS) people via a live video with assistance. Supervised with assistance provided by Be My Eyes agents.	Once	15 minutes for the result	N/R
UKHSA 2022 (22),<br>UK,<br>MM,<br>RTI (SARS-CoV-2)	SS: PCR self-swab<br>(details N/R)	Home	Instructions provided to BPS people. Initially a 24-page printed instruction booklet, later changed to 12-page printed booklet also available in alternative formats.<br>Supervised: Users were observed by video but without intervening.	Once	N/R	N/R
UKHSA 2023 (23),<br>UK,<br>MM,<br>RTI (SARS-CoV-2)	N/R	Home	Unsupervised	Varied: everyone able to access free, twice-a-week LFD testing; contacts of cases with COVID-19 could test daily rather than self-isolate.	N/R	N/R
UKHSA 2023 (24),<br>UK,<br>MM,<br>RTI (SARS-CoV-2)	N/R – presumed ST<br>Direct LAMP (SEND and prison settings);	(1) special educational needs and disabilities (SEND)	N/R<br>(1) SEND - supervised by parent/guardian	(1) SEND: one per week (pilot 13 weeks);	N/R	N/R

	RT-PCR (adult social care setting – not eligible for review)	settings; (2) prison		(2) Prison: staff twice weekly		
UKHSA 2023 (25), UK, Survey, RTI (SARS-CoV-2)	N/R – presumed SS; oropharyngeal or nasal swab	Regional testing site	Verbal and written instructions to collect the sample, and instructions booklet on how to perform the PCR test sample collection. Standard written national instructions were provided. Unsupervised	Once	N/R	N/R
UKHSA 2024 (26), UK, Survey, RTI (SARS-CoV-2)	ST for SARS-CoV-2: Higher Education Institution (HEI) - Innova LFDs; Schools & colleges - n/r	(1): HEI test sites; (2) schools & colleges: test sites within educational setting for 2 weeks, then home testing	Instructions N/R; Unsupervised	(1) HEI: twice, 3-4 days apart; (2) schools & colleges: N/R but evaluation covers 5-week period.	N/R	(1) HEI: those testing positive advised to seek a confirmatory PCR, and required to self-isolate for 10 days. (2) Schools & colleges: Positive test results identified at an ATS did not initially require a confirmatory PCR but this was reinstated from 31 March 2021. However, positive LFD tests taken at home did require a confirmatory PCR.
Witzel 2019 (27), Witzel 2020 (28), Witzel 2020 (29),	ST: finger-prick blood sample for HIV antibodies ST; BioSURE^{TM} HIV ST kit (an	Home	Written information provided with the test kit, an online video providing	Trial arm 1 = once;	(From protocol): When the test is completed, two lines can appear on the paper test	N/R

Witzel 2021 (30), Nicholls 2022 (31), UK, MM+qualitative, STI (HIV)	antibody immunoassay detecting HIV 1/2 antibodies from approximately 28 days after infection)		instructions on kit use (produced by BioSURE) was available on the study website; Unsupervised ST	Trial arm 2 = every 3 months for 2 years	strip; the upper (control) line and the lower (test line) becomes visible if the applied sample contains sufficient antibodies to HIV; 15 minutes	

Part B – Other European studies

STUDIES IN OTHER EUROPEAN COUNTRIES						
**Author, year (reference), country, study design, infection**	**Details of the self-sampling (SS) & self-testing (ST)**	**Setting**	**User instructions, training & supervision**	**SS/ST number & frequency**	**Details related to SS/ST results**	**User instructions & actions after SS/ST**
Agusti 2022 (32), Spain, Survey, RTI (SARS-CoV-2)	ST: Antigen-detecting rapid diagnostic tests (Ag-RDTs) (PanBIO ™ COVID-19 Antigen Self-Test, Abbot Laboratories, Chicago, US)	Home	ST kits included a pictorial leaflet with guidance on how to perform ST; an instructional video was available on YouTube. N/R but assume unsupervised if at home	N/R but data collected after ST	N/R	Participants asked to send ST result and its picture which was assessed by a coordinator & a microbiologist. All participants with a positive result were contacted and were recommended to self-isolate and contact their doctor.
Baetselier 2019 (33), Belgium, Survey, STIs	SS: First-void urine using Colli-Pee device (Novosanis, Belgium); Laboratory testing for chlamydia trachomatis (CT), neisseria gonorrhoeae (NG), Mycoplasma genitalium	Home	User instructions provided with the device; participants were asked to send the urine sample to the laboratory by regular post. N/R but assume unsupervised if at home	1-3 (data reported after the first use)	The result was not disclosed to the physician or participant.	N/R

	(MG) and Trichomonas vaginalis (TV)					
Camus 2021 (34),<br>France,<br>Survey,<br>Genital infections	SS: Vaginal self-swab using Copan ESwab1 (Biome´rieux, Marcy l'Etoile, France) and one using Cobas PCR media™ (Roche Diagnostics, Meylan, France). Laboratory testing	N/R but inferred that SS was done at the clinics	Written and schematic instructions were provided to patients.<br>N/R if supervised	Once with each swab	N/R	N/R
Castell 2014 (35),<br>Germany,<br>Survey,<br>STIs (range of microorganisms)	SS: Cervico-vaginal lavage; 2nd generation Delphi Screener™ (Delphi Bioscience, BV Scherpenzeel, The Netherlands);<br><br>Sexually Transmitted Infection Profiling performed at a laboratory (multiplex PCR, xMAP technology, Luminex®)	Home	Unclear what instructions were provided to participants but article describes the SS process;<br>After SS, participants transferred the lavage to the transportation tube and mailed it to the laboratory;<br>Unsupervised SS	Once	Sexually transmitted infection profiling was performed on the self-collected sample at a research facility;<br>Those testing positive were contacted	Those testing positive for gonorrhoea were advised to immediately contact a gynaecologist for confirmation by clinical diagnosis; if analysis resulted in any other suspicious findings, participants received a recommendation to see their gynaecologist
Colom-Cadena 2022 (36),<br>Spain,<br>Survey,<br>RTI (SARS-CoV-2)	ST: Nasal swab and Panbio™ SARS-CoV-2 Ag Rapid Diagnostic Tests (Abbot Laboratories, Chicago, US)	Schools & summer camps	ST procedure explained via paper-based schematic and illustrated instructions and video;<br>Supervised (in School A): previously trained older pupils supervised the sampling procedure	School A: weekly ST for 8 weeks;<br>School B & summer camp: once	In School A: older trained pupils read the results;<br>In School B and summer camps: participants read the results themselves and the healthcare team validated them	Positive cases were referred to the health centre for a RT-PCR to confirm the results
Fajardo 2022 (37),	ST: Oral fluid sample for hepatitis C (HCV) antibodies	Community sites	Instructions were abbreviated into double-	Once	In findings authors mention 'control line' and 'test line';	Healthcare professional read

Georgia,<br>MM,<br>Hepatitis C	ST with OraQuick® HCV Rapid Antibody Test Kit (OraSure, USA)		sided page with pictorial instructions and simple nontechnical language;<br>Supervised: ST was observed by healthcare professional who completed standardised product-specific checklist of errors and difficulties observed during test procedures; help was provided if requested by participant and if they had exhausted efforts for ST independently		20 minutes	the result (inter-reader agreement) and then performed and interpreted a second test (inter-operator agreement)
Flipse 2022 (38),<br>Netherlands,<br>Survey,<br>RTI (range of respiratory viruses)	SS: self-collected gargle fluids and self-swabbing oropharynx or nasopharynx;	N/R	N/R	Once (inference)	Laboratory testing: molecular diagnostics of respiratory viruses	N/R
Grandahl 2020 (39) &<br>Grandahl 2020 (40),<br>Sweden,<br>Survey &<br>Qualitative,<br>STIs (Chlamydia and gonorrhoea)	SS: Urine (men), vaginal SS (women)	Home	Participants returned samples using prepaid envelope;<br>Unsupervised	Once	(From Appendix 2) Individual test results were provided on eHealth website in personal records	(From Appendix 2) Persons with a detected infection are asked to visit a public healthcare clinic for free treatment and mandatory partner notification
Haussig 2019 (41),<br>Germany,<br>Survey,<br>RTIs (range of respiratory pathogens)	SS: nasal swabs; multiplex PCR tests RespiFinder® 2SMART (Pathofinder, Maastricht, Netherlands) for 22 viral and bacterial pathogens	N/R	Information leaflet explaining the procedures, an instruction on how to take an anterior nasal swab;<br>Users required to send samples to laboratory for testing;	3 swabs per adults; 4 swabs per child	Laboratory results were fed back to each participant through their personal GrippeWeb diary (webpage with participant login); because the laboratory analysis included notifiable	N/R

			Unsupervised		diseases, the responsible local public health department was notified whenever one of these pathogens was identified	
Hoehl 2021 (42),<br>Germany,<br>Survey,<br>RTI (SARS-CoV-2)	ST: anterior nasal swab for SARS-CoV-2 rapid antigen test (R-Biopharm, Darmstadt)	Home	Video and written instructions provided on how to carry out the test, and a study hotline was available for queries;<br>Unsupervised	Every 48-hours for 7 weeks	N/R	Samples on which the teachers reported positive or unclear results were investigated using RT-PCR at the Institute of Medical Virology, Frankfurt University Hospital
Iruzubieta 2021 (43),<br>Spain,<br>Survey,<br>RTI (SARS-CoV-2)	ST: finger-prick blood sample, Lungene COVID-19 IgG/IgM Rapid Test Cassette (Hangzhou Clongene Biotech Co., Ltd., China)	Home	Paper-based and video-based instructions;<br>Unsupervised	Once	15 minutes	Participants uploaded photo of test result which was checked by a health professional
Lafort 2023 (44),<br>Belgium,<br>Survey,<br>RTI (SARS-CoV-2)	ST: rapid antigen tests for SARS-CoV-2, usually using nasal samples	Home	Unsupervised	N/R	N/R	N/R
Leenen 2020 (45),<br>Netherlands,<br>Survey,<br>STIs (range of STIs)	SS:<br>- oral swab for chlamydia (CT) and gonorrhoea (NG)<br>- anorectal swab for CT and NG<br>- self-collected urine for genital CT and NG and for syphilis and hepatitis B (HBV),<br>- finger-prick blood sampling;<br>Swabs and urine were processed with a PCR for CT	Home	Instructions on home sampling procedures and on how to return the samples;<br>Participants returned SS to laboratory;<br>Unsupervised	Once	The STI clinic communicated the laboratory test results to the participants via routine STI clinic protocol (negative = text message; positive = phone call)	Participants were invited to clinic as required (e.g., for treatment, counselling, partner testing etc.)

	and NG (Roche Cobas 4800, Roche Diagnostics, Basel, Switzerland);<br>A syphilis screening test (Elecsys® syphilis immunoassay, Roche, Basel, Switzerland) was performed; or a rapid plasma reagin reditest (Biokit, Barcelona, Spain) for those who reported a history of syphilis;<br>For HBV unvaccinated, HBV serology was performed on the blood sample. In case of a positive anti-hepatitis B core antigen test, hepatitis B surface antigen (HBsAg) test and anti-HBs (HBsAg II and anti-HBs II, Roche, Basel, Switzerland) were performed to determine HBV status					
Lindner 2021 (46),<br>Germany,<br>Survey,<br>RTI (SARS-CoV-2)	SS: nasal swabs; STANDARD Q COVID-19 Ag Test (SD Biosensor, Korea)	Ambulatory testing facility	Written and illustrated instructions in German or English, adapted from the manufacturer's instructions for use;<br>Procedures observed by a study physician, without answering questions or providing corrections (Supervised)	Once	N/R	N/R
Loo 2017 (47),<br>Netherlands,<br>Survey,	SS: self-collected dried blood spot (DBS) for	Clinic	Instruction scheme with several steps depicted by icons and a clear picture of	Once	Laboratory testing	N/R

STI (HIV, hepatitis B, syphillis)	laboratory testing for HIV, hepatitis B and syphilis (First 50% of participants completed SS, the second 50% had provider-collected sample and these were compared)		the DBS card with the 5 circles (cross section 1.5 cm) for sampling;<br>Unsupervised			
Loos 2016 (48),<br>Belgium,<br>MM;<br>STI (HIV)	SS: oral fluid; Oracol device (Malvern Medical Developments, Worcester, UK)	Community sites	During community sessions, a medical doctor introduced the testing and a community member with HIV offered a testimony;<br>Detailed explanation of procedures was provided individually in a separate area	Once	Laboratory testing;<br>Participants chose to collect results from local HIV/STI testing facility or via a secure website;<br>Participants received an email notifying them their results were ready to be collected/viewed;<br>If participants tested positive, their results instead were communicated over the phone and confirmatory blood testing at clinic recommended;<br>Results available approx. 1 week after ST	Those testing positive were recommended confirmatory blood testing at a clinic
Marinos 2022 (49),<br>Greece,<br>Survey,<br>RTI (SARS-CoV-2)	ST: (From the Introduction) rapid antigen detection tests (RADTs) for COVID-19 (Participants were asked generally about ST for SARS-CoV-2, not specific test types)	N/R	N/R	N/R	N/R	Positive results of ST had to be confirmed by another RADT or PCR test by an authorised provider
Møller 2022 (50),<br>Denmark,<br>Survey,<br>RTI (SARS-CoV-2)	ST: different RADTs using nasal swabs<br>- COVID-19 Antigen Detection Kit (DNA	Home	Illustrated instruction pamphlet and a link to an online instruction video;<br>Unsupervised	Once	For the test to be conclusive, a line should appear after 10 minutes in the control area of the test plate. If a line appeared in the test area, the	Participants a photograph of the test plates by email to the project inbox;

	Diagnostic A/S, Risskov, Denmark)<br>- SARS-CoV-2 Antigen Rapid Test (Hangzhou Immuno Biotech Co Ltd, Hangzhou, China)<br>- a nasopharyngeal RADT - COVID-19 Antigen Rapid Test Device (Abbott Rapid Diagnostic Jena GmbH, Jena, Germany)<br>(Compared to oropharyngeal swab and PCR test performed by a trained health care worker)				test was positive, and no line indicated a negative result. Weak lines in the test area were also considered positive results;<br>10 minutes<br>Results were self-interpreted by participants, and the interpretation was confirmed by the researchers based on test photographs	in case of a positive or inconclusive self-RADT result, participants were advised to call in for further instruction
Nash 2021 (51),<br>Italy,<br>Survey<br>STI (HIV)	ST for HIV (produced by Mylan)	N/R	Supervised;<br>Unclear whether healthcare professional provided assistance	Once	N/R	N/R
Prazuck 2016 (52),<br>France,<br>Survey,<br>STI (HIV)	ST: finger-stick whole-blood HIV test ("autotest VIH1," AAZ-LMB, Rungis, France)	Clinic	Written instructions provided;<br>Supervised: participant could choose whether to perform test alone or with observer; the observer confirmed the different steps that were followed by the participant on a standardized sheet;	Once	Participants did not read the results of their own test; they interpreted standardised tests	Participant moved to the next room with a trained staff member who performed the regular HIV rapid test, including pre- and post-counselling
Prazuck 2021 (53),<br>France,<br>Survey,<br>RTI (SARS-CoV-2)	ST: blood sample for SARS-CoV-2 antibodies COVID-PRESTO*R self-test	N/R	Written instructions and an instructional video; participants invited to use either one or both;<br>Supervised: could assist the user, if asked to, and	Once	Uses anti-human immunoglobulin (Ig) M antibody (test line M), anti-human IgG antibody (test line G) and rabbit IgG (control line C) immobilized on a nitrocellulose strip	N/R

			evaluate the execution of the different tasks		10 minutes	
Prazuck 2022 (54), France, Survey, RTI (SARS-CoV-2)	ST: Nasal sponge self-sampling; A vertical flow test using highly sensitive monoclonal antibodies to detect SARS-CoV-2 core antigen in a nasal sample - COVID-VIRO ALL IN® (AAZ-LMB, Boulogne-Billancourt)	Hospital COVID-19 testing units	Written instructions were provided; Supervised (Observers completed evaluation forms)	Once	Coloured test line appears in the results window only if SARS-CoV-2 antigens are present in the sample. The intensity of the coloured test line vary depending on the amount of SARS-CoV-2 antigens present in the sample. In the event of no SARS-CoV-2 antigen being present, the test line will not display any colouration. 15 minutes for results	N/R
Prinsenberg 2022 (55), Netherlands, Survey, Hepatitis C	SS: Finger-prick dried blood spot for hepatitis C virus (HCV) RNA	Home	Paper illustrations and an online instructional video on how to collect finger-prick blood samples; Unsupervised; Participants returned samples to the laboratory	Once	Laboratory testing; Participants receive their test result anonymously online	Users who test positive are linked to care to confirm their test result and start treatment
Rohr 2022 (56), Germany, Qualitative, RTI (SARS-CoV-2)	SS: Gargle for SARS-CoV-2 testing	Home	Written instructions and website link to further multilingual explanations (in German, English, Russian, Italian, Turkish) and a video showing the SS procedure; Unsupervised	Once	Laboratory testing; Results reported online	N/R
Schuit 2022 (57), Netherlands, Survey, RTI (SARS-CoV-2)	ST: Nasal and oropharyngeal-nasal swabs; testing for SARS-CoV-2 antigens. 3 tests: Flowflex (Acon Laboratories), MPBio (MP	Home	Manufacturers' instructions were provided to perform nasal SS; investigators' instructions were provided for oropharyngeal SS together with	Once	Participants interpreted their test results visually according to manufacturer's instructions. 15-30 minutes for results.	

	Biomedicals), and Clinitest (Siemens-Healthineers)		manufacturers' instructions for nasal SS.<br>Unsupervised			
Schuit 2022 (58),<br>Netherlands,<br>Survey,<br>RTI (SARS-CoV-2)	ST: Self-performed nasal swab and saliva (oral fluid) spitting test.<br>COVID-19 Antigen Rapid Test (Ag-RDT).<br>Saliva ST - Hangzhou AllTest Biotech Co. Ltd.<br>Nasal Ag-RDT - SD Biosensor SARS-CoV-2 Rapid Antigen Test Nasal for self-testing by Roche Diagnostics.	Home	Instructions provided by the manufacturers.<br>Unsupervised.	Once using each SS method	Participants interpreted their Ag-RDT test results visually in accordance with the instructions.	
Sweeney-Reed 2021 (59),<br>Germany,<br>Survey,<br>RTI (SARS-CoV-2)	SS: Gargle for SARS-CoV-2 testing;<br>Gargling of 10 ml of 0.9% NaCl-solution for 5–10 s was performed	Home	Detailed, illustrated instruction leaflet provided;<br>Unsupervised	Twice a week	Laboratory testing;<br>Results were available within 48 hours via an app	N/R
Tonen-Wolyec 2020 (60),<br>France,<br>Survey,<br>RTI (SARS-CoV-2)	ST: blood sampling for SARS-CoV-2 antibodies ST;<br>Exacto® COVID-19 self-test (Biosynex Swiss SA, Freiburg, Switzerland)	Home	Written and video instructions;<br>Supervised: observer used standardised sheet to record participants' progress through ST steps; verbal assistance was provided	Once	Result was interpreted as from a number of different lines possible (control, IgG, IgM).<br>10-15 minutes	Participants with positive ST results were referred to a clinically certified laboratory for result confirmation
Tonen-Wolyec 2021 (61),<br>France,<br>Survey,<br>RTI (SARS-CoV-2)	ST: Nasal mid-turbinate (NMT) sampling (swab inserted about 2–3 cm into nostril parallel to the palate until resistance is met at turbinate); Ag-RDST BIOSYNEX Antigen Self-Test COVID-19 Ag+ (Biosynex Swiss SA, Freiburg, Switzerland) for COVID-19	Private setting (unclear what kind)	Manufacturers' instructions: a leaflet in French, English, German, Dutch, and Italian.<br>A video with instructions was available online from YouTube in French and English.<br><br>Supervised: A trained observer observed the ST	Once	A red line appears in the test line area: the result is negative when no red line appears in the test line area; an internal control is assured by a red line appearing in the control line area.<br>10-15 minutes	N/R

	antigenic testing from self-collected NMT secretions		and recorded performance of each step and appeals for assistance – but unclear if they offered assistance.			
Wachinger 2021 (62),<br>Germany,<br>Qualitative,<br>RTI (SARS-CoV-2)	ST: Anterior-nasal swabs, STANDARD Q COVID-19 Ag Tests (SD Biosensor, Gyeonggi-do, Korea)	Home	Step-by-step guide on how to perform ST, plus a telephone and email hotlines that could be contacted in case of screening-related questions	Three times per week for 9 weeks	N/R	N/R
Weidlich 2023 (63),<br>Germany,<br>Survey,<br>STIs (Chlamydia, gonorrhoea)	SS: Oropharyngeal and anal swabs, Copan FLOQSwab 552C®* (Copan, Brescia, Italy);<br>Self-collected urine, Copan Self-UriSponge systemTM 8E031S100* (Copan, Brescia, Italy)<br><br>(SS compared to professionally-collected samples)	Clinic	Instructions on the proper use of swabs through pictograms accompanied by explanatory text	Once	Real-time PCR for CT/NG was performed immediately using the same Cepheid Xpert CT/NG assay and software Dx system version 4.8 (Cepheid AB, Solna, Sweden) for all samples	N/R
Würstle 2021 (64),<br>Germany,<br>Survey,<br>RTI (SARS-CoV-2)	SS: Oropharyngeal swab for SARS-CoV-2 PCR testing, collected using FLOQswabs 552C 80 mm (Copan, Brescia, Italy, no transport media); Optionally one anorectal self-swab using UTM 305C swabs (Copan, Brescia, Italy). (SS compared to professionally-collected samples)	Hospital	Two separate instructions (for each swab type) with no further assistance;<br>Unsupervised	Once	Laboratory testing;<br>One self-collected swab was sent for diagnostic analysis immediately following sampling; the other was stored at ambient temperature for 48 h before being analysed	N/R

Supplementary Table S2: Summary of quantitative findings from peer-reviewed studies conducted in Europe

Summary of key quantitative findings*

- Overall acceptability: 71.4-100%
- Instructions easy to understand: 82-100%
- SS/ST easy to perform: 42.3⁺-98.5%
- Some level of pain/discomfort/unpleasant‡ collecting sample: 16.7-52%
- Ease/confidence interpreting results: 88.5-100%
- Belief in test accuracy/reliability: 68.1-96.4%
- Willingness to perform SS/ST again in the future: 71.4% - 99%

*Across sampling and infection types

⁺Respondents were mostly heterosexual men who were first-time users of HIVST

‡No pain/discomfort/unpleasantness was most frequently endorsed response

**Questionnaire item** (number of reports)	**Summary of quantitative findings** (with reference numbers)
**Overall satisfaction/ acceptability** (n=16)	**Blood:** 94.3% of respondents who performed blood-based ST for COVID-19⁺ (53) and between 71.4-100% of respondents who performed blood-based ST for HIV* (27,30,51) reported a good overall experience. Additionally, 96.9% of respondents who performed blood-based SS for HIV (1) agreed SS was acceptable.<br><br>**Cervicovaginal/urine:** between 93.0-98.1% of respondents who performed cervicovaginal (women) or urine (men) SS for STI†s (35,40) reported a good overall experience.<br><br>**Nasopharyngeal swab:** majority (91.1-100%) of respondents who performed SS for COVID-19 (11,36) or RTI‡ (41) reported a good overall experience. Additionally, majority (90.6-91.4%) of respondents who performed ST for COVID-19 (32,61) reported a good overall experience.<br><br>**Saliva:** 98.6% or respondents who performed SS for COVID-19 (4) rated their experience as ‘excellent’ or ‘good’.

	**Sampling type comparisons:** One study (10) compared different SS types (nasal lining fluid, saliva and hand swabs) for RTI. Adults reported high acceptability (98-100%) across all SS types; children of all ages reported high levels (100%) of acceptability of hand swabs, acceptability of saliva varied across ages from 70% (toddlers) to 100% (school aged), nasal lining fluid acceptability decreased with age (15% infants).<br><br>**Sampling type not stratified[1]:** One study (5) included multiple SS types (swab, saliva, blood) for COVID-19 but reported high satisfaction (89.9%) across SS types.<br><br>**Throat swab:** 92.1% of respondents who performed a throat swab ST for COVID-19 reported good overall acceptability (12).
**Ease of understanding of SS/ST instructions** (n=21)	**Blood:** majority of respondents (88.5-98.1%) who performed ST for COVID-19 (3,14,15,43,53,60) and HIV (27,30,52) found instructions clear to use; majority of respondents (82-95%) who performed SS for hepatitis C virus (HCV) (55) and HIV (1,20,47) found instructions clear to use.<br><br>**Nasopharyngeal swab:** over 90% of respondents who performed SS for COVID-19 (11), 83.8-94.3% of respondents who performed ST for COVID-19 (57,61), and 100% of respondents who performed SS for RTI (41) found instructions clear to use.<br><br>**Nasal and oropharyngeal swab (combined):** 88.1-95% of respondents who performed ST for COVID-19 (58) found the instructions clear to use.<br><br>**Nasal sponge:** 98.5% of adults, 83.3% of children, and 81.3% of teenage respondents who performed ST for COVID-19 (54) found the instructions clear to use.<br><br>**Sampling type not stratified[3]:** 95.7% of respondents who performed SS for STI using multiple sampling methods found instructions clear to use (45).<br><br>**Throat swab and saliva sample:** majority (% not extractable) of respondents who performed SS for sore throat found instructions easy to follow (17).
**Ease of performing SS/ST** (n=26)	**Blood:** between 70-95.2% of respondents who performed ST for COVID-19 (3,14,15,43,60) and between 42.3-97% of respondents who performed ST for HIV (27,30,51,52) found it very/fairly easy to perform; 50% of respondents who performed SS for HCV (55) and 60.7-86.3% who performed SS for HIV (1,20,47) found it very/fairly easy to perform.<br><br>**Cervicovaginal:** 88.9% respondents who performed SS for STI found it was easy to perform (35).<br><br>**Gargle fluid compared with nasopharyngeal swab:** median score higher for gargle fluid than nasopharyngeal swab among participants SS for RTI (38).

	**Nasopharyngeal swab:** majority (approx. 70.0-87.8%) of respondents who performed SS for COVID-19 (11,18,36,46) found it easy to perform. Majority of respondents who performed ST for COVID-19 (32,42) reported having no difficulties performing the test. Additionally, respondents who performed ST for COVID-19 (61) reported it was easy to collect sample in both nostrils (90.6%) and deposit sample into the well (91.4%).<br><br>**Nasal sponge:** 98.5% of adults and 92.5% of child and teenage respondents who performed ST for COVID-19 found it easy to perform (54).<br><br>**Oral fluid:** between 80-89% respondents who performed ST for HCV found it easy to perform (37).<br><br>**Oropharyngeal swab:** 88.1% of respondents who performed SS for STI found it easy to perform (63).<br><br>**Rectal:** 75.2% of respondents who performed SS for STI found it easy to perform (63).<br><br>**Urine:** 89.1- 87.8% of respondents who performed SS for STI found it easy to perform (33,63).
**Ease of / confidence in interpreting results** (n=11)	**Blood:** of respondents who performed ST for COVID-19, 94.4-98.3% reported the results were easy to interpret (14,15,53,60) and 99.3% were confident they had interpreted the results correctly (3). Of respondents who performed ST for HIV (52), 96.6% reported it was easy to interpret positive test results compared with invalid tests (93.9%).<br><br>**Nasopharyngeal swab:** 88.5-92.4% of respondents who performed SS for COVID-19 were confident they had interpreted the results correctly (7,36). Additionally, 98-100% of respondents who performed ST for COVID-19 (58,61) were confident they had interpreted the results correctly.<br><br>**Nasal and oropharyngeal swab (combined):** 96.7-97.1% of respondents who performed ST for COVID-19 were confident they had interpreted the results correctly (57,58).<br><br>**Nasal sponge:** 98.5% of adult respondents who performed ST for COVID-19 found results the results easy to interpret (54).<br><br>**Throat swab:** 100% of respondents who performed ST for COVID-19 were confident they had interpreted the results correctly (12).
**Pain, discomfort and/or fear related to SS/ST** (n=6)	**Blood:** between 26.3 and 45.6% of respondents who performed SS for HIV reported it was unpleasant to collect the sample (1,47).<br><br>**Cervicovaginal:** 16.7% of respondents who performed SS for STI reported feeling partly/uncomfortable collecting the sample (35).<br><br>**Nasopharyngeal swab:** among adults who performed SS for RTI (41), most (82.0%) reported the experience was 'unproblematic' and 18% characterised the experience as 'only briefly unpleasant', for children 39% reported the experience was 'unproblematic' and 52.0% as 'only briefly unpleasant'. Among students who performed ST for COVID-19 (49), 69.2% experienced fear during the procedure.

	**Sampling type not stratified[2]:** among adults who used a SS for RTI (9), most (58%) reported no discomfort and no pain (94%) across sampling events; among children, 'moderate, severe, and extreme discomfort' were reported during 33% sampling events from children <5y compared to 7% of sampling events from children >5y. No pain was reported in 70% sampling events from children >5y and 48% sampling events in children <5y.
**Challenges in performing / interpreting SS/ST** (n=8)	**Blood:** challenges reported by respondents who performed ST for COVID-19 (3) included creating a blood drop from the finger-prick site (23.2%); using pipette (17.7%); applying blood to sample well (10.6-31.3%%)<br><br>**Cervicovaginal/urine:** among respondents who performed SS for STI (40) areas of improvement included, 'should be handled faster' (9.3%) and 'clearer information' (7.6%).<br><br>**Nasopharyngeal swab:** the most frequently endorsed challenges reported by respondents who performed SS for COVID-19 were the testing procedure was unpleasant (4%) and instructions were not clear (4%) (18); you have to interpret the result yourself (17.3%) and not having the emotional and/or logistical support to read the result (6.14%) (36); feeling nervous (3%), and fever or dizziness, poor concentration, feeling cold, aversion to self-sampling, language barrier and limited fine motor skills (1% each) (46). Among respondents who performed ST for COVID-19 (49), 22.6% reported difficulties with the procedure of taking the sample.<br><br>**Oropharyngeal:** among respondents who performed SS for COVID-19 (64), 48.0% reported no challenges and 48.0% reported their gag reflex was triggered. Among respondents who performed SS for STI (63), pain (16.1%) was the most commonly endorsed challenge obtaining the sample. An additional challenge identified from free text answers was gagging (n=21).<br><br>**Rectal:** among respondents who performed SS for STI (63), pain (18.7%) and 'I did not understand instructions' (1.7%) were the most commonly endorsed challenges obtaining the sample. Additional challenges identified from the free text answers included dryness (n=10), difficulty identifying orifice (n=4), swab depth challenge (n=3), swab insertion challenge (n=3)<br><br>**Urine:** among respondents who performed SS for STI (63), 'I did not understand written instructions' (4.8%), and 'I could not collect urine in container' (2.2%) were the most commonly endorsed challenges obtaining the sample. Additional challenges identified from the free text answers included missing information on how to proceed with container (n=9) and difficulties urinating (n=4).
**Willingness to perform SS/ST again** (n=12)	**Blood:** 98.3% of adult respondents who completed ST for COVID-19 (3), 71.4% of respondents who completed ST for HIV (51), and 73.2-80.0% of respondents who completed SS for HIV (20,47) were willing to perform the SS/ST again in the future. 79.8-97.1% (increased with age) of parents who completed ST for COVID-19 (3) were willing to perform the ST on their child again in the future.<br><br>**Nasopharyngeal swab:** 78.3-97% adults who performed SS/ST for COVID-19 (36,61), SS COVID-19 (11), and SS for RTI (41) were willing to perform the SS/ST again in the future; 95% of parents who completed SS for RTI (41) were willing to perform SS again in the future.<br><br>**Oral fluid:** 91-99% of respondents who completed ST for HCV were willing to perform the test again in the future (37).

	**Sampling type not stratified[1]:** 94.9% of respondents who SS for COVID-19 were willing to perform the sample again in the future (5).<br><br>**Sampling type not stratified[4]:** 90.3% of respondents who SS for STIs were willing to perform the sample again in the future (63).<br><br>**Urine:** 91.1% of respondents who SS for STIs were willing to perform the sample again in the future (33).<br><br>**Throat swab and saliva sample:** majority (% not extractable) of respondents who performed SS for sore throat were willing to perform the sample again in the future (17).
**Preference for SS/ST** (n=2)<br><br>**& preference for home testing** (n=9)	**Blood:** 72.6-74.4% of respondents who performed ST for COVID-19 (3), 15.8% of respondents who performed SS for HIV (47) reported a preference for home testing. 93.2% of respondents who performed ST for COVID-19 (43) appreciated ST at home during a pandemic situation, 38.2% believed this kind of test should be carried out by a healthcare professional.<br><br>**Cervicovaginal:** 78.7% of respondents who performed a SS for STIs (35) preferred to collect the sample at home than at a study centre. Additionally, 31% of respondents who performed SS for STIs (34) preferred to collect the sample at home than by a healthcare professional, or else they did not mind (43%).<br><br>**Gargle fluid**: of the respondents who performed SS gargle fluid for COVID-19 (59), approximately 55% preferred sampling at school, 30% preferred sampling at home, and 12% in a medical setting.<br><br>**Nasopharyngeal swab:** 72.0-86.7% of respondents who performed SS/ST for COVID-19 (36) or ST COVID-19 (7,32) preferred to perform the SS/ST at home, with 99.5% of education professionals (32) responding workplace availability of ST.<br><br>**Oral fluid:** 73-89% of respondents who performed ST for HCV preferred to perform ST at home (37).<br><br>**Oropharyngeal:** 34% of respondents who performed SS for COVID-19 preferred SS over healthcare professional guided sampling (64).<br><br>**Sampling type not stratified[3]:** 60.9% of respondents who performed SS for HIV preferred to collect the sample at home (45).
**Preference between sample types** (n=5)	**Gargle fluid compared with nasopharyngeal swab**: 82.3% of respondents who performed SS gargle fluid and nasopharyngeal swab for RTI (38), and approximately 39.0% who performed SS for COVID-19 (59) preferred gargle fluid. Reasons gargle fluid was preferred included ease of sample collection and test validity (59).<br><br>**Nasopharyngeal compared with throat swab:** 57.2% of respondents who performed ST nasopharyngeal and throat swabs for COVID-19 (50) preferred the nasopharyngeal swab while 33% preferred the throat swab. Reasons respondents preferred the nasal swab included it was a more

	pleasant test, shorter response time, and easy to perform. Reasons respondents preferred the throat swab included the belief it provided a more valid result and throat sampling was more comfortable.<br><br>**Nasal swab compared with saliva sample:** Participants had more difficulty with taking a sample with the nasal than with the saliva rapid antigen diagnostic test (58). There was no difference in the readability of the results between the two sample types.<br><br>**Throat swabs compared with saliva sample:** saliva pot best rated in terms of how pleasant it was to use, followed by saliva sponge, then the throat swab (17).
**Motivation(s) for SS/ST**<br>(n=6)	**Blood:** motivations for SS among those who performed SS for HCV (55) included 'I want to make sure I am not infected with HCV' (61.1%); 'I was worried I might be infected with HCV' (30.0%); 'it saves time' (25.9%); 'test was cheap' (25.9%); and 'I wanted to buy a test anonymously' (18.5%).<br><br>**Cervicovaginal/urine:** motivations for SS among those who performed SS for STI (40) included 'check health status after unprotected sex' (72.9%); suspected infection' (7.8%); and 'experiencing symptoms' (7.5%)<br><br>**Gargle sample:** motivations for SS among those who performed SS for COVID-19 (59) included, 'science' (83.3%), 'infection spread' (69.5%), and 'school opening' (24.8%).<br><br>**Nasopharyngeal swab:** motivations for SS among those who performed SS for COVID-19 (18) 'included I wanted to know if I had the virus' (22%); 'to help beat the virus in my area' (20%); 'it sounded easy to do' (17%); and 'I needed to know if I was infected so I could protect vulnerable people that I live with or meet regularly' (13%).<br><br>**Saliva:** motivations for SS among those who performed SS for COVID-19 (4) included 'knowing their own Covid-19 status in the absence of symptoms' (38.4%); 'prevention passing on infection to family and friends' (31.5%); 'to contribute to scientific research on Covid-19' (18.2%); and 'to prevent passing on infection to other colleagues/students on campus' (11.3%)<br><br>**Sample type not reported:** motivations for ST among those who performed ST for COVID-19 (44) included 'symptoms' (31.4-34%); 'risk contact' (26.9%); 'regular self-testing' (26.5%); and 'before visiting someone' (21.3%).
**Benefits of SS/ST**<br>(n=8)	**Blood:** the main benefits of ST for respondents who performed ST for HIV (51) were rapid result (68%); no need for medical prescription (36%); and privacy (21%).<br><br>**Cervicovaginal:** the main benefits of SS for respondents who performed SS for STI (34) were ease of use (96%); accordance with religious beliefs and cultural norms (21%); promote monitoring of GI (40%); cheaper (6%).

	**Nasopharyngeal swab:** the main benefits of SS/ST for respondents who performed SS/ST for COVID-19 were 'results within minutes' (83.2-86.0%) (32,36); 'convenience' (85.2%) (32); 'tests improve safety and protection against Covid' (78.6-86.7%) (32,36); 'I can take control of my health with respect to Covid' (55.1-67.0%) (32,36); 'it gives me a more relaxed feeling when meeting friends' (63.5%) (36); 'contributes to the normalization of Covid tests' (41.8-57.5%) (32,36); 'the test is free of charge' (56.1-64.8%) (32,36); and 'privacy and confidentiality' (31.6%) (32).<br><br>**Saliva:** the main benefit of SS for respondents who performed SS for STIs was convenience (93.3%) (4).<br><br>**Sampling type not stratified[1]:** the main benefits of SS for respondents who performed SS for COVID-19 (5) were 'helping to keep campus safe' (97.8%); 'being able to contribute to national efforts to control virus' (93.5%); and 'being involved in Covid-19 research' (81.7%).<br><br>**Sampling type not stratified[3]:** the main benefits of SS for respondents who performed SS for STIs (45) were **'**convenient and at own time' (64.3%); testing at home (46.4%); and no transportation required (46.4%).<br><br>**Throat swab:** the main benefits of ST for respondents who performed ST for COVID-19 (12) were beneficial for them (97%), their friends and family (99.5%), people they live with (98%), and their wider community (98.5%).
**Belief in test accuracy** (n=7)	**Blood:** 96.4% of respondents who performed ST for HIV believed the test was accurate (51).<br><br>**Nasopharyngeal swab:** between 68.1-86.7% of respondents who performed SS for COVID-19 (18,36) believed the process was accurate. Additionally, majority of respondents who performed ST for COVID-19 (32) were confident (88.8%) and trusted (93.8%) the result from the ST.<br><br>**Saliva:** 92.3% of respondents who performed SS for STIs believed the process was accurate (4).<br><br>**Sampling type not stratified[1]:** 79.6% of respondents who performed SS for COVID-19 believed the process was accurate (5).<br><br>**Throat swab:** 90% of respondents who performed ST for COVID-19 believed the test was accurate (12).
**Being prepared to pay for ST** (n=3)	**Blood:** 17.9% of respondents who ST for HIV were willing to pay more than 20€ for the ST (51).<br><br>**Cervicovaginal/urine:** 36.9% of respondent who performed SS for STIs (40) were not willing to pay for the SS, 20.3% were willing to pay a maximum of SEK 50, and 26.9% were willing to pay a maximum of SEK 100.<br><br>**Urine:** 54.3% of respondents were willing to pay 10-20 Euros (33).

Supplementary Table S3: Summary of qualitative findings from peer-reviewed studies conducted in Europe

**Author, year** (reference), infection, country	**Sample**	**Enablers, benefits of SS/ST**	**Barriers to, cons of SS/ST**	**Study conclusions related to acceptability of SS/ST**
**Ahmed-Little 2016** (1)<br>HIV<br>UK	756/2447 free-text comments	• Easier<br>• Less intimidating by avoiding contact with health services / professionals<br>• Easily accessible<br>• More timely<br>• Anonymity	• Some concerns about the procedure and the quantity of lancets and plasters (but SS still preferred over venepuncture).	Home SS via kits ordered online was acceptable and convenient.
**Atchison 2021** (3)<br>Covid-19<br>UK	Interviews n=25	• Communication & instruction (e.g., simple and straightforward)	• Communication & instruction (e.g., video too long, emphasis to read instruction before performing test, clarification on interpreting results)<br>• Performance/usability (e.g., challenges obtaining blood sample and transferring to cassette)	Most people find the instructional materials to be well-designed and accessible. Nevertheless, recommendations were made to refine instructions. Some participants noted challenges obtaining a sufficient blood sample, and there were examples of using alternative objects (e.g., pins, sewing needles) to draw blood.
**Bauld 2023** (4)<br>Covid-19<br>UK	Survey n=1750;<br>Pilot survey n=300;<br>Interviews n=48	• attitudes & beliefs (e.g., SS/ST accuracy)<br>• experience & procedure (e.g., SS/ST is not painful/invasive)<br>• settings & logistics (e.g., multiple sites with no appointment needed)<br>• social utility (e.g., protecting others)<br>• performance/usability (e.g., SS/ST is considered simple)	• experience/procedure (e.g., results' waiting times)<br>• performance/usability (e.g., difficult to produce enough requested saliva; no eat/drink 30 minutes before testing)<br>• settings & logistics (e.g., privacy of booths; sites opening times)	Participants preferred saliva testing to nasal and oral swab testing. An asymptomatic SARS-CoV-2 testing programme designed specifically for university staff and students was acceptable and was positively received.

**Author, year** (reference), infection, country	**Sample**	**Enablers, benefits of SS/ST**	**Barriers to, cons of SS/ST**	**Study conclusions related to acceptability of SS/ST**
**Blake 2020** (5)<br>Covid-19<br>UK	Survey n=99;<br>Focus groups n=10 staff, n=5 student;<br>Small group interviews n=20 students;<br>Interview n=6 students	• communication & instructions (e.g., appropriate communication about testing and test results)<br>• personal relief/motivations (e.g., test provided peace of mind and wellbeing; satisfied curiosity about health status)<br>• social utility (e.g., protecting others and expectation that others would do the same)	• experience/procedure (e.g., SS/ST is discomfort performing swab, fear of needles)<br>• settings & logistics (e.g., challenges obtaining and dropping off tests/samples)	Almost all students and staff in this study would take part in an asymptomatic self-testing programme again and would recommend it to others. PCR self-testing using throat swab or saliva was highly acceptable.
**Brown 2019** (6)<br>Chlamydia<br>UK	Pre-post interviews n=20	• personal relief / motivations (e.g., avoid stigmatisation)<br>• settings & logistics (e.g., not having to visit the clinic)<br>• Perceived reliability of the results – would do the extra-genital test or do it in the clinic if it was more reliable that way.	• attitudes & concerns (e.g., concerns about correct performance; discomfort and embarrassment to perform the test; nobody to ask questions to)<br>• experience/procedure (e.g., throat swab and ano-rectal swab uncomfortable)<br>• performance/usability (e.g., ano-rectal swab difficult to find comfortable position)<br>• settings & logistics (e.g., having to return samples by post)	The extra-genital testing was well accepted by young women, who found the instructions and process to be clear. Most reported it was easier than they anticipated, and they would be willing to perform SS/ST as part of their usual care.
**Denford 2021** (8)<br>COVID-19<br>UK	N = 52 interviews, including 35 of those who agreed to daily ST	• Needing and wanting to protect oneself and those around and prevent spreading the virus<br>• Needing, and wanting, to avoid self-isolation and leave home<br>• Protecting vulnerable people.<br>• To know when to self-isolate and prevent the spread of the virus.<br>• As rapid confirmation of COVID status and additional reassurance	• Concerns about LDF test accuracy<br>• Concerns about trusting the test result if unsure whether one took the test accurately<br>• Confusion about consequences of ST (e.g. need to self-isolate)	Participants reported a range of factors that motivated them (or not motivated some) to perform daily ST.

Author, year (reference), infection, country	Sample	Enablers, benefits of SS/ST	Barriers to, cons of SS/ST	Study conclusions related to acceptability of SS/ST
		that one did not have the virus, which helped mental wellbeing		
**Fajardo 2022** (37)<br>Hepatitis C<br>Georgia	Survey n=200; Cognitive interviews n=20; In-depth interviews n=20	• performance /usability (e.g., easy to use; acceptable price)<br>• personal relief/motivations (e.g., avoid stigmatisation)<br>• settings & logistics (e.g., possibility to test in comfortable environment; increased privacy)	• attitudes & concerns (e.g., belief that blood test is more accurate)<br>• performance/usability (e.g., collecting the oral specimen; difficulty interpreting the results, including at the required waiting time)	HCV ST is usable and acceptable testing method for both men who have sex with men (MSM) and transgender (TG) people and people who inject drugs (PWID). Findings indicate that MSM/TG people who completed the HCV ST correctly and without assistance was significantly higher than PWID.
**German 2023** (9)<br>Respiratory pathogens<br>UK	Survey n=141 [n=36 families]; Interviews n=24	• communication, training & instructions (e.g., instructions become easier to follow with repetition)<br>• performance/usability (e.g., hand swab is considered simple and better than saliva and nasal)	• experience/procedure (e.g., saliva swabs considered disgusting; discomfort with nasal sampling for younger children; no eat/drink 30 minutes before testing; refrigerating/freezing requirement)<br>• performance/usability (e.g., difficult to perform saliva and nasal swab on children)	Simplicity of procedure enhanced adherence among the families participating in the study. In general, parents found sampling method easy to carry out, particularly the less invasive ones for the children.
**Grandahl 2020** (39)<br>STIs<br>Sweden	Interviews n=11 women, n=9 men	• communication, training & instructions (e.g., results received relatively quickly)<br>• performance/usability (e.g., ease use; free of charge)<br>• personal relief/motivations (e.g., ST allows avoiding clinic testing which does not suit all; relief within the couple relationship; relief after unprotected sex; anonymity in the community)<br>• settings & logistics (e.g., increased privacy; easy to access and order online; discrete packaging)	• attitudes & concerns (e.g., concerns regarding personal data; sample handled incorrectly; uncertainty about correct performance)<br>• communication, training & instructions (e.g., instructions' language not understood; lack of counselling; unclear what to do if they had infection)<br>• settings & logistics (e.g., concerns over the sample going missing in the post)	The free-of-charge SS test service was highly appreciated by the participants. Checking after unprotected sex or a regular routine – 'to be on the safe side' – was the key reason for ordering the test.

**Author, year** (reference), infection, country	**Sample**	**Enablers, benefits of SS/ST**	**Barriers to, cons of SS/ST**	**Study conclusions related to acceptability of SS/ST**
		• social utility (e.g., protect others; reduce spread of infections)		
**Hirst 2021** (12)<br>Covid-19<br>UK	Survey n=213;<br>Interviews n=18	• communication, training & instructions (e.g., training valuable)<br>• personal relief/motivations (e.g., regular testing provided reassurance on own infection)<br>• social utility (e.g., reduced fear of accidentally infecting family and friends)	• attitudes & concerns (e.g., uncertainty about ST results' accuracy; concerns about correct performance)<br>• communication, training & instructions (e.g., more information false negatives)<br>• experience / procedure (e.g., ST found uncomfortable or even 'extremely unpleasant')	ST was acceptable and results could be correctly interpreted. Results showed perception of broad benefits to testing for participants and others, despite the fact that beliefs about test accuracy varied.
**Jones 2021** (16)<br>Covid-19<br>UK	Interviews n=30 university students	• communication, training & instructions (e.g., effective awareness campaign; results received relatively quickly)<br>• personal relief/motivations (e.g., test provided peace of mind and reduced anxiety; increased confidence engaging in activities that were allowed<br>• performance/usability (e.g., ease use; free of charge)<br>• settings & logistics (e.g., easy to access the test; ST sites had good capacity and proximity; low risk of transmission at ST sites)<br>• social utility (e.g., protect others; reduce transmission)	• attitudes & concerns (e.g., rely on others being at risk in order to test; testing is stigmatising; fear of consequences if testing positive)<br>• communication, training & instructions (e.g., testing reminders; emphasise benefits of testing; address concerns in case of positive results; frame testing as a social norm; test results messages clarify residual risk of infection inherent in a negative test; emphasise need of testing after vaccination)<br>• experience/procedure (e.g., not pleasant, gagging and vomiting)<br>• settings & logistics (e.g., increase sites accessibility, drop-in or same-day booking)	The findings show that the testing programme is convenient and well organised, with testing as a potential requirement to access social events, and self-isolation support being key contributor to uptake. Participants' experiences of getting tested were mostly positive and generally intended to continue testing.
**Loos 2016** (48)<br>HIV<br>Belgium	Survey n=142;<br>Field notes n=137;<br>Informal interviews with tester n=41	• communication, training & instructions (e.g., convenient to access results online; option to collect results from the clinic)<br>• experience & procedure (e.g., painless)	• attitudes & concerns (e.g., saliva-based testing led to misconceptions regarding HIV transmission [unintended consequence)]	Acceptability of the intervention differed between individual participants and settings. Acceptance was higher among women, in churches and settings where community leaders were engaged in HIV

**Author, year** (reference), infection, country	**Sample**	**Enablers, benefits of SS/ST**	**Barriers to, cons of SS/ST**	**Study conclusions related to acceptability of SS/ST**
		• performance/usability (e.g., ease of use; free of charge; better than blood test)<br>• personal relief/motivations (e.g., provided reassurance on own infection)<br>• settings & logistics (e.g., testing without seeing physician)		awareness raising. Higher preventive outcomes were observed in settings with lower acceptance. The presence of the intervention team visualized the magnitude of the HIV epidemic to the public and promoted HIV testing uptake at large, for example, those who declined indicated they would take up testing later.
**Lown 2023** (17)<br>Sore throat<br>UK	Interviews n = 38 (including 8 mothers, 1 father)	• SS by throat swab and saliva sampling were acceptable.<br>• Self-swabbing throat preferred over swabbing by someone else as could control the swab and gag reflex.<br>• Saliva samples easier to collect than throat swabs.<br>• Confidence in obtaining good samples.<br>• Familiarity with SS following experience of COVID-19 ST	• SS generally not pleasant and sometimes uncomfortable, although still happy to complete them<br>• Unanticipated practical difficulties, e.g., with producing enough saliva, not touching surrounding areas when throat swabbing<br>• Someone else assisting or collecting the sample might be able to obtain an accurate sample	Participants were generally happy and confident in SS by throat swabs and collecting saliva samples, with saliva samples perceived as slightly more acceptable than throat swabbing.
**Nicholls 2022** (31)<br>HIV<br>UK	Interviews n=29 cisgender men MSM from Asian, Black and Latin American backgrounds	• performance/usability (e.g., ease use)<br>• settings & logistics (e.g., good packaging)<br>• personal relief/motivations (e.g., sense of being in control and autonomy without interfacing with HCP; avoid judgmental contact with clinics; HIV ST could replace routine STIs testing)	• attitudes & concerns (e.g., untrustworthiness in ST accuracy)	Overall, HIV ST was felt to be empowering as leading to increases in self-efficacy and provided the opportunity to test without accessing health services.
**Powell 2016** (19)<br>Chlamydia<br>UK	Interviews with self-testers n=11	• communication, training & instructions (e.g., free t-shirts to tell others they tested)<br>• performance/usability (e.g., ease of use)<br>• personal relief/motivations (e.g., privacy; avoid a physical examination by	• attitudes & concerns (e.g., uncertainty about correct performance and ST accuracy; anxiety of testing positive in the absence of HPCs for reassurance and advice; anxiety in relation to couple relationship - seen as suggesting	These exploratory findings suggest that ST independently of formal health care systems may no more negatively impact people than being tested by health care professionals. Findings suggest that interventions which increase confidence in using STs and that

**Author, year** (reference), infection, country	**Sample**	**Enablers, benefits of SS/ST**	**Barriers to, cons of SS/ST**	**Study conclusions related to acceptability of SS/ST**
		HCP; avoid going to a clinic perceived as stigmatising; could ST with a friend instead; reassurance about asymptomatic infection; sense of control and autonomy)<br>• settings & logistics (e.g., save time; no need for a medical appointment)	promiscuity, unfaithfulness, or indicating a likelihood of being infected)	provide reassurance of test accuracy may increase ST intentions.
**Rohr 2022** (56)<br>Covid-19<br>Germany	Interviews with self-testers n=37, with test rejecters n=29	• communication, training & instructions (e.g., useful study information in the brochure; clear pictorial and video instructions which increased self-efficacy; convenient to receive results online; trusted communication material)<br>• performance/usability (e.g., ease use; child-friendly)<br>• personal relief/motivations (e.g., know about own health status)<br>• settings & logistics (e.g., convenient to test at home)<br>• social utility (e.g., testing at home reduces risk for transmission)<br>• attitudes & beliefs (e.g., trust in SS/ST accuracy; reliable study's institutional affiliation)	• attitudes & concerns (e.g., uncertainty about correct performance and ST accuracy)<br>• communication, training & instructions (e.g., biohazard symbol raised concerns about safety of test; how results would be communicated unclear)<br>• experience/procedure (e.g., procedure was disgusting; SS in the morning on an empty stomach; results' waiting times)<br>• performance/usability (e.g., SS method perceived as too complex)<br>• settings & logistics (e.g., over-use of plastic in the packaging)	Most participants who completed the SS reported it was easy to use, trustworthy, and convenient. There was lack of consensus of preferred SS method; some preferred gargle others swabbing.
**Wachinger 2021** (62)<br>Covid-19<br>Germany	Interviews<br>n=6 school stakeholders and staff,<br>n=10 pupils,<br>n=10 parents	• personal relief/motivation (e.g., sense of safety)<br>• experience & procedure (e.g., good testing experiences)<br>• social utility (e.g., testing at home reduces risk of transmission; contribute to COVID-19 research)	• experience/procedure (e.g., need to acquaint testing, which initially is annoying and scaring, to become like 'like brushing teeth')	The screening was highly accepted and viewed as feasible among interviewed participants. Negative consequences (e.g., more risk-taking behaviour) were not observed.

**Author, year** (reference), infection, country	**Sample**	**Enablers, benefits of SS/ST**	**Barriers to, cons of SS/ST**	**Study conclusions related to acceptability of SS/ST**
**Wanat 2021** (13)<br>Covid-19<br>UK	Interviews n=18	• communication, training & instructions (e.g., training valuable)<br>• personal relief/motivations (e.g., increased confidence engaging in activities that were allowed)<br>• social utility (e.g., reduced fear of accidentally infecting other, reduce virus spreading; self-isolate; contribute to research)	• attitudes & concerns (e.g., uncertainty about correct performance and resulting ST results' accuracy)<br>•experience/procedure (e.g., temporary discomfort)<br>•settings & logistics (e.g., testing on campus inconvenient and frustrating, with fear of infecting others and lack of mirror)	Interviewees were motivated to conduct once-a-week testing as they wanted to know whether or not they were infected. Most participants accepted that the test was not 100% accurate. Importantly, most reported that a negative test result did not change their behaviour, but some did allow more social contact because they felt reassure. Participants valued the training, but some individuals still doubted their ability to do the test. Participants also raised the importance of safety and convenience when attending for tests on site.
**Witzel 2019** (27)<br>HIV<br>UK	Survey n=494;<br>Interviews n=10	• communication, training & instructions (e.g., clear instructions)<br>• experience & procedure (e.g., less anxiety for more experienced testers due to the relative immediacy of results)<br>• performance/usability (e.g., ease of use; results easy to read)<br>• personal relief/motivations (e.g., use new technology)<br>• settings & logistics (e.g., increased privacy, and confidentiality; increased opportunities and accessibility to ST)<br>• social utility (e.g., be a of good citizen; contributing to gay community and science more)	• attitudes & concerns (e.g., about correct performance; anxiety about conducting the HIV ST alone; HIV ST is disconnected from established care pathways)<br>• experience/procedure (e.g., the 15-minute wait for results exceptionally anxious time)<br>• performance/usability (e.g., blood collection via lancet)	Cisgender men who have sex with men participating in this study reported a good overall experience of the intervention.
**Witzel 2020** (28)<br>HIV<br>UK	Interviews n=37<br>MSM	• performance/usability (e.g., ease of use)<br>• personal relief/motivations (e.g., appeal to use new technology; reassurance	• attitudes & concerns (e.g. anxiety about conducting the HIV ST alone, especially infrequent testers; untrustworthiness in	In nearly all cases, HIV ST reduced barriers to testing, which related to either stigma and privacy issues, or convenience and

Author, year (reference), infection, country	Sample	Enablers, benefits of SS/ST	Barriers to, cons of SS/ST	Study conclusions related to acceptability of SS/ST
		after a sexual risk event; avoid stigmatisation, reduce anxiety and overcome privacy concerns, especially infrequent testers; sense of being in control)<br>• settings & logistics (e.g., convenient to test at home, which is a comfortable location)	ST accuracy, especially more frequent testers and those testing positive)<br>• communication, training & instructions (e.g., instructions were not sufficiently clear to guide use)<br>• experience/procedure (e.g., the 15-minute wait for results more anxious time than in the clinic)<br>• performance/usability (e.g., difficulty using the lancet)	opportunity cost. Concerns regarding kit reliability typically resolved following a negative result but persisted for those who tested positive.
**Witzel 2020** (29)<br>HIV<br>UK	N=37 interviews:<br>N=14 with MSM,<br>n=11 with inexperienced testers,<br>n=12 with opportunistic adapters<br><br>*This paper has delineated participants based on testing experience to identify similarities / differences in motivations and experience*	• personal relief/motivations for opportunistic adaptors (e.g., more convenient and fitted around their lifestyle better - this was secondary benefit for inexperienced testers & pro-testers)<br>• personal relief/motivations for inexperienced testers and pro-testers (e.g., reduce stigmatisation and shame, and overcome privacy concerns; sense of being in control - opportunistic adapters did not have privacy concerns)		Analysis developed a testing typology, inexperienced testers, pro-testers, and opportunistic adapters. HIV ST was acceptable to all participants, but their motivations and experiences differed.
**Witzel 2021** (30)<br>HIV<br>UK	Survey n=46;<br>Interviews n=20	• communication, training & instructions (e.g., clear instructions)<br>• performance/usability (e.g., ease of use; results easy to read)	• performance/usability (e.g., difficulty using the lancet, errors in correctly inserting test stick into the pot containing reagent)	This work adds weight to the conclusion that HIV ST is highly acceptable to trans people in a range of settings. The primary driver of HIV ST uptake for trans people was

**Author, year** (reference), infection, country	**Sample**	**Enablers, benefits of SS/ST**	**Barriers to, cons of SS/ST**	**Study conclusions related to acceptability of SS/ST**
		• personal relief/motivations (e.g., ST allows avoiding contact with healthcare system where lack of necessary skills, empathy and cultural competence to meet the needs of trans people were experienced; use of new technology; sense of being in control)<br>• setting & logistics (e.g., privacy)<br>• social utility (e.g., contributing to the wider acceptability of HIV ST)		issues with inaccessible and inappropriate clinical services which created pronounced barriers to accessing testing and care.

Supplementary Table S4: Summary of findings from included non-peer-reviewed reports

Author, year (reference), title<br><br>study design, infection	Summary of relevant findings related to acceptability, usability and motivations for SS/ST
**Seguin 2018** (20)<br>"Self-sampling kits to increase HIV testing among black Africans in the UK"<br><br>MM, HIV	*Quantitative findings (62/65 respondents returned acceptability questionnaire):*<br>• Instructional video: watched by 19/62 (32.2%) of whom 100% found the video useful and 83.3% stated it made them feel more confident.<br>• Instructions of kit were easy to understand: 82% reported 'easy' or 'very easy'.<br>• Majority (73.2%) were willing to use the kit again.<br>• How participants felt about taking sample themselves: 60.7% felt 'comfortable or 'very comfortable'.<br>• 34.5% reported it was unacceptable to target black African people specifically for SS.<br><br>*Qualitative findings (21 interviews):*<br>• Barriers to the acceptability of SS kits:<br>○ fear of needles,<br>○ insufficient blood flow, issues with the vial and difficulties collecting blood and dropping into the vial, issues with follow-up for insufficient samples,<br>○ stigma, taboo and fear regarding HIV and HIV testing (not wanting to find out HIV status).<br>• Facilitators of the acceptability of SS kits:<br>○ convenience,<br>○ non-threatening lancet,<br>○ clear instructions,<br>○ trust in distributor of kits,<br>○ awareness-raising of HIV testing and treatment.<br>• Most interviewees were not personally offended or discriminated by the intervention being targeted at black African people specifically; one felt offended (finding it racist) and some were unaware the intervention was targeted.
**UKHSA 2022** (22)<br>"COVID-19 PCR: home-testing experience of blind and partially sighted people"<br><br>MM, COVID-19	*Interviews with 29 blind and partially sighted (BPS) and a survey with 69 BPS people.*<br>• BPS people want to complete the test independently without having to rely on friends and family. People expect the call centre to be able to assist with a broad range of issues across the user journey.<br>• Challenges experienced by BPS people ordering and using PCR tests were reported (1st evaluation):<br>○ Findings and reading barcode numbers for registration and courier pickup was almost impossible for participants without assistance.<br>○ Identification of some kit components was challenging without assistance.

	○ The swab test was considered unpleasant but intuitive for participants.<br>○ Sealing biohazard bag and folding the box were very hard for participants.<br>○ Arranging courier collection was challenging because it required participants to be able to read the number on the label.<br>• After a live video assistance was introduced using the Be My Eyes app and improvements to the packaging and return box were made (2nd evaluation), BPS participants reported:<br>○ Positive experience of the quality of assistance provided by the Be My Eyes service, and many felt they wouldn't have been able to complete the home test without assistance.<br>○ Step-by-step verbal guidance helped reassure participants and reduce their anxiety.<br>○ Live video assistance was especially helpful for kit registration, locating and reading the test kit barcodes, and assembling the returns packaging.<br>○ Assembling the returns packaging remained challenging and participants were uncertain whether their attempts to self-assemble boxes were robust enough to protect the sample during shipping.<br>○ The surveyed 69 participants identified the following steps as particularly challenging: using the instructions (45%), registering the kit (55%), collecting the swab (42%), packaging the sample (41%).
**UKHSA 2022** (21)<br>"COVID-19 LFD: self-testing experience of blind and partially sighted people"<br><br>MM, COVID-19	*8 BPS people* performed ST and assessed the difficulty levels of each step of ST:<br>1. Receiving LFD test kit – overall scored as easy<br>2. Preparing test area – minor issues<br>3. Checking test kit contents – **major issues** (one participant withdrew after this stage) – users reported confusion around test kit contents and contrast between components.<br>4. Peeling the seal off the top of the extraction tube – **major issues** – removing the firmly stuck foil cover was problematic.<br>5. Placing filled tube into extraction tube holder – minor issues<br>6. Identifying swab and opening the packet – minor issues<br>7. Self-swabbing both nostrils – no issues/ easy<br>8. Transferring the sample from the swab to the extraction tube – minor issues<br>9. Closing dropper tip of extraction tube – no issues<br>10. Squeezing 4 drops of liquid onto the test cassette's sample well – **major issues** – users had multiple issues applying the sample to the test strip, with difficulties distinguishing the sample well from the results well and determining if the appropriate sample volume had been applied. This was the most challenging step for both the BPS participants and Be My Eyes agents.<br>11. Waiting 15 minutes for the result – no issues<br>12. Interpreting the test result (done by the agents via a video call) – minor issues<br>13. Understanding the implications of the result – no issues.<br>BPS participants experienced challenges in conducting the tests while holding their smartphone as some parts of the process required them to use both hands. This limited the ability of the agent to observe and provided assistance. BPS participants and agents suggested advising, prior to using the Be My Eyes service, to use a hands-free setup for the BPS participants' smartphone or camera device and to include information about preparing the testing area to avoid white or pale testing areas.

**UKHSA 2023** (23)<br>"COVID-19: general public testing behaviours"<br><br>MM, COVID-19	*Quantitative findings:*<br>• 59% of 2000 surveyed people (aged 16 and over) perceived LFDs as easy to take (poll from May 2021).<br>• 'Surveys showed that trust in LFDs had changed over time and that LFDs had increasingly become accepted as sufficient to check for COVID-19.' (p. 11)<br>• By January 2022, 77% of 1000 surveyed people in England reported thinking that LFDs provide accurate results (compared to 86% thinking that PCR tests provide accurate results).<br>• The main reported reasons for testing were (based on min. 94 respondents per wave):<br>o Testing because one thought they had symptoms and wanted to check if it was COVID-19.<br>o Testing to enable meeting with friends, family or attending events.<br>o Testing because one was asked to test by their employer or school, college or university.<br>o Testing because one was in contact with someone who tested positive or may have a regular contact with virus.<br>• The main reasons for testing changed over time: in May 2021 it was more likely to be asymptomatic precautionary testing; then testing due to experiencing symptoms; in December 2021 it was to enable meeting people and attending events; then by February 2023 testing for general reassurance, because it was seen as the 'right thing to do' or due to contact with a suspected COVID-19 case.<br><br>*Qualitative findings:*<br>• LFDs were perceived to give users autonomy and control to test where and when they liked (based on 4 focus groups).<br>• People testing to enable social mixing (meeting people and attending events) in December 2021/January 2022 because they did not want another Christmas disrupted by COVID-19.
**UKHSA 2023** (24)<br>"Testing for COVID-19 using saliva: case studies in vulnerable settings"<br><br>MM, COVID-19	*Special Educational Needs and Disabilities (SEND) settings – a survey with 2983 teachers, students and parents/guardians, and 21 interviews:*<br>• The majority of students (77.1%) preferred the saliva sampling; 14.3% preferred swabs, and 8.6% were not sure.<br>• Survey responses showed 68% of staff and 40% of students found it extremely/somewhat easy to collect the saliva sample.<br>• Challenges collecting saliva samples included not being able to physically spit, not producing enough saliva, and difficulty explaining the process to students with different cognitive abilities (interview). Additional, but less frequently reported challenges included a child who found the process "disgusting" and another who having learned how to spit, then continued spitting around the house (interview).<br>• Nearly all parents expressed preference for saliva-based sampling over swab-based sampling (interview).<br>• Saliva sampling was considered less invasive and less onerous (one sample per week instead of two swab-based samples) (interview)<br>• Some perceived saliva sampling to be more accurate that lateral flow devices (LFD) (interview).<br>• A small number (3/17) of parents reported saliva-based testing enabled them to test their child which otherwise they would/could not have done. However, most would have tested their child regardless of sampling type if required to do so (interview).<br>Overall, saliva collection was preferred by parents and students, but it was not easy to perform. Even among those who preferred saliva sampling, the benefits were often unrelated to the method itself (e.g., only having to collect one sample per week, not having to upload results).

	Prison setting – survey with 37 staff and prisoners:<br>• Survey respondents (all staff) indicated it was easy to collect the saliva sample (94.6%) and preferred saliva sampling to swab (97.3%).<br>• However, saliva sampling does not 'unlock' testing for staff members as most (89.2%) reported routinely testing with LFDs or PCR prior to pilot.<br>Staff who actively participated reported a preference for saliva-based sampling. However, uptake was low indicating purported ease of sampling does not mitigate factors causing low uptake in this group.
**UKHSA 2023** (25)<br>"COVID-19: assessment of swabs available for testing children under 5 years of age"<br><br>Survey, COVID-19	*Survey (number of participants unclear)*<br>• Testing tolerance: The mini-tip swab had higher incidence of distress reported (78%) when compared to standard swab (25%) (although greater proportion were successful at collecting sufficient sample with mini-tip swab). Most did not describe nature of distress, but 57% of incidents of distress with the mini-tip involved crying and 9.3% involved discomfort.<br>• Ease of use: majority reported mini-tip swab (77%) and standard swabs (75%) were not challenging to use. Of those who responded, 'challenging but completed', 22% were using standard swab and 23% for mini-tip swab.<br>• Location of swab: the success rate for 'nose and throat' swabbing was higher for mini-tip swab (84%) than standard swab (68%). 31% of respondents indicated swabbing the nose only. The most common reason was the child became too uncomfortable/distressed during attempts to swab their throat.<br>Benefits of mini-tip swab viewed as limited and were not thought to justify establishing a bespoke testing programme.
**UKHSA 2024** (26)<br>"Mass asymptomatic COVID-19 testing in schools, colleges and HE institutions"<br><br>Survey, COVID-19	*Higher education settings – survey with 2214 students:*<br>• Student satisfaction with testing was high (93%) and the intention to repeat test on post-winter break return was also high (75%).<br>• Over 85% of students reported it was easy to locate relevant information about testing and to queue safely to take the test, site accessibility was good, and it was easy to understand test results.<br>• The most commonly endorsed reasons for testing were (a) to protect themselves and others by reducing the spread (41%); (b) as a way to return home for the winter break (40%); and (c) to give peace of mind (12%).<br>• Students also voiced concerns about the efficacy of LFDs and the impact on their academic work and life if they tested positive (open text box).<br><br>*Schools and colleges – survey (number of participants unclear):*<br>• Being regularly tested made 87% of pupils feel more confident about returning to attend school in person. Parents also reported feeling more confident (data not reported).<br>• Discomfort and potential inaccuracy of testing were the most frequently reported concerns about testing from pupils (data not reported).<br>These studies showed it was possible to quickly establish asymptomatic testing in schools and universities for Covid-19 and that a large number of pupils and students took part.

Supplementary Table S5: Quality appraisal of peer-reviewed studies conducted in Europe

Part A – Quantitative descriptive studies

Study (reference) (country)	Infection type	Quantitative descriptive study design				
		Is the sampling strategy relevant to address the research question?	Is the sample representative of the target population?	Are the measurements appropriate?	Is the risk of nonresponse bias low?	Is the statistical analysis appropriate to answer the research question?
Agusti 2022 (32) (Spain)	RTI (Covid-19)	yes	can’t tell	yes	can’t tell	yes
Aiano 2021 (2) (UK)	RTI (Covid-19)	yes	can’t tell	yes	no	yes
Baetselier 2019 (33) (Belgium)	STIs	yes	no	yes	no	Yes
Castell 2014 (35) (Germany)	STIs	yes	yes	yes	no	Yes
Camus 2021 (34) (France)	Genital infections	yes	can’t tell	yes	yes	yes
Colom-Cadena 2022 (36) (Spain)	RTI (Covid-19)	yes	can't tell	yes	no	yes
Davies 2021 (7) (UK)	RTI (Covid-19)	yes	yes	yes	yes	yes
Flipse 2022 (38) (Netherlands)	Respiratory microorganisms	yes	can't tell	yes	yes	yes
Gillam 2021 (11) (UK)	RTI (Covid-19)	yes	yes	yes	no	yes
Grandahl 2020 (40) (Sweden)	STIs	yes	yes	yes	no	yes
Haussig 2019 (41) (Germany)	RTIs	yes	yes	yes	no	yes
Hoehl 2021 (42) (Germany)	RTI (Covid-19)	yes	can't tell	yes	can't tell	yes

Iruzubieta 2021 (43) (Spain)	RTI (Covid-19)	yes	yes	yes	yes	yes
Jing 2021 (14) (UK)	RTI (Covid-19)	yes	yes	yes	yes	yes
Jing 2022 (15) (UK)	RTI (Covid-19)	yes	yes	yes	no	yes
Lafort 2023 (44) (Belgium)	RTI (Covid-19)	yes	yes	yes	yes	yes
Leenen 2020 (45) (Netherlands)	STIs	yes	no	yes	no	yes
Lindner 2021 (46) (Germany)	RTI (Covid-19)	yes	no	yes	yes	yes
Loo 2017 (47) (Netherlands)	STIs (HIV and other STIs)	yes	yes	yes	can't tell	yes
Marinos 2022 (49) (Greece)	RTI (Covid-19)	yes	yes	yes	yes	yes
Martin 2021 (18) (UK)	RTI (Covid-19)	can't tell	yes	no	no	yes
Møller 2022 (50) (Denmark)	RTI (Covid-19)	yes	yes	yes	can't tell	yes
Nash 2021 (51) (Italy)	HIV	yes	yes	yes	no	yes
Nikolaou 2023 (10) (UK)	Respiratory microorganisms	yes	no	yes	yes	yes
Prazuck 2016 (52) (France)	HIV	yes	can't tell	yes	can't tell	yes
Prazuck 2021 (53) (France)	RTI (Covid-19)	can't tell	no	yes	can't tell	yes
Prazuck 2022 (54) (France)	RTI (Covid-19)	yes	can’t tell	yes	yes	yes
Prinsenberg 2022 (55) (Netherlands)	Hepatitis C	yes	yes	yes	no	Yes
Schuit 2022 (57) (Netherlands)	RTI (Covid-19)	yes	can’t tell	yes	yes	yes
Schuilt 2022 (58) (Netherlands)	RTI (Covid-19)	yes	can’t tell	yes	can’t tell	Yes

Sweeney-Reed 2021 (59) (Germany)	RTI (Covid-19)	yes	can't tell	yes	no	yes
Tonen-Wolyec 2020 (60) (France)	RTI (Covid-19)	yes	yes	yes	yes	yes
Tonen-Wolyec 2021 (61) (France)	RTI (Covid-19)	yes	no	yes	can't tell	yes
Weidlich 2023 (63) (Germany)	STIs	yes	can't tell	yes	can't tell	yes
Wurstle 2021 (64) (Germany)	RTI (Covid-19)	yes	can't tell	yes	can't tell	yes

Part B – Mixed-methods studies

**Study (reference) (country)**	**Infection type**	**Mixed-methods studies**				
		**Is there an adequate rationale for using a mixed methods design to address the research question?**	**Are the different components of the study effectively integrated to answer the research question?**	**Are the outputs of the integration of qualitative and quantitative components adequately interpreted?**	**Are divergences and inconsistencies between quantitative and qualitative results adequately addressed?**	**Do the different components of the study adhere to the quality criteria of each tradition of the methods involved?**
Ahmed-Little 2016 (1) (UK)	HIV	no	yes	yes	no	no
Atchison 2021 (3) (UK)	RTI (Covid-19)	yes	yes	yes	no	yes
Bauld 2023 (4) (UK)	RTI (Covid-19)	yes	yes	yes	no	yes
Blake 2020 (5) (UK)	RTI (Covid-19)	yes	yes	no	no	yes
Brown 2019 (6) (UK)	Chlamydia	no	yes	no	no	yes
Fajardo 2022 (37) (Georgia)	Hepatitis C	yes	yes	yes	no	yes

German 2023 (9) (UK)	Respiratory microorganisms	yes	yes	yes	no	yes
Hirst 2021 (12) (UK)	RTI (Covid-19)	yes	yes	yes	no	yes
Loos 2016 (48) (Belgium)	HIV	yes	yes	yes	no	can't tell
Lown 2023 (17) (UK)	Sore throat	no	yes	yes	yes	no
Witzel 2019 (27) (UK)	HIV	yes	yes	yes	no	yes
Witzel 2021 (30) (UK)	HIV	can't tell	no	can't tell	no	yes

Part C – Qualitative studies

**Study (reference) (country)**	**Infection type**	**Qualitative** studies				
		**Is the qualitative approach appropriate to answer the research question?**	**Are the qualitative data collection methods adequate to address the research question?**	**Are the findings adequately derived from the data?**	**Is the interpretation of results sufficiently substantiated by data?**	**Is there coherence between qualitative data sources, collection, analysis and interpretation?**
Denford 2021 (8) (UK)	RTI (Covid-19)	yes	yes	yes	yes	yes
Grandahl 2020 (39) (Sweden)	STIs	yes	yes	yes	yes	yes
Jones 2021 (16) (UK)	RTI (Covid-19)	yes	Yes	yes	yes	yes
Nicholls 2022 (31) (UK)	HIV	yes	yes	yes	yes	yes
Powell 2016 (19) (UK)	Chlamydia	yes	yes	yes	yes	no
Rohr 2022 (56) (Germany)	RTI (Covid-19)	yes	yes	yes	yes	yes

Wachinger 2021 (62) (Germany)	RTI (Covid-19)	yes	yes	yes	yes	yes
Wanat 2021 (13) (UK)	RTI (Covid-19)	yes	yes	yes	yes	yes
Witzel 2020 (28) (UK)	HIV	yes	yes	yes	yes	yes
Witzel 2020 (29) (UK)	HIV	yes	yes	yes	yes	yes

Supplementary Table S6: Key characteristics of eligible studies conducted outside of Europe

**Author, year (reference), Country, Study design (data collection methods)**	**Infection type/ pathogen, SS/ST, sample type, setting**	**Participants: type & number**	**Key findings related to acceptability**
Altaf 2023 (65),<br>Pakistan,<br>Quantitative (survey)	HIV, ST, oral fluid, community-based organisation's office or at home	150 MSM completed surveys	145(96.6%) participants found the instructions and the kit easy to use on their own, 83(55.3%), therefore found to be acceptable among men who have sex with men.
Arias 2016 (66),<br>Canada,<br>Quantitative (survey)	STIs, SS, Vaginal swab, Clinical setting	184 women from a street youth clinic and therapeutic abortion clinic	Women reported SS with HerSwab to be easy (97.1%), comfortable (88.3%), and preferred to physician-collected sample (80.9%).
Ben Moussa 2022 (67),<br>Morocco,<br>Quantitative (survey)	HIV, ST, Oral fluid-based, Clinical setting	492 MSM and FSW attending clinic and requesting HIV testing	Respondents rated the HIVST as (very) easy to perform (96.7%) and interpret the results (96.5%).
Biello 2021 (68),<br>USA,<br>Mixed Methods (survey + interviews)	HIV, ST, Oral fluid-based, Home<br><br>STI, SS, Capillary blood, urethral, rectal and pharyngeal swabs,<br>Home	MSM who had not tested for HIV in last 3 months and self-reported evidence of being at risk of HIV acquisition ;<br>Survey:<br>19 (HIV ST);<br>41 (STI SS)<br>Interviews: 37	HIV ST respondents rated use very convenient (80%) and being (extremely) confident they had performed the test correctly.<br>STI SS respondents indicated SS was convenient (78%) and protected privacy (83%). Just over half (51%) preferred SS to physician-collected sample (34% indicated neither) and 85% were willing to SS in the future.<br>Qualitative findings likewise found high acceptability and usability of both HIV ST and STI SS. However, participants expressed more challenges and uncertainty related to STI SS.
Billups 2023 (69),<br>USA,<br>Quantitative (survey)	UTI, ST, Urine, Home	150 women with a history of uncomplicated UTI	85% respondents rated the home test was very/somewhat helpful.
Boisvert Moreau 2022 (70),<br>Benin,<br>Part of a larger mixed methods study, this paper reports findings from interviews (including the	HIV, ST, Saliva-based, Clinical setting/home	29 cis women who identify as a sex worker (FSW)	The most frequently stated advantages of HIV ST were its autonomy (51.7%) and privacy (37.9%), and that it is a painless test (34.5%). Nine participants (31.0%) also liked HIV ST for its accessibility. Participants felt confident in their use and interpretation of HIV ST (96.6%) and commended the ease of use. The main barriers included concerns over reliability of saliva-based test (20.7%), possibility of positive

Author, year (reference), Country, Study design (data collection methods)	Infection type/ pathogen, SS/ST, sample type, setting	Participants: type & number	Key findings related to acceptability
numbers of interviewees reporting different views)			users concealing their status (20.7%), and lack of psychological support (10.3%).
Bresser 2022 (71), Lesotho & Zambia, Mixed Methods (survey – relevant data, cognitive interviews – not relevant data)	RTI (COVID-19), ST, Nasal swab, Clinical setting	Inclusion criteria differed between countries – community members that may/may not be symptomatic Survey: 1125 respondents	Overall, 98.2% were very/somewhat satisfied; 99.5% reported it was very/easy to perform COVID-19 ST; 98% found it easy to interpret the results; and 90.4% believed the results were definitely correct. 84.5% relied on pictures to conduct test.
Brown 2016 (72), USA, Mixed Methods (survey + interviews)	HIV, ST, Mouth swab/oral fluid, Clinical setting	People who had unprotected vaginal or anal intercourse in the year prior with a partner of unknown/discordant status; Interviews & survey: n=21 participants	Peer influence and perceived social norms were primary motivational forces for HIV ST. Accuracy of HIVS T (compared with blood testing) was raised by a few participants. Facilitators of HIV ST included ease of use, privacy, and speed. Barriers included cost and lack of immediate and direct contact with medical professional.
Bwalya 2020 (73), Zambia, Qualitative (observations, interviews, focus groups)	HIV, ST, oral/finger-prick blood sample, Home	Community members aged 16+<br>Observations = 22<br>Interviews = 33 (+7 interviews with non-users)<br>Focus groups = 19 participants	HIV ST was viewed as novel and interesting and was found to be easy to use and painless (compared with finger-prick). However, some distrust regarding accuracy (compared with blood-based testing). Benefits included time saving, convenience, privacy, and empowerment. Low literacy levels were a barrier for some.
Bwana 2018 (74), Kenya, Quantitative (survey)	HIV, ST, antibodies, community and clinics	484 venous blood draw, 91 survey	High acceptability, 97.71% participants willing to use again and 98.29% willing to recommend it to a sexual partner. Participants found test instructions easy to understand (96.86%) and test device easy to use (94.29%), 97.71% finding result interpretation straightforward. Only 34.07% correctly identified weak positive results, indicating need for improved guidance on interpretation.
Chan 2023 (75), Malaysia, Quantitative (survey)	Hep. C, ST, Oral fluid and blood-based, Clinical setting	Adults who self-referred to study or were receiving care at study site with unknown HEPC status;<br>Survey = 200	The majority (97%) were willing to use Hep. C ST again in the future. Preferred testing: primary care centre (33.5%), at home (20%), by HCP at clinic (20%). Preference for ST type: oral fluid-based (51.5%), blood-based (26%), no preference (22.5%).

**Author, year (reference), Country, Study design (data collection methods)**	**Infection type/ pathogen, SS/ST, sample type, setting**	**Participants: type & number**	**Key findings related to acceptability**
Chapman 2022 (76), USA, Quantitative (survey)	STIs, SS, Multiple sampling types (Urogenital, pharyngeal, rectal), Clinical setting	People with HIV accessing healthcare clinic<br><br>Survey = 34 (rectal); 60 (pharyngeal)	94% rectal SS strongly/agreed they were comfortable collecting sample. 78% pharyngeal SS strongly/agreed they were comfortable collecting sample. Both sample types were reported as being easy to perform and provided greater privacy than clinician-collected sampling. A minority of respondents indicated concern over not collecting sample correctly.
Chasco 2021 (77), USA, Mixed Methods (interviews – reports relevant data, patient records – not relevant)	HIV, SS, Multiple sampling types (finger-prick blood, urine, pharyngeal and rectal swabs), Home	Patients due follow up visit at pharmacist<br><br>Interviews = 10	Blood collection most difficult to collect but did not deter use or successful collection. No issues reported with other sampling types. Most reported returning samples to be convenient and unproblematic.
Choko 2018 (78), Uganda, Mixed Methods (survey – reports relevant data, interviews with peer distributors – not relevant)	HIV, ST, oral fluid-based sample, Home	Fishermen who had not recently tested for HIV;<br><br>Survey = 89	Most respondents reported being satisfied with HIVST (94.4%), performing HIVST was not hard (98.9%), and trusted the results (92.1%).
Chu 2022 (79), USA, Quantitative (survey)	RTI (COVID-19), ST, anterior nasal swab, Home	Adults and children with PT-PCR-confirmed infection;<br>Survey = 277	Most reported no difficulties collecting nasal swab (95%) and indicated they would be more likely to test for SARS-COV-2 if home tests were available over the counter (96%).
Dawkins 2022 (80), USA, Quantitative (survey)	STI, SS, vaginal swab, Clinical setting	Female patients attending clinic (symptomatic & non-symptomatic);<br>Survey = 55	Majority (% not available) reported STI SS instructions were easy to understand, easy to use, and felt confident collecting sample.
De Schacht 2022 (81), Mozambique, Quantitative (survey)	HIV, ST, sampling type unclear, Home	Pharmacy clients who had purchased and used HIVST;<br>Post-purchase survey = 77 users (additional 5 purchased but not used)	Majority of respondents reported instructions were very/easy to follow (92%), easy to perform (91%), believed they had performed test correctly (100%), believed test result (97%), and preferred HIVST to conventional testing (83%).

**Author, year (reference), Country, Study design (data collection methods)**	**Infection type/ pathogen, SS/ST, sample type, setting**	**Participants: type & number**	**Key findings related to acceptability**
Des Marais 2018 (82)<br>Quantitative (survey)	STIs *(C. trachomatis, N. gonorrhoeae, Trichomonas vaginalis, and Mycoplasma genitalium)* and HPV,<br><br>SS, cervicovaginal swab,<br>Home & clinical setting	Survey = 193 women aged 30-64, infrequently testing, low income and uninsured	Majority (67.6%) reported positive or 22.3% neutral 'overall thoughts' about SS; 96.3% reported willingness to SS again; 93.6% reported no difficulty understanding SS instructions; 97.4% reported no or little physical discomfort; 82.2% reported no pain.
Dovel 2020 (83),<br>Malawi,<br>Quantitative (survey)	HIV,<br>ST,<br>Clinical setting	Patients from outpatient departments at high burden health facilities;<br>Survey = 1052	Majority would use HIVST again (98%) which was higher than acceptability in standard clinician-collected group (82%) and optimised clinician-collected group (85%). All groups indicated desire for additional counselling, but highest (75%) in HIVST group.
Dube 2022 (84),<br>USA,<br>Qualitative (interview)	HIV,<br>SS, Blood sample,<br>Home and clinical setting	Participants referred from larger HIV cure-directed clinical trials;<br><br>Interviews (longitudinal) = 17	Motivations included wanting to contribute to science, help HIV community, and curiosity of new technology. Overall experiences were positive – convenience, providing reassurance, ease of use, clear instructions, minimal pain, perceived safety all mentioned. Challenges centred on collecting sufficient sample, difficulty getting device to stick to or removed from arm.
Edelstein 2020 (85),<br>USA,<br>Quantitative (survey)	HIV,<br>ST, sampling type not stated,<br>Home	Adult cisgender men who have sex with men (MSM) and transgender and gender-nonconforming (TGNC) individuals who had sex with men, who were living in NYC, not previously HIV diagnosed<br><br>Survey = 5903 (respondents who performed HIVST on themselves)	Respondents reported liking the privacy (71%) and convenience (68%) of home HIVST, although only 30% would be willing to pay retail cost of $40.
Francis 2019 (86),<br>Uganda,<br>Mixed-methods (survey + interviews)	Bacterial vaginosis, SS, Vaginal swab,<br>School setting	Girls not menstruating on the day of enrolment<br><br>Survey = 96,<br>Interviews = 83	Respondents reported it was easy to understand the instructions (88% with nurse assistance; 87% without assistance) and to collect the swab (58%; 72%). Across groups 94% willing to SS again in the future.

**Author, year (reference), Country, Study design (data collection methods)**	**Infection type/ pathogen, SS/ST, sample type, setting**	**Participants: type & number**	**Key findings related to acceptability**
			Respondents who collected sample without nurse assistance felt more comfortable (61% vs 56%), more in control (67% vs 48%), more relaxed (50% vs 27%), and less embarrassed (37% vs 56%), but more worried they collected the sample incorrectly (48% vs 27%). Qualitative findings largely map onto the quantitative findings. Notable additional topics included: concern related to loss of virginity and value of education (learning about reproductive health).
Francis 2018 (87), South Africa, Quantitative (survey)	STIs & bacterial vaginosis, SS, vaginal swab (women) / urine (men) / blood (clinician-collected), Home	Young (15-24 years) people<br><br>Survey = 433 (185 men, 248 women)	Both men and women reported instructions for sampling were clear, and they felt comfortable, in control, relaxed, and confident in their ability to collect the sample (% not provided). Participants disagreed it was painful. Most men and over half of women felt embarrassed or anxious.
Gaydos 2020 (88), USA, Quantitative (survey)	STI, SS, Vaginal swab, Pharmacy clinic	Women (18+ years) using a retail pharmacy<br><br>Survey = 312	Majority of respondents felt comfortable collecting sample in pharmacy (95.2%), collecting sample was easy (99.3%), pharmacy sampling was convenient (96.8%). Most indicated preference for self-collection (63.5%) (or no preference 30.1%) at home (26.9%), pharmacy (18.9%) (or no preference 36.9%). Most were willing to pay up to $20 (62.1%).
Gonzalez-Montalvo 2023 (89), USA, Qualitative (focus groups)	RTI (COVID-19), SS, Nasal swab, Community centre	Adults with a history of detention or imprisonment<br><br>Focus group = 17 participants (across 3 focus groups)	Attitudes were generally positive. Main advantage centred around allowing person to test themselves (empowerment). Nasal swab was viewed as acceptable as insertion was shallower. Some thought additional incentives would be required to increase participation.
Gresenguet 2017 (90), Central Africa, Quantitative (survey)	HIV, ST, Finger-prick blood, Clinical settings	Adults seeking to know their HIV status or people living with HIV;<br>Survey = 300	Respondents indicated instructions were very/easy (83.3%), reading & interpretation very/easy of positive result (88.7%), negative result (88.3%), and invalid (86.3%), and ability to surmount difficulties very/easy (84.7%).
Habel 2018 (91), USA, Quantitative (survey)	STI, ST, Urine and vaginal swab, University health centre	Students accessing university health centre	Motivations for ST: risk of STI (62.7%); important way to take care of self (40.5%); part of regular health check (23.2%).

Author, year (reference), Country, Study design (data collection methods)	Infection type/ pathogen, SS/ST, sample type, setting	Participants: type & number	Key findings related to acceptability
		Survey = 185	Benefits of ST: ease (69.2%), no appointment needed (63.2%), confidentiality (38.4%), and cost (26.5%). Majority were overall very/satisfied (96.3%), found ST very/easy (97.8%), found instructions very/easy (96.7%), and would use ST again (80%). No significant differences between men & women.
Haddad 2021 (92),<br>USA,<br>Quantitative (survey)	STI, SS, Rectal swab,<br>Community health centre	MSM and TGW patients attending community health centre;<br>Survey = 33	Majority agreed SS was easy (93.9%), felt no pain (81.8%), and preferred SS to clinician-collected sampling (69.7%) (remaining 30.3% stated no preference).
Harichund 2019 (93),<br>South Africa,<br>Mixed Methods (interviews + participant checklist)	HIV, ST, blood-based sample, Clinical setting	Adults attending clinic with unknown (presumed negative) HIV status;<br>40 participants completed baseline, post-test 1, post-test 2 checklists and interviews	Themes centre around technical feasibility, environmental feasibility, and economic feasibility.<br>Most reported HIV ST was convenient, instructions were easy to understand and perform. Recommendations included refining instructions and providing instructions in local language(s). Cost was identified as a barrier.
Harichund 2019 (94),<br>South Africa,<br>Qualitative (interviews)	HIV, ST, blood-based sample, Clinical setting	Adults attending clinic with unknown (presumed negative) HIV status<br><br>40 participants	Findings are delineated between regular and naive testers. Overall, acceptability of HIV ST was high, with majority (74.4%) preferring HIV ST to clinician-performed sample. Reasons included: greater confidentiality, convenience, autonomy. Challenges/barriers included: absence of counselling and lack of confidence without HCP support.
Harichund 2019 (95),<br>South Africa,<br>Qualitative (interviews)	HIV, ST, blood-based sample, Clinical setting	Adults attending clinic with unknown (presumed negative) HIV status<br><br>40 participants	Explores how HIV counselling and testing (HCT) can complement HIV ST. Counselling: not universally required for HIV ST, but requested by some. Confidence: Some felt HCT was more accurate and reliable than HIV ST. Differences in confidence performing HIV ST observed between naive testers (more confident) than regular testers. Convenience: HIV ST considered more convenient, especially in resource limited setting. Confidentiality: HIV ST offered greater privacy and reassurance that of the confidentiality of the results.

Author, year (reference), Country, Study design (data collection methods)	Infection type/ pathogen, SS/ST, sample type, setting	Participants: type & number	Key findings related to acceptability
Hector 2018 (96), Mozambique, Quantitative (survey)	HIV, ST, Oral sample (plus confirmatory finger-prick blood sample), Clinical setting	Adolescents (16-20 years) who were not pregnant recruited through schools<br><br>Survey = 293	Oral HIV ST was easy to use for 38% of respondents; 78.2% were confident they could perform HIV ST at home unsupervised. However, only 22.4% preferred home-based HIV ST (76.3% preferred hospital). Those (15%) who preferred traditional HIV testing believed blood testing was more trustworthy.
Herbert 2022 (97), USA, Quantitative (survey)	RTI (COVID-19), ST, Anterior nasal swab, Home	Adults with no COVID-19 symptoms recruited through Medical School and Northwestern University; Survey = 206	98.7% would recommend ST to others.
Hergott 2022 (98), Uganda, Quantitative (survey)	Malaria, SS, Dried blood spot, Home	Community members (adults and children) with no malaria symptoms; Survey = 123	Majority reported SS was easy (93%) and most preferred SS at home to clinical sampling (93%). Adult males most likely to report no pain compared to adult females and children (80% vs 59% vs 60%).
Hiransuthikul 2021 (99), Thailand, Quantitative (survey)	STI, SS, urine and swab pharyngeal, rectal, and if applicable, neovaginal compartments, Clinical setting	Transgender women attending clinic who had condomless sex within past 6 months; Survey = 128	Majority reported SS was simple (69.5%), had no concerns over SS (65.6%), would choose SS in the future (96.9%), want home-based SS (68.8%), and were overall satisfied (82.8%). Biggest concern was that incorrect SS might lead to incorrect results (33.6%).
Hoagland 2021 (100), Brazil, Quantitative (survey)	HIV, ST, sample type not stated, Location not stated	MSM and transgender/non-binary individuals taking PrEP, HIV negative; Survey = 185 (users of HIV ST)	Among users of HIV ST, 88.1% were comfortable using it.
Hogenson 2019 (101), USA, Quantitative (survey)	STI, SS, penile-meatal orifice or vaginal swab with option for additional rectal swab, Home	People aged 14+ years<br><br>Survey = 149 (repeat users)	Majority were very/satisfied (92.7%), found SS very/easy to collect (91.3%), and found instructions very/easy (96%).
Horvath 2022 (102), Uganda, Quantitative (survey)	HIV, ST, Oral sample, Home (location at participant's discretion)	Adults at-risk (condomless sex) with negative/ unknown HIV status; Survey = 70	Majority were very/satisfied with HIV ST (99.9%), found instructions very/clear (98.6%), and found it very/easy to complete (98.6%).

**Author, year (reference), Country, Study design (data collection methods)**	**Infection type/ pathogen, SS/ST, sample type, setting**	**Participants: type & number**	**Key findings related to acceptability**
Hsieh 2020 (103),<br>USA,<br>Quantitative (survey)	STI (trichomonas vaginalis), ST, Vaginal swab, Clinical setting	Emergency department patients undergoing gynaecological examination<br><br>Survey = 136	Majority reported that it was not hard at all for them to correctly SS, insert swab, or perform the test (86.8%, 86.8% & 81.6%, respectively). Most would ST at home if available (80.5%) and believed ST was 'just as good' (63.2%) or 'better than' (16.9%) clinician sampling.
Huang 2016 (104),<br>USA,<br>Quantitative (survey)	HIV, ST, Oral sample,<br>Home	Adults (aged 18+) who self-identified as Black/African American or Latino MSM;<br>Survey = 57	Majority reported HIV ST was very/easy (96.5%) and would prefer HIV ST to other testing choices in the future (68.4%).
Hubbard 2020 (105),<br>USA,<br>Quantitative (survey)	HIV, ST, Sample type not stated (presumed oral sample), Home	Adults self-identifying as one of targeted priority populations with unknown HIV status;<br>Survey = 42 (phase 1);<br>26 (phase 2)	Key advantages of HIV ST: did not require visit to clinic (84%), test in private (68%), did not wait long for results (52%). Most preferred HIV ST to clinic-based testing (92%) and half found HIV ST easy to use.
Isabel 2023 (106),<br>Canada,<br>Quantitative (survey)	COVID-19, ST, Swish and gargle saliva sample,<br>Clinical setting	Patients (adults & children) attending clinic for COVID-19 testing (symptomatic or contact with infected person);<br>Survey = 238 (80 children; 158 adults)	Majority reported swish and gargle sample collection was very/comfortable (89.9%). A higher proportion of adults collected sample with minimal help (93.7% vs 81.2%), greater tolerance to salty taste (91.1% vs 67.5%) and were more likely to complete sample testing (93% vs 84.8%) than children.
Ivanova Reipold 2022 (107),<br>Kenya,<br>Quantitative (survey)	Hep. C, ST, Oral fluid sample, Clinical setting	HIV-positive people who inject drugs (index case) or partners who have had sexual intercourse or injected drugs with index case;<br>Survey = 150	95% would be willing to perform Hep. C ST again in the future and most preferred home ST (80%) to ST in health centre (18%), community centre by HCP (16.7%), or screening campaign (1.3%). ST was viewed as easy/very easy (66%) with timing of the test (18%) reported most difficult.
John 2019 (108),<br>USA,<br>Quantitative (survey, including free text questions)	HIV ST, sampling unclear,<br>STI SS, urine and rectal swab, Home	Gay and bisexual men;<br>N=1070	Majority reported urine-based SS (98.9%) and rectal swab SS (89.6%) was easy. Nearly all (99.3%) reported trust in their HIV ST results. Instructional videos for SS/ST were useful (95.6%). Key findings from free text: anxiety while waiting for result, pain/discomfort during rectal swab, inconsistent instructions (HIV ST).

**Author, year (reference), Country, Study design (data collection methods)**	**Infection type/ pathogen, SS/ST, sample type, setting**	**Participants: type & number**	**Key findings related to acceptability**
Johnson 2021 (109),<br>Malawi,<br>Qualitative (interview)	HIV, ST, Oral sample, Home or other location (patient discretion)	Community members aged 30+ years who had performed HIV ST;<br>In-depth interviews = 12 (focus group discussion not with users)	Advantages of HIV ST: convenience, speed of results, easy to perform, time and cost saving, trustworthy results, bring couples closer together.
Jolliff 2022 (110),<br>USA,<br>Mixed Methods (survey – motivations + qualitative – experience)	COVID-19, ST, Nasal swab, Home	Caregivers of children with medical complexity;<br>In-depth interviews & survey = 20	Majority were motivated to test to get early treatment if positive (90%) and to know if child safe to attend school (85%). Themes capture mixed experience of caregivers: 'testing ranges from benign to traumatic', 'testing ranges from simple to complex', 'need for assistance testing'
Junior Matovu 2022 (111),<br>Uganda,<br>Mixed Methods (survey + interviews)	HIV, ST, oral-based,<br>Clinical setting	At risk populations (i.e., MSM, PWID, FSW, fisher folks, adolescent girls and young women) who were active on PrEP;<br>Survey = 366<br>In-depth interviews = 20	Participants reported HIV ST to be time saving, easy to use, cost effective, private, and accessible. It was viewed as an important way to overcome the stigma & discrimination felt at health clinics. Reported more trust in the accuracy of HIV ST than facility-conducted testing. Lack of emotional support if positive identified as a concern. Majority (88.4%) preferred HIV ST to conventional testing and were willing to pay (73.2%) for the test.
Kra 2022 (112),<br>Côte d'Ivoire, Mali and Senegal,<br>Quantitative (survey)<br>ATLAS study	HIV, ST, oral-based,<br>Home	At risk populations (i.e., MSM, PWID, FSW);<br>Survey = 2615	99% of respondents found HIV ST very/easy to use. Additional benefits included: privacy, autonomy, and free cost. Most reported no difficulty understanding instructions (97%), collecting sample (99%), & reading result (98%).
Kumwenda 2021 (113),<br>Malawi,<br>Qualitative (interview)	HIV, ST, Oral Fluid,<br>Home	FSW<br>Interviews = 18	Participants liked that oral-based HIV ST was not invasive and did not involve blood collection (considered painful). Most preferred HIV ST to facility-based testing as it was viewed as more convenient, confidential, and flexible. It was also viewed as a way to avert discriminatory treatment at conventional testing services.
Kurth 2016 (114),<br>Kenya,<br>Quantitative (survey)	HIV, ST, Oral fluid, Home	Adults with unknown/previously negative HIV status;	Most respondents reported HIVST was acceptable (94%) and not difficult to perform (95.4). Most were also

Author, year (reference), Country, Study design (data collection methods)	Infection type/ pathogen, SS/ST, sample type, setting	Participants: type & number	Key findings related to acceptability
		Survey = 239	confident they had used HIV ST and interpreted the results correctly (% not available).
Ky-Zerbo 2022 (115), Côte d'Ivoire, Mali and Senegal, Qualitative (focus group)	HIV, ST, Oral fluid, Home	FSW; Focus group = 73 (HIV ST users; 14 non-users)	HIV ST was viewed as offering more privacy and autonomy than conventional testing and way to avoid stigma. Oral-based HIV ST preferred over blood-based testing and ST preferred over supervised testing.
Ky-Zerbo 2022 (116), Côte d'Ivoire, Mali and Senegal, Qualitative (interviews)	HIV, ST, Oral fluid, Home	MSM, PWID, FSW who acted as secondary distributors of HIV ST among their networks and the secondary recipients; Interviews = 89 (24 secondary recipients)	Approx. half (42%) of secondary users of HIV ST performed ST alone without assistance. Assistance was typically sourced from primary user and could be technical support and/or psychological support. Majority (75%) reported no difficulties. Challenges included collecting the sample (2 participants) and interpreting the results (4 participants).
Lapsley 2023 (117), Kenya, Qualitative (focus groups)	HIV, ST, Oral based, Home	Adolescents and young adults;<br><br>Focus group = 61 (across 9 focus groups)	HIV ST was viewed as offering more privacy and autonomy compared with conventional testing, and offered a way of avoiding stigma from HCPs. Oral testing was viewed as less invasive than blood-based sampling. Participants emphasised the importance of support throughout the testing process and linkage to appropriate follow-up care.
Lee 2024 (118), Singapore, Qualitative (interviews)	COVID-19, ST, nasal swab, retrospective use of ART kits	40 adults aged 21 and over who had used ART kits when made available in Singapore in the post-pandemic period	Facilitators: desire to protect vulnerable individuals; convenience and ease of use of ST kits.<br>Barriers: discomfort during testing, concerns regarding cost, uncertainty about correct testing technique. Perceptions of continued testing: some recommended ongoing ST, especially for new variants and vulnerable groups, others questioned necessity given normalcy of pandemic.
Lee 2023 (119), USA, Quantitative (survey)	COVID-19, SS, Saliva, School setting	Parents and/or their child(ren) and teachers<br><br>Survey = 287	Overall, levels of acceptability were lower among children (58-73.2%) than among adults. Ease of testing children increased with age from 25% for 3-year-olds to 75% for 5-year-olds. 4- and 5-year olds reported it was a fun thing to do (78.3% and 76% respectively).
LeRouge 2022 (120), USA,	Influenza, ST, Nasal swab, Home	People experiencing flu-like symptoms	Majority (81.5%) preferred home ST to provider-collected testing and would use test again (94.6%), regardless of pandemic conditions. Motivation included internal (mental

**Author, year (reference), Country, Study design (data collection methods)**	**Infection type/ pathogen, SS/ST, sample type, setting**	**Participants: type & number**	**Key findings related to acceptability**
Mixed Methods (survey + interviews)		Survey = 254<br>Interviews = 31	distress, illness symptoms, agency) and external (convenience, security and privacy) factors. Most found the instructions easy to follow and were able to swab with no/mild discomfort.
Lippman 2018 (121),<br>South Africa,<br>Quantitative (survey)	HIV, ST, Oral fluid/ finger-prick (participant choice), Home	MSM in area with high prevalence of HIV<br><br>Survey = 127	83% of respondents preferred HIV ST to conventional testing. 64.6% preferred finger-prick HIV ST and 34.2% preferred oral fluid HIV ST. 97.3% reported being very likely to use HIV ST again in the future.
Lippman 2016 (122),<br>USA,<br>Mixed Methods (survey + interview)	HIV, ST, Oral fluid, Home	Transgender women who have had anal sex within the last year and unknown HIV status<br><br>Interview = 11<br>Survey = 44	Majority of respondents reported HIV ST was easy to collect sample (95%), follow instructions (91%), and interpret results (93%). Most were confident they interpreted results correctly (98%) and preferred HIV ST to conventional testing (68%). Qualitative results likewise indicated HIV ST was easy to use, convenient, and painless. HIV ST overcame barriers related to stigma, discomfort, and discrimination experienced by some at health clinics. Cost was identified to be the main barrier to future testing with HIV ST.
Lippmann 2016 (122),<br>USA,<br>Mixed Methods (survey + interviews)	HIV, ST, Oral fluid, home, then visited a health centre for follow-ups.	HIV-negative adult transgender women<br><br>Survey = 50<br>In-depth interviews = 11	94% reported the test was easy to use; 93% - easy to read; 68% preferred HIV ST to clinical testing. Price was a barrier for uptake. A need to reconcile the necessity of privacy vs the need for support that is found at testing sites. Some participants used the kits with others for mutual support.
Liu 2022 (123),<br>China,<br>Qualitative (feedback comments)	HIV, ST, oral fluid and blood, Home	192 comments randomly selected from 21,018 feedback comments	ST was described as convenient, private and accurate. Shipping speed and quality of tools were negative.<br>Stigma: relief after getting a negative result but fear and guilt prior to taking the test. Users associated a negative test with moral living and the intention of living a more disciplined life in the future.
Lora 2020 (124),<br>Malawi,<br>Qualitative (observations, interviews, focus groups and photovoice)	HIV, ST, sampling type not reported, Home and work venues	FSW, home and venue-based, recently self-tested for HIV, of these 19 women knew their status already, with 11 having previously tested positive	Control, confidentiality, and convenience. Home-based FSWs appreciated privacy and respect given by self-testing, which allowed them to test without fear of stigma or judgment from HCPs. Flexibility of HIV ST valued by FSWs with long working hours, test at own convenience without

Author, year (reference), Country, Study design (data collection methods)	Infection type/ pathogen, SS/ST, sample type, setting	Participants: type & number	Key findings related to acceptability
		(and nine of these taking antiretrovirals).<br><br>Interview = 20	the need to visit a clinic. Some FSWs reported HIV ST increased vulnerability by limiting autonomy, as decisions to ST often influenced by peers and venue owners. Especially true for venue-based FSWs who faced coercion from venue owners to ST. Cases of "false-negative" results, causing confusion and highlights need for clear messaging around limitations and correct use of HIV ST.
Luisi 2023 (125),<br>USA,<br>Quantitative (survey)	COVID-19, ST,<br><br>Participants were asked to specify any COVID-19 tests they had received during the previous 6 months (e.g., laboratory, pharmacy, doctor's office, drive through site, government home test kit, or other home test kit).	39,500 addresses were selected in a probability sample; 5,666 respondents to survey.<br>Among 4,654 participants enrolled at baseline, 3,400 (73%) completed an online follow-up survey	Among kit users, 95.5% rated the experience as acceptable, and 23.6% reported being unlikely to have tested without the COVIDTests.gov program. Compared with White persons, Black persons were 72% less likely to use other home test kits. Provision of tests through this well-publicized program likely improved use of COVID-19 home testing and health equity in the USA, particularly among Black persons. More than 95% rated the experience as very acceptable (64.1%) or acceptable (31.4%).
Lyons 2019 (126),<br>Senegal,<br>Quantitative (survey)	HIV, ST, oral. HIV ST kits distributed through venue-based and social network-based approaches. Distribution sites included sex work venues, bars, nightclubs, hot spots, mobile clinics, and health facilities serving key populations	MSM, FSW, MSW, PWID, and transgender women.<br><br>Pre-Test Survey: 1149;<br>Post-Test Survey: 817;<br>HIV ST Kits Distributed: 1839	86.1% found the instructions easy to follow. 74.5% reported being comfortable using HIV ST. 95.2% would recommend HIV ST to others. 94.4% believed their family or friends would use the HIV ST. 30.6% discussed HIV testing with a sexual partner or friend after receiving the HIVST. 54.3% used it at the distribution site, and 45.7% used it at home.
Mabonga 2021 (127),<br>Uganda,<br>Quantitative (survey)	HIV, ST, urine and vaginal samples were tested for NG and CT,<br>Clinics	407 patients screened for CT and NG, with 363 HIV-positive participants included in analysis.	96.9% reported that self-sampling was easy, and 95.6% found it comfortable. 83.8% believed that the results from SS would be accurate. 47.2% preferred SS for future testing, while 23.3% preferred clinician-collected samples, and 29.2% had no preference. Women significantly preferred SS compared to men (56.9% vs. 29.3%). Higher proportion of young adults (55.8% of those aged 17-24) preferred SS compared to older participants.
MacGowan 2018 (128),<br>USA,	HIV, ST, oral fluid, dried blood spot, clinical setting	MSM,<br>22 participants	Most were able to correctly conduct the rapid HIV ST and interpret their results. Participants expressed confidence in

**Author, year (reference), Country, Study design (data collection methods)**	**Infection type/ pathogen, SS/ST, sample type, setting**	**Participants: type & number**	**Key findings related to acceptability**
Quantitative (survey)			performing ST after initial training and practice. Some preferred the oral fluid test (OraQuick) over the finger-stick blood test (Sure Check).
Majam 2021 (129), South Africa, Mixed Methods (survey + post-test interviews)	Hep. C, ST, blood samples (3 different kits), clinical setting, private room to conduct ST.	Each kit, n=52, 18+ years old	Overall usability index 90.7% for bioline. Across all three kits, participants had minimal difficulties following instructions. 90-97.3% correctly interpreted results across all 3 kits. Majority would use the ST again and/or recommend to a friend or sexual partner, across all 3 kits. 97% also stated they would go to a clinic or seek treatment following a positive test. 96% found the device easy to use and preferred ST at home vs going to a clinic.
Majam 2020 (130), South Africa, Mixed Methods (usability checklist + post-test interviews)	HIV, ST, five blood and two oral samples, performed in front of an observer	200 participants interviewed, first time HIV lay self-testers, unknown HIV status	Average usability index for all seven HIV STs was 92.8% (84.2% to 97.6%). Overall, participants could correctly interpret the non-reactive/negative and reactive/positive results for each device. 96% participants would use ST again and recommend to a partner/friend. Mainly difficulty in some steps of sample collection across the seven devices. General dislike of the needle (12%).
Marley 2014 (131), China, Quantitative (survey)	HIV, ST, oral-fluid, observed in clinical setting	FSW, MSM, 1137 respondents	Over half willing to accept HIV ST. Problems in collecting sufficient/appropriate samples. 55.5% doubted accuracy of results. 7.5% recommended simplification of test instructions.
Marley 2021 (132), China, Quantitative (survey)	HIV, ST, oral-fluid read by electronic reader	MSM, 692 respondents	71.2% willing to use electronic reading for HIV ST. Many willing to ST more frequently as well with Smartphone based electronic readers (SER). 80.9% considered home testing right before sex with partner. Cost and confidentiality were deterrents for using SER.
Martinez-Perez 2023 (133), Peru Quantitative (survey)	COVID-19, ST, oral-fluid, rural and urban areas	438 (203 female, 233 male, 2 non-binary) respondents	86.96% accepted COVID-19 ST. Convenience acknowledged. Overall, 51.98% and 27.72% of females answered that they would be ‘likely’ or ‘very likely’ to ST; and 44.21% and 34.33% of males would be ‘likely’ or ‘very likely’ to ST.
Mavhu 2022 (134), Zimbabwe,	HIV, ST vs provider-testing, oral-fluid vs blood sample, Home	18+ years old, unaware of HIV status,	Participants who reported being illiterate were less likely to choose oral-fluid-based ST. Those who chose blood-based

Author, year (reference), Country, Study design (data collection methods)	Infection type/ pathogen, SS/ST, sample type, setting	Participants: type & number	Key findings related to acceptability
Mixed Methods (telephone questionnaire + in-depth interviews)		n=1244, 500 FSW, 251 attend rural clinics, 249 attend urban general clinics, 244 attend voluntary medical male circumcision clinics; 20 in-depth interviews	ST thought it was more accurate 71%. Oral-fluid perceived to be less painful than blood sample. People didn't understand how HIV can be detected in oral-fluid. Hesitancy in ST causing delays.
McMahon 2021 (135), Uganda, Quantitative (survey)	HIV, ST, oral-fluid, clinic-based or community	FSW, not recently tested for HIV, 18+ years old; 31 baseline and 30 follow up	Desirable effects of HIV ST: allows conversation with clients. HIV ST can allow positive testers to grieve, which cannot be done in facility-based clinics. Some difficulties when one tests positive but partner tests negative, inciting violence. Allows increased autonomy and control over condom-less sex with clients and allows an increased reputation of being the 'health sex worker' to attract more clients.
Melnyk 2023 (136), USA, Quantitative (survey)	UTI, ST, vaginal swab, home-based	30 patients called for telephone survey	Process was slower as test had to be mailed, patients had to drive an average of 23.3 mins to drop off test to the lab. On average, uropathogens were resistant to 54% of the 19 tested antibiotics. After treatment with antibiotics per the multiplex PCR UTI sensitivities report, 70% of the patients developed recurrence of UTI symptoms with an average time to patients contacting the health care system of 24.5 days.
Mkopi 2023 (137), Tanzania, Quantitative (survey)	HIV, ST, oral-fluid, rural community	50 households, 139 participants in intervention and 128 in control arm. Participants must live with male head of household + never tested for HIV	All participants reported they would test for HIV. 22.4% agreed that getting tested for HIV in the clinic takes a long time. Female intervention participants were 2.9 times more likely to test for HIV than control participants. Male participants in the intervention arm were 3.2 times more likely to test for HIV than control participants.
Moradi 2022 (138), Iran, Mixed Methods (survey + interviews)	HIV, ST, n/a	930 respondents: FSW 400, MSM 400, transgender 200. 3 surveys + semi-structured interviews with 7 from each group.	50% stated need for counselling. 98% would re-take test, i.e. high acceptability.

Author, year (reference), Country, Study design (data collection methods)	Infection type/ pathogen, SS/ST, sample type, setting	Participants: type & number	Key findings related to acceptability
Mphande 2021 (139), Malawi, Mixed Methods (in-depth interviews + observational journals)	HIV, ST, oral-fluid, test taken in waiting space	56 in-depth interviews and observational journals	Non-users primary reason for not ST: having tested recently. Recommended private spaces to view result outside of view from public, e.g. in waiting area. 25% interview respondents said testing lacked privacy.<br>Rapid results, convenient. Peer support required for HIV ST. Lack of trust in HCPs for confidentiality reasons, HIV ST provides autonomy in whether to disclose results.<br>But also not many opportunities to disclose results, patients uncomfortable to start conversations on HIV test results.
Mujugira 2021 (140), Uganda, Qualitative (interviews)	HIV, ST, oral-fluid, at home	110 HIV-negative cisgender women, cisgender men and transgender FSWs;<br>40 interviews with 30 FSW and 10 partners.	Economic, relational, and sexual empowerment.<br>Made condom-less sex safer. ST with partners to increase trust and to normalise HIV ST. ST at home avoids stigma. Allows FSW to enforce and monitor condom use with clients. MSM seemed to find easier to disclose HIV ST to clients and link them to care. FSW tended to fear violence.
Mukora-Mutseyekwa 2022 (141), Zimbabwe, Mixed Methods (survey + interviews)	HIV, ST, oral-fluid, option of ST in a private room at the clinic or taking the ST kit to their hostel/home	227 participants, university students, 59% female, number of interviews not reported	75% perceived low risk, 74% would trust HIV ST result. Initial scepticism of test distribution due to misinterpretation possibilities. 42% of students who collected HIV ST provided non-existent mobile numbers - relating to stigma.
Nambusi 2023 (142), Botswana, Quantitative (survey)	HIV, Hep. C, Syphilis,<br>ST, finger-prick test, clinical setting	200 young people 18-24 enrolled	Previous HIV history did not influence willingness to ST; all respondent said they would test again. Risk of misdiagnosing oneself without appropriate medical support leading to adverse mental health outcomes. Some concern that HIV ST would replace sexual healthcare services, as could be possibly interpreted by young people. Kits don’t cover all STIs. Emphasis on need for good quality counselling with ST. Important to monitor access and equity following increased demand.
Nangendo 2023 (143), Uganda, Mixed Methods (interviews + survey)	HIV, ST, oral -fluid, at home (given 10 days)	1628 men enrolled, interviews: 6 with HIV positive, 8 with HIV negative	HIV ST made easy to disclose results to sexual partners; preferred to test in morning when not eaten and saliva ‘pure’. HIV ST accessible. 20% needed help during HIV ST. Men perceived blood-based testing as confirmatory to the

Author, year (reference), Country, Study design (data collection methods)	Infection type/ pathogen, SS/ST, sample type, setting	Participants: type & number	Key findings related to acceptability
			saliva test. 68.2% need psychosocial support after HIVST. They were concerned that without access to post-test counselling, HIV ST may not prevent occurrence of self-harm, illicit behaviour, and social conflict among users. Reported of a likelihood of disclosure challenges after positive results since linkage to care is not guaranteed, and thought HIV ST results could be used to decide on long term relationships.
Nelson 2022 (144), USA, Quantitative (survey)	HIV, STI, SS, urogenital / extragenital, rectal, pharyngeal self-swabs, clinical setting	Priority subgroups: MSM, Sexual and gender minorities. N=1382, 68.9% Black or African American	Urine: 18.9% preferred SS vs 19.3% preferred provider swabbing. Rectal: 57% preferred SS vs 43% provider. Pharyngeal: 33.1% preferred self-swab vs 43% provider. Patients reluctant to SS but once instructed on how, more willing to try. Divided responses on comfort of SS.
Ngoc 2023 (145), Vietnam, Quantitative (survey)	HIV, ST, blood-sample, clinical setting	Lay-users, 18+, first-time HIV ST with unknown HIV status. Total n=2399, 600 for each HIV ST device	93% recommend ST. Half preferred using at clinic vs half at home. A statistically significant association between successful ST and higher education level.
Ngure 2017 (146), Kenya, Mixed Methods (interviews, focus groups, interview-administered questionnaires)	HIV, ST, oral-fluid, clinical setting	PrEP users, 222 couples enrolled. 30 interviews (20 male, 10 female), Focus groups separate for men and women	High acceptability, 93% reporting conducting HIV ST at least once. 54.4% didn't share results with anyone. 56.7% preferred oral SS over finger-prick.
Nguyen 2022 (147), USA, Mixed Methods (survey + interviews)	COVID-19, ST, oral-fluid, at home	Survey n=31, Interviews n=15. Participants had to have been unvaccinated for COVID-19 at recruitment	Mean adherence 88%, 71% preferred at-home weekly ST. Half developed a habit of ST. Time – biggest opportunity cost.
O'Byrne 2022 (148), Canada, Quantitative (survey)	HIV, ST, blood-based, at home	18+, not on PrEP, survey respondents = 167/288	Generally favourable feedback, still room for improvements, such as more discrete packaging, widening access to other geographical locations, making instructions more user-friendly and simpler.

Author, year (reference), Country, Study design (data collection methods)	Infection type/ pathogen, SS/ST, sample type, setting	Participants: type & number	Key findings related to acceptability
Ogale 2019 (149), Uganda, Qualitative (interviews)	STIs, SS, vaginal/penile-meatal swab, clinical setting	1825 sexually active HIV positive and negative men and women aged 18–49 years in two communities,<br><br>36 interviews	SS for STI was generally acceptable, valued for privacy, confidentiality, and efficiency. Many participants preferred provider-collected samples due to concerns about SS accuracy, fear of self-harm, and hygiene.<br>Despite concerns, most participants were willing to recommend and redo SS in the future.
Oyaro 2020 (150), Kenya, Mixed Methods (survey + focus groups)	HIV, ST, oral-fluid, clinical setting	Pregnant and post partum women,<br>400 participants,<br>3 focus groups with 18 women + 8 HCPs	95.3% HIV ST users would recommend to other women. Reason women attend hospitals during pregnancy is for HIV testing (e.g., to know their status).
Pant Pai 2014 (151), Canada, Quantitative (survey)	HIV, ST, oral-fluid, clinic	Males and females, 18+ years, n=145	ST highly acceptable among Canadian university students, 81% preferring oral ST over conventional laboratory tests. 99% found ST convenient, and 74% were willing to purchase STs over the counter, despite concerns about test accuracy and linkage to care. 100% agreement between ST results and those verified by HCPs.
Patel 2018 (152), USA, Quantitative (survey)	HIV, ST, oral-fluid, ST kits given in a clinical setting (ED) and then taken home	52 in intervention arm, 48 in control	HIV ST kits increased testing rates among patients who initially declined testing in the ED, 48% of the HIV ST group testing themselves compared to 6% in the control. Approach well-received, all participants in the HIV ST group taking kits home, and it provided an effective way to engage individuals who previously declined HIV testing, many of whom had never tested before.
Pathmanathan 2021 (153), Zimbabwe, Mixed Methods (in-depth interviews + usability testing)	Syphilis, ST, blood-based rapid test, at home	20 MSM, 10 key informants	Trusted this test more as blood-based vs oral-fluid HIV ST. 89.6% usability reported.
Peck 2014 (154), Kenya, Malawi, South Africa,	HIV, ST, oral and finger-prick tests, community and clinics across three countries	10 people per test kit,<br>150: Kenya (49); Malawi (47); South Africa (54). 33 used oral	Less than 25% performed all steps correctly. Positive results interpreted correctly 79.9%.<br>80% felt confident doing test and were likely to do again.

Author, year (reference), Country, Study design (data collection methods)	Infection type/ pathogen, SS/ST, sample type, setting	Participants: type & number	Key findings related to acceptability
Mixed Methods (survey + interviews)		test, 117 used finger-prick test	
Phanuphak 2018 (155), Thailand, Quantitative (survey)	HIV, ST, blood-based, community + online	474 MSM, 99 TGW	Higher levels of education increased pre-test counselling chances. Integrating online HIV ST was feasible.
Phongphiew 2021 (156), Thailand, Qualitative (interviews)	HIV, ST, blood-based, clinical setting	N=90, 15-19 year olds with high HIV risk, MSM (n=45) and TGW (n=45)	Preference for clinic testing vs at home.<br>Adolescents emphasised need for pre-test counselling (80% in favour).
Pierre 2020 (157), Rwanda, Qualitative (interviews)	HIV, ST, oral-fluid, clinical setting	21 men aged 18-39	Generally well-accepted among men, appreciating privacy, quick results time, and convenience.<br>Mixed experiences with the clarity of instructions, some suggesting translations into local languages. Most participants didn't feel the need to visit a health facility after a negative result, some concerns about potential social harms, such as distress from unclear results, and the importance of linkage to care for those who are positive.
Rael 2022 (158), USA, Qualitative (interviews)	HIV/syphilis, ST, blood sample, at home	N=11 TGW in-depth interviews	High acceptability of dual HIV/syphilis ST among TGW. Successful use of ST in various settings, e.g., homes and cars. Varying range of comfort levels with the blood collection process, but acceptability increased with use.
Rao 2022 (159), India, Mixed Methods (interviews + diagnostic test evaluation)	HIV, ST, oral-fluid, clinical setting	98 participants (48 confirmed HIV seroreactive and 50 sero-non-reactive)	High acceptability: comfort, confidentiality, and convenience of ST, particularly preferring saliva over blood. Some participants suggested improvements, including the need for helpline for support. Concerns of potential for psychological distress following positive result and need for a confirmatory test to avoid false security.
Reipold 2021 (160), Egypt, Quantitative (survey)	Hep. C, ST, oral-fluid, observed in clinical setting	116 enrolled	High acceptability, 96.5% willing to ST again and 99% willing to recommend it to family and friends. 53.4% experienced difficulties with removing the cap from the test tube and placing the tube in the stand. Additional support needed to improve usability for those with lower education levels.
Ren 2017 (161), China,	HIV, ST, n/a, online	5,6996 MSM online survey responders	40% rate of HIV ST uptake among MSM in Beijing, 92% of willing to ST in the future. Higher monthly income, prior

Author, year (reference), Country, Study design (data collection methods)	Infection type/ pathogen, SS/ST, sample type, setting	Participants: type & number	Key findings related to acceptability
Quantitative (survey)			experience with HIV voluntary counselling and testing associated with increased HIV ST uptake.
Rick 2021 (162), Brazil, Quantitative (survey)	HIV/STI, ST, clinical setting	838 males, 99% self-identified as cis-gender	Highly accepted, motivators including quick results, privacy, avoiding physical attendance at health units. Barriers: fear of test result and not knowing where to get tested.
Ritchwood 2019 (163), South Africa, Qualitative (focus groups + observations)	HIV, ST, oral-fluid and blood-based (choice), Clinical setting	Women, peers and partners.<br><br>Total n=55 for both phases, 16 in focus group of phase 1. Phase 2b n=40.	More comfort SS saliva than blood, but those who were concerned about accuracy preferred blood-based kit. More than half participants stated they'd like to have someone with them whilst HIV ST. Suggested HIV ST booths in the community may help increased uptake and more privacy outside the home.
Rocha 2019 (164), Brazil, Quantitative (survey)	T. vaginalis (STI), SS, vaginal swab, clinical setting but SS alone	300 women	Many found SS acceptable, with low pain, easy to use. Some preferred a complete gynaecological exam and to have a HCP complete the sampling.
Rochford 2023 (165), Zimbabwe, Mixed Methods (interviews + survey)	STI (CT, NG, T. vaginalis), SS, vaginal swab, clinical setting	Youth (16-24 years old) women.<br>129 women tested,<br>12 in-depth interviews	49.2% 16-19 year olds reported anxiety SS.<br>Most would recommend to a friend. Privacy and clear instructions vital and could be compromised by lack of locks on bathrooms.
Rutty 2020 (166), Zambia, Quantitative (survey)	FGS, SS, vaginal swab/ urinal sample/ cervical swab, at home	603 women	Highly acceptable, 90% preferring to SS at home due to greater privacy.
Saberi 2022 (167), USA, Quantitative (survey)	HIV/COVID-19, SS, dried blood spot (HIV), oropharyngeal swab/urine sample/rectal swab (syphilis, Hep. C, CT, NG), nasopharyngeal (Sars-Cov-2), at home	18+ years adults,<br>92 enrolled	82.2% like to recommend home SS kit to a friend.<br>Many preferred home collection due to convenience and ability to avoid clinic testing.
Sarkar 2016 (168), India, Mixed Methods (survey + interviews)	HIV, ST, oral-fluid,<br>Clinical setting (but ST alone)	Pregnant women, 18+ years, in first trimester.<br><br>Survey: 202<br>In-depth interviews: 32	100% acceptance rate. 90.6%s stated benefits from post-test counselling. 98% deemed pre-test counselling necessary.<br>Most difficult part was inserting sample in buffer solution (13.3%).
Schilling 2023 (169), USA,	COVID-19, ST (AI-enabled diagnostic tool), oral-fluid, at home	N=822,<br>18+ years old	Stronger preference for screening at home with ST kit vs in-clinic and vs reporting results on an app.

Author, year (reference), Country, Study design (data collection methods)	Infection type/ pathogen, SS/ST, sample type, setting	Participants: type & number	Key findings related to acceptability
Quantitative (survey)			Ease of use and convenient. Web-based symptom screening tool well-received in white demographics with black demographics preferring in-person clinic testing.
Schnall 2016 (170), USA, Mixed Methods (survey + interviews)	HIV, ST, oral-fluid. Observed in clinical setting.	21 young adults, at high risk for HIV, 18-24 years old	ST perceived as quick, easy and private, without judgement from HCPs in clinical settings. Some concerns on accuracy and validity due to using saliva rather than blood samples.
Schuster 2023 (171), USA, Quantitative (survey)	COVID-19, ST, oral-fluid, nasal swab, school setting	67 school students and 68 staff, asymptomatic	37% preferred nasal to saliva swab method as faster and easier.
Sharma 2022 (172), USA, Mixed Methods (survey + interviews)	HIV/Bacterial STI, SS, rectal swab, urine sample, pharyngeal swab (gonorrhoea and chlamydia), finger prick (HIV), at home.	GBMSM, n=100, 25 in-depth interviews	High acceptability, convenience, avoiding stigma, ease of use. Sometimes additional support required for, e.g., finger-prick blood testing.
Sievers 2022 (173), USA, Quantitative (survey)	COVID-19, ST, nasal swab, clinical setting	50 rehabilitation residents, 18+ years	Willingness to report positive results in the post-test survey decreased in comparison to pre-test survey. ~25% increase in familiarity with lateral flow testing following first time use, cited ease of use and convenience.
Smith 2016 (174), Australia, Quantitative (survey)	Chlamydia, ST, vaginal swab/urine sample, at home vs in-clinic.	445 respondents, MSM n=165, women n=150, heterosexual men n=130.	Highly acceptable, 65% preferring home retesting due to convenience and ease of use.<br>95% reported no significant problems with at home retesting.
Smith 2016 (175), South Africa, Quantitative (survey)	HIV, ST, blood sample, clinical setting	224 participants, 16-25 years	72.5% respondents not put off by ST. 12 found it distressing to prick themselves. 89.96% would tell others about ST. Younger participants more likely to give higher acceptability ratings.
Soto 2023 (176), USA, Quantitative (survey)	COVID-19, SS, nasal swabs, clinical setting observation	Families with children younger than 7 years, n=21	Little or no difficulty across most families, all felt comfortable using nasal swab and would return samples without problems.
Strong 2021 (177), USA, Quantitative (survey)	COVID-19, SS, nasal swab, clinical setting that simulated a home environment	N=30, no older than 65 years	All 30 participants successfully collected valid samples. Some feedback on clearer instructions relating to label use and how far up the nose to swab.

Author, year (reference), Country, Study design (data collection methods)	Infection type/ pathogen, SS/ST, sample type, setting	Participants: type & number	Key findings related to acceptability
Sullivan 2021 (178), USA, Mixed Methods (survey + interviews)	CT/NG, SS, pharyngeal swab, a rectal swab, and urine sample. At home	GBMSM, n=51 couples completing test; In-depth interviews: n=10 couples	93% reported would be willing to SS a throat again. 35% reported the pharyngeal swab as uncomfortable. Rectal swab most challenging (71% reported not easy to use), some used a water enema prior to sample collection over fears of faecal matter on the sample.
Tonen-Wolyec 2019 (179), Congo, Quantitative (survey)	HIV, ST, blood-based finger prick, clinical setting unsupervised	628 adolescents	91% accepted ST. Acceptability increased once test had been completed from 67.8% to 95.5%. 68% claimed post-test counselling essential vs only 46.5% claiming pre-test counselling was necessary.
Tonen-Wolyec 2020 (180), Congo, Quantitative (survey)	HIV, ST, oral-fluid vs blood-based, clinical setting observed	528 participants	23.1% did not trust the results of the oral-fluid ST, whereas 25.0% had fear when using blood ST. Preference for oral-fluid-based HIV ST over blood-based HIV ST.
Tonen-Wolyec 2021 (181), Congo, Quantitative (survey)	HIV, Hep. C, Hep. B, ST, finger-prick blood sample, at home and clinic	18-49 years of age, at high risk of HIV, Hep. B and Hep. C, and unaware of their serostatus;<br><br>n=251 (60 FSW, 13 IDG, 69 prisoners, 31 MSM)	Overall usability index of 89.2%. 73.3% asked for verbal help. 90.9% found instructions easy to use. 78.5% correctly interpreted ST result. 95.6% and 86.1% would accept to reuse and secondarily distribute STs to partners, friends or family members.<br>Those who used the video and written instructions were more likely to reuse and distribute STs.
Tun 2018 (182), Nigeria, Mixed Methods (survey + follow up interviews)	HIV, ST, oral-fluid, at home	MSM, 17-59 years old; Survey = 319, Follow up interviews = 257	88.5% indicated there was nothing they did not like about ST. 99.6% said they would use the test again. Lack of stigma for homosexuality.
Ursini 2023 (183), Tanzania, Quantitative (survey)	FGS, SS, genital swab and urine sample, private room at study site	211 women aged 18–45 years, having had prior sexual activity	95.3% said the genital swab was comfortable being performed by a female HCP, less than half comfortable with a male HCP. SS and female HCP top two modalities for carrying out testing. >5% would prefer sampling at home, but would do it with further training or female HCP present.
Valentine-Graves 2020 (184), USA/Puerto Rico,	COVID-19, SS, saliva/ oropharyngeal/ dried blood spots. At home, online with counsellor	18+ years old, residing either in the USA or Puerto Rico, recruited online, n=148	90% rated dried sport collection as acceptable. Median acceptability for throat swab and saliva was 5/5.<br>Few comments regarding too high a complexity of instructions and equipment for lay people.

Author, year (reference), Country, Study design (data collection methods)	Infection type/ pathogen, SS/ST, sample type, setting	Participants: type & number	Key findings related to acceptability
Quantitative (survey + verbal comments during observations)			
Volk 2015 (185), Peru and Brazil, Quantitative (survey)	HIV, ST, blood sample, at home	MSM, n=52 in Peru, n=51 in Brazil, 49% <25 years old	98% (90/92) reporting use of ST at the one-month visit. 92% comfortable collecting own blood. 99% would use again in the future, all participants said they would recommend to friends.
Wachinger 2021 (186), Uganda, Qualitative (interviews)	HIV, ST, oral fluid, at home	960 FSW enrolled, 62 interviews with 33 participants	Description of HIV ST allowing FSW to increase financial resources by saving on transport costs and clinic fees + test customers who want to go condom-less. Sharing the ST experience with peers seen as bonding and strengthening social relationships. Preferred to learn of HIV status through HIV ST than facility-based. Concern that HIV being tested for in saliva, when previously told only in the blood.
Wang 2020 (187), China, Quantitative (survey)	HIV, ST, oral-fluid, self-administered test study on-site	510 MSM, 140 MSM recruited online	76.5% reported being willing to use a ST outside of the study, but 56.8% reported unwillingness to purchase oral HIV ST, assuming a cost of $8. 65.5% preferred blood testing.
Ware 2023 (188), Uganda, Qualitative (interview)	HIV, ST, oral-fluid, at home	500 pregnant women living with HIV + their male partners enrolled,<br>Interviews: 45 women + 45 male partners	Difficulties with delivering HIV ST to male partners due to fears of disclosing own HIV status leading to abandonment and relationship strain. Women confident in interpreting but preferred to leave positive results communication HCPs. Emotional burden on women because of responsibility of secondary distribution of HIV ST.
Wilson 2021 (189), Kenya, Quantitative (survey)	HIV, ST, oral-fluid, at home	274, ages 15–24 years old	97% self-reported HIV ST completion. 92% self-tested without assistance. 83% reported HIV ST easy to use. Acceptability of HIV ST services was high, with ≥ 90% reporting that they somewhat or completely agreed that HIV ST met their approval, was pleasing, was welcomed, and was liked. 16% preferred provider-delivered testing reporting it was more accurate and trustworthy + used by friends. 75% wanted post-test counselling.

Author, year (reference), Country, Study design (data collection methods)	Infection type/ pathogen, SS/ST, sample type, setting	Participants: type & number	Key findings related to acceptability
Wirtz 2021 (190), Myanmar, Mixed Methods (survey + interviews)	HIV, ST, oral-fluid/ dried blood spot, clinical setting	MSM, transfeminine individuals; Survey = 577 Interviews = 20	Initial lack of confidence due to misconception that HIV ST cannot be detected in oral fluid. Increased confidence following actual use of the test. Privacy and convenience of ST among 'hidden' MSM.
Wixted 2022 (191), USA, Quantitative (survey)	COVID-19, SS, blood sample, clinical setting	N=100	15.7% samples did not have enough serum for analysis, those who could not collect a sufficient sample were significantly older.<br>5% didn't think instructions were easy to follow.<br>Most thought was convenient, easy to use and painless.
Xu 2022 (192), China, Quantitative (survey + observations)	Hep. C, ST, oral fluid, in community and hospital outpatient clinic.	N=100, MSM, 18+ years old	96% completed the Hep. C ST, 54% completed all steps without assistance. 94% were willing to ST if available. Preference for oral-fluid Hep. C ST in 59% vs blood-based ST 19%.
Yan 2015 (193), China, Quantitative (survey)	HIV, ST, oral fluid/ blood spot	522 MSM	72.8% perceived HIV ST as less accurate compared to hospital provided testing.
Zhang 2021 (194), Australia, Qualitative (interviews)	HIV, ST, sample type not specified, participants asked about their previous experiences	15 gay and bisexual men interviewed	HIV ST seen to supplement clinic-based testing rather than replace it. Need for increased access to ST kits in Australia to ensure increase in testing frequency. Concerns around decreased sensitivity in HIV window period.

Abbreviations used in the table: CT – chlamydia trachomatis, GBMSM – gay, bisexual, men who have sex with men, FGS - Female genital schistosomiasis, FSW- female sex workers, HCP – healthcare professional, HCT – HIV counselling and testing, Hep. – hepatitis, HIV – human immunodeficiency virus, NG – Neisseria gonorrhoeae, MSM – men who have sex with men, MSW – male sex workers, PreEP – Pre-Exposure Prophylaxis, PWID – persons who inject drugs, RTI – respiratory tract infections, STI – sexually transmitted infections, UTI – urinary tract infections, SS – self-sampling, ST – self-testing, TGW – transgender women.

Supplementary Table S7: Summary of enablers and barriers to self-sampling/self-testing reported in studies outside of Europe

Part A - Enablers

**ENABLERS & BENEFITS OF SS/ST** (bold font = unique to non-European studies)	**RTI**	**STI**	**OTHER INFECTIONS**
	**(Reference numbers to non-European studies per infection type and continent)**		
Motivation to determine one's infection status, gain reassurance/ peace of mind, and inform one's behaviour, **safeguard health status/ increased awareness/ knowledge of infection following testing themselves and others**	Africa: (71)<br>Asia: (118)<br>North America: (79,110,120,147)	Africa: (70,78,109,111,115–117,126,135,140–143,165)<br>Asia: (123,159)<br>North America: (67,72,76,77,84,91,105,122,158,170,178,195)<br>Australia: (194)	Africa: HCV: (160)<br>Asia: HCV: (75,192)
Motivation to prevent infection transmission and protect others	Africa: (71)<br>Asia: (118)<br>North America: (79,89,110,120,147)	Africa: (70,73,78,116,135,186)<br>North America: (72,76,77)	
A part of good citizenship, to promote normalisation of SS/ST	North America: (120,147)	Africa: (73)<br>North America: (77)<br>Australia: (194)	
Convenience, practicality, ease of access, flexibility (timing & lifestyle-wise)	Africa: (71)<br>North America: (79,89,110,120,147,191)<br>South America:(133)	Africa: (70,78,93–95,109,111,113,115,117,121,124,134,139–143,146,150,153,154,166,182,196)<br>Asia: (123,155,161,190)<br>Australia: (174,194)<br>North America: (68,72,76,77,80,84,85,92,105,108,148,151,170,172,178,195)	Africa: HCV: (160)<br>Asia: HCV: (75,192)<br>North America: UTI: (136)
Interest in new health technology		Africa: (73,78,116,124)<br>North America: (72,76,77)<br>Australia: (194)	Asia: HCV: (75)
Motivation to contribute to research/ public health initiatives		Australia: (194)<br>North America: (84,172)	

Experience of and familiarity with SS/ST	North America: (79,125)	Asia: (187,193)<br>North America: (77)	Africa: HCV:(107)<br>Asia: HCV: (192)
Control, autonomy over one's health, **empowerment (e.g., by negotiating condom use, allowing users to have control over their infection decisions)**	North America: (120)	Africa: (70,73,94,112,113,115,117,121,124,130,139,140,142,143,146,153,163,165,186)<br>Asia: (123)<br>Australia: (194)<br>North America: (122,151)	Africa: HCV: (197)
Privacy	North America: (120)	Africa: (70,73,81,94,109,111,115,117,121,124,130,135,139–143,150,153,157,163,165,166,182,186,189,196)<br>Asia: (123,155,161,190)<br>Australia: (174,194)<br>North America: (85,105,122,148,151,152,170,178,195)<br>South America: (162,164)	Africa: HCV: (129,160)<br>Asia: HCV: (192)
Confidentiality, a way of avoiding health services & associated judgment & stigma	North America: (79,120)	Africa: (73,78,81,93,94,111,113,115–117,130,134,135,140,142,143,150,153,154,182)<br>Asia: (123,155)<br>North America: (72,76,77,83,84,91,101,105,152,170,172)<br>South America: (100,162)	Africa: Malaria: (98)<br>Asia: HCV: (75)
No or lower cost		Africa: (93,94,111–115,153,182,196)<br>Asia: (123,145,159)<br>North America: (91,151)	
Accessibility, proximity of testing sites, without pre-booked appointments, access to self-tests in the community OR not needing to go to testing sites initially	Asia: (118)<br>North America: (125,167,191)	Africa: (81,96,111,113,143,146,153,166,182,186)<br>Asia: (138,159)<br>Australia: (194)<br>North America: (88,103,122,167,172,195)	
Ease sample self-collection, ease of use of the SS/ST device	Africa: (71)<br>Asia: (118)<br>North America: (106,110,120,169,171,173,176,191)	Africa: (67,70,78,86,87,90,93,96,102,111,112,114–117,127,130,134,139–141,146,154,163,165,166,175,182,189,196,198)<br>Asia: (99,132,145,156,161,168,190,193)<br>Australia: (174,194)<br>North America: (68,72,76,77,80,84,91,92,103,104,108,122,128,144,148,158,170,178)	Africa: HCV: (129,160)<br>Malaria: (98)<br>Asia: HCV: (192)31/10/2024 16:46:00

		South America: (164,185)	
Trust in test accuracy/ result validity	Africa: (71)<br>Asia: (118)<br>North America: (97,147,169)	Africa: (81,96,111,127,134,141,143,153,157,189)<br>Asia: (123,138)<br>North America: (68,103,108)	
Trust in/ credibility of study institution or test providers	Asia: (118)<br>North America: (147)	Africa: (165)<br>Asia: (132)	Africa: HCV: (129)<br>Asia: HCV: (75)
Clear instructions, understanding instructions	North America: (79,120,176)	Africa: (73,74,78,81,83,86,87,90,93,102,120,130,153,154,165,181,182,196)<br>Asia: (155,168)<br>Australia: (174,194)<br>North America: (68,70,72,76,82,84,85,91,122,128,178,195)	
Accompanying materials/ demonstrations/ other languages to assist with SS/ST	Asia: (118)<br>North America: (97,120)	Africa: (115,116,139,146,153,154,163,181)<br>Asia: (138,155)	
Low discomfort/ invasiveness, no pain, needles not necessary	Africa: (71)<br>North America: (79,89,110,120,147,191)	Africa: (73,78,83,113,116,117,127,134,135,150,153,163,165,188,196)<br>Asia: (131,168)<br>North America: (72,76,77,82,84,92,101,122,170,195)<br>South America: (164)	Asia: HCV: (75)
Rapid results	Africa: (71)<br>Asia: (118)<br>North America: (79,110,147)	Africa: (67,73,78,81,115,130,135,139,143,153,157,188)<br>North America: (68,72,76,77,105,108,151,170)<br>Asia: (123,132,156,168)<br>South America: (162)	Africa: HCV: (129)<br>Asia: HCV: (75)
Ease of sample return	North America: (184)<br>South America: (184)	Africa:	Africa:
Ease of reading and understanding ST result, confidence in interpreting results	North America: (147)	Africa: (74,83,90,112,114,130,140,141,154,163,180,182,188,189,198)<br>Asia: (138,145,190,193)<br>North America: (77,122,128,148,151,158,170,178)<br>South America: (185)	Africa: HCV: (107,129,160)<br>Asia: HCV: (192)
**Presence of symptoms warranting the need/ use of a self-test**	North America: (120)		Asia: HCV: (75)
**Motivated by seeing others testing**		Africa: (139)<br>Asia: (193)	
**Ability to safely dispose of the kit**		Africa: (93,163)<br>North America: (158)	

**Additional counselling to prepare for receiving the result, peer support**	Asia: (118)	Africa: (94,95,109,139,141,163,189,198)<br>Asia: (156,168)<br>North America: (122,151,178)	
**Ability to strengthen social relationships by sharing the ST experience with peers/ others**		Africa: (186)	

Part B - Barriers

BARRIERS & CHALLENGES TO **SS/ST** (bold font = unique to non-European studies)	RTI	STI	OTHER INFECTIONS
	**(Reference numbers to non-European studies per infection type and continent)**		
Stigma associated with the infection/ being seen to test/ **packaging not being discreet**	North America: (89)	Africa: (67,73,94,109,114–116,139,141,142,157,182)<br>Asia: (123)<br>North America: (72,76,122,170,178)	Asia: HCV: (75)
Low or no perceived risk of infection, normalcy of infection	Asia: (118)	Africa: (114,116,134,141)<br>Asia: (190)<br>Australia: (174)<br>North America: (152)<br>South America: (185)	Asia: HCV: (192)
Lack of time for SS/ST **or time restrictions on buying a test, no easy access to ST**	North America: (89,110)<br>South America: (133)	Africa: (116,142,146)<br>Asia: (155)<br>Australia: (194)<br>North America: (148,151)<br>South America: (162)	
Cost (prohibitive)	Africa: (71)<br>Asia: (118)<br>South America: (133)	Africa: (111,163,189,198)<br>Asia: (131,132,161,187,190)<br>Australia: (194)<br>North America: (72,85,122,172)	

Complaints about testing sites (e.g., lack of privacy)		Africa: (94,143)	
Concerns about safety/ risks of infection transmission/ contamination at testing sites	North America: (89,184)<br>South America: (184)	Africa: (115,153,196)<br>Asia: (123,155)<br>North America: (199)	
Concerns/ questions & wanting information about test accuracy/ reliability	Asia: (118)<br>North America: (79,110)	Africa: (73,78,81,96,113,124,135,150,163,180,186,189)<br>Asia: (131,168,193)<br>Australia: (194)<br>North America: (68,70,72,76,77,151,170)	Africa: HCV: (160)<br>Asia: HCV: (75,192)
Difficulties with instructions/ **need for videos/pictures**	Africa: (71)<br>North America: (177,184,191)<br>South America: (184)	Africa: (67,94,137,143,154,157,163)<br>Asia: (145,159)<br>North America: (72,77,108,148,170)	Africa: HCV: (160,197)
Difficulties collecting (sufficient) samples, difficult or inconvenient procedures	North America: (110,119,171,173,191)	Africa: (90,94,130,153,154,163,180,181)<br>Asia: (131,145)<br>North America: (68,84,92,108,128,148,158,172)<br>South America: (164,185)	Africa: HCV: (197)<br>Asia: HCV: (192)
Problems with using the equipment	North America: (147,177,184,191)<br>South America:(184)	Africa: (78,81,130,143,153,154,157,163,181,182)<br>Asia: (123,131,145,159)<br>North America: (77,84,108,128,148)<br>South America: (164)	Africa: HCV: (160)<br>Asia: HCV: (75,192)
Concern/ uncertainty if SS/ST performed (in)correctly	Asia: (118)<br>North America: (147)	Africa: (78,86,94,96,113,115,116,135,186)<br>Asia: (99,159)<br>Australia: (174,194)<br>North America: (68,72,76,77,84,108,170,178,195)	Asia: HCV: (75)
Challenges, concerns and inconvenience related to returning samples	North America: (147,167)	Africa: (93)<br>North America: (68)	
Discomfort, pain when collecting samples/ unpleasant sampling	Asia: (118)<br>North America: (106,110,200)	Africa: (86)<br>North America: (108,158,172,178)<br>South America:(164)	Africa: Malaria: (98)
Other negative emotions associated with SS/ST (frustration)		North America: (68)	
Anxiety when performing ST without supervision/ advice,	North America: (79,110,120,191)	Africa: (73,78,81,86,94,95,113,117,130,141,142,146,150,153,154,172,175,180,183,196)	Asia: HCV: (75)

disconnected from care pathway, **anxiety in young children or children with additional needs**		Asia: (145,155)<br>Australia: (194)<br>North America: (68,70,72,76,77,144,172)	
Anxiety waiting for results, perceived long time for results/ **fear of positive result**	North America: (79)	Africa: (78,81,116,157,188)<br>Asia: (123,131,132,190)<br>Australia: (194)<br>North America: (68,72,76,108,178)<br>South America: (162)	
Lack of clarity about how results would be communicated	North America: (79)	Africa: (78)<br>North America: (68,77,84,88)	Asia: HCV: (75)
Having to interpret ST results, difficulties interpreting the results	North America: (79)	Africa: (73,78,81,96,116,124,135,141,143,181)<br>Asia: (131)<br>North America: (85,148)	Asia: HCV: (75)
Uncertainty/ anxiety about what to do when testing positive**/ risks of adverse events following positive result**	North America: (79)	Africa: (73,78,135,163,186)<br>North America: (72,76,77)	Asia: HCV: (75)
**Lack of awareness/ knowledge/ experience of using SS/ST**	Africa: (71)<br>North America: (173)	Africa:<br>(67,70,73,78,81,83,90,93,96,113,115,124,126,134,135,168,180,181,183,188)<br>Asia: (123)<br>North America: (72,76,105)<br>South America: (100)	Asia: HCV: (192)
**Lack of knowledge of the infection itself**		Africa: (70,73,96,113,113,115,134,135,153,183,188)<br>Asia: (123)<br>North America: (72)	Asia: HCV: (192)
**Concerns around confidentiality/ privacy, mistrust regarding testing (e.g. data), mistrust as a result of protected characteristics such as race**	North America: (89,120,125,147)	Africa: (81,94,95,115,116,135,139,142,182,186,198)<br>Asia: (132,159,168)<br>North America: (148)	
**Lack of (psychological) support/ counselling following ST, lack of ability to talk to a clinician,**		Africa: (70,78,81,81,94,111,117,130,134,142,143,150,157,182,186)<br>Asia: (123,159)<br>Australia: (174,194)<br>North America: (148)	Africa: HCV: (129,160)<br>Asia: HCV: (75,192)

**feeling disconnected from health services**			
**Concern around linkage to treatment/care**		Africa: (153)	
**Language barriers when reading instructions**	North America: (119)	Africa: (141,143,157)<br>Asia: (159)	

References

1. Ahmed-Little Y, Bothra V, Cordwell D, Freeman Powell D, Ellis D, Klapper P, et al. Attitudes towards HIV testing via home-sampling kits ordered online (RUClear pilots 2011–12). J Public Health [Internet]. 2016 Sep 17;38(3):585–90.

2. Aiano F, Jones SEI, Amin-Chowdhury Z, Flood J, Okike I, Brent A, et al. Feasibility and acceptability of SARS-CoV-2 testing and surveillance in primary school children in England: Prospective, cross-sectional study. PLOS ONE. 2021;16(8):e0255517.

3. Atchison C, Pristerà P, Cooper E, Papageorgiou V, Redd R, Piggin M, et al. Usability and Acceptability of Home-based Self-testing for Severe Acute Respiratory Syndrome Coronavirus 2 (SARS-CoV-2) Antibodies for Population Surveillance. Clin Infect Dis Off Publ Infect Dis Soc Am. 2021 May 4;72(9):e384–93.

4. Bauld L, Street A, Connelly R, Bevan I, Corti YM, Baxter MS, et al. Students' and staffs' views and experiences of asymptomatic testing on a university campus during the COVID-19 pandemic in Scotland: a mixed methods study. BMJ Open. 2023;13(3):e065021.

5. Blake H, Corner J, Cirelli C, Hassard J, Briggs L, Daly JM, et al. Perceptions and Experiences of the University of Nottingham Pilot SARS-CoV-2 Asymptomatic Testing Service: A Mixed-Methods Study. Int J Environ Res Public Health. 2021;18(1):188.

6. Brown S, Paterson C, Dougall N, Cameron S, Wheelhouse N. Understanding the attitudes and acceptability of extra-genital Chlamydia testing in young women: evaluation of a feasibility study. BMC Public Health [Internet]. 2019 Jul 24 [cited 2024 May 24];19(1):992. Available from: https://doi.org/10.1186/s12889-019-7313-0

7. Davies B, Araghi M, Moshe M, Gao H, Bennet K, Jenkins J, et al. Acceptability, Usability, and Performance of Lateral Flow Immunoassay Tests for Severe Acute Respiratory Syndrome Coronavirus 2 Antibodies: REACT-2 Study of Self-Testing in Nonhealthcare Key Workers. Open Forum Infect Dis [Internet]. 2021 Nov 1 [cited 2024 Feb 21];8(11):ofab496. Available from: https://doi.org/10.1093/ofid/ofab496

8. Denford S, Martin AF, Love N, Ready D, Oliver I, Amlôt R, et al. Engagement With Daily Testing Instead of Self-Isolating in Contacts of Confirmed Cases of SARS-CoV-2: A Qualitative Analysis. Front Public Health [Internet]. 2021 Aug 3 [cited 2024 May 24];9. Available from: https://www.frontiersin.org/journals/public-health/articles/10.3389/fpubh.2021.714041/full

9. German EL, Nabwera HM, Robinson R, Shiham F, Liatsikos K, Parry CM, et al. Participant perceptions and experiences of a novel community-based respiratory longitudinal sampling method in Liverpool, UK: A mixed methods feasibility study. PLOS ONE [Internet]. 2023 Nov 9 [cited 2024 Feb 21];18(11):e0294133. Available from: https://journals.plos.org/plosone/article?id=10.1371/journal.pone.0294133

10. Nikolaou E, German EL, Howard A, Nabwera HM, Matope A, Robinson R, et al. Assessing the use of minimally invasive self-sampling at home for long-term monitoring of the microbiota within UK families. Sci Rep [Internet]. 2023 Oct 24 [cited 2024 Feb 21];13(1):18201. Available from: https://www.nature.com/articles/s41598-023-45574-6

11. Gillam TB, Cole J, Gharbi K, Angiolini E, Barker T, Bickerton P, et al. Norwich COVID-19 testing initiative pilot: evaluating the feasibility of asymptomatic testing on a university campus. J

Public Health [Internet]. 2021 Mar 1 [cited 2024 Feb 26];43(1):82–8. Available from: https://doi.org/10.1093/pubmed/fdaa194

12. Hirst JA, Logan M, Fanshawe TR, Mwandigha L, Wanat M, Vicary C, et al. Feasibility and Acceptability of Community Coronavirus Disease 2019 Testing Strategies (FACTS) in a University Setting. Open Forum Infect Dis [Internet]. 2021 Dec 1 [cited 2024 Feb 26];8(12):ofab495. Available from: https://doi.org/10.1093/ofid/ofab495

13. Wanat M, Logan M, Hirst JA, Vicary C, Lee JJ, Perera R, et al. Perceptions on undertaking regular asymptomatic self-testing for COVID-19 using lateral flow tests: a qualitative study of university students and staff. BMJ Open [Internet]. 2021 Sep 1 [cited 2024 Mar 1];11(9):e053850. Available from: https://bmjopen.bmj.com/content/11/9/e053850

14. Jing M, Bond R, Robertson LJ, Moore J, Kowalczyk A, Price R, et al. User experience analysis of AbC-19 Rapid Test via lateral flow immunoassays for self-administrated SARS-CoV-2 antibody testing. Sci Rep [Internet]. 2021 Jul 7 [cited 2024 Mar 1];11(1):14026. Available from: https://www.nature.com/articles/s41598-021-93262-0

15. Jing M, Bond R, Robertson LJ, Moore J, Kowalczyk A, Price R, et al. User experience of home-based AbC-19 SARS-CoV-2 antibody rapid lateral flow immunoassay test. Sci Rep [Internet]. 2022 Jan 21 [cited 2024 Feb 26];12(1):1173. Available from: https://www.nature.com/articles/s41598-022-05097-y

16. Jones LF, Batteux E, Bonfield S, Bhogal JK, Taylor J, Caiado C, et al. Durham University students' experiences of asymptomatic COVID-19 testing: a qualitative study. BMJ Open [Internet]. 2021 Dec 1 [cited 2024 Mar 11];11(12):e055644. Available from: https://bmjopen.bmj.com/content/11/12/e055644

17. Lown M, Smith KA, Muller I, Woods C, Maund E, Rogers K, et al. Internet Tool to Support Self-Assessment and Self-Swabbing of Sore Throat: Development and Feasibility Study. J Med Internet Res [Internet]. 2023 Dec 8 [cited 2024 May 22];25(1):e39791. Available from: https://www.jmir.org/2023/1/e39791

18. Martin AF, Denford S, Love N, Ready D, Oliver I, Amlôt R, et al. Engagement with daily testing instead of self-isolating in contacts of confirmed cases of SARS-CoV-2. BMC Public Health [Internet]. 2021 Jun 5 [cited 2024 Mar 1];21(1):1067. Available from: https://doi.org/10.1186/s12889-021-11135-7

19. Powell R, Pattison HM, Marriott JF. Perceptions of Self-Testing for Chlamydia: Understanding and Predicting Self-Test Use. Healthcare [Internet]. 2016 Jun [cited 2024 Feb 26];4(2):25. Available from: https://www.mdpi.com/2227-9032/4/2/25

20. Seguin M, Dodds C, Mugweni E, McDaid L, Flowers P, Wayal S, et al. Self-sampling kits to increase HIV testing among black Africans in the UK: the HAUS mixed-methods study. Health Technol Assess [Internet]. 2018 May 2 [cited 2024 Feb 26];22(22):1–158. Available from: https://www.journalslibrary.nihr.ac.uk/hta/hta22220/

21. UK Health Security Agency. COVID-19 LFD: self-testing experience of blind and partially sighted people [Internet]. 2022 Nov [cited 2024 May 28]. Available from: https://www.gov.uk/government/case-studies/covid-19-lfd-self-testing-experience-of-blind-and-partially-sighted-people

22. UK Health Security Agency. COVID-19 PCR: home-testing experience of blind and partially sighted people [Internet]. 2022 Nov [cited 2024 May 28]. Available from: https://www.gov.uk/government/case-studies/covid-19-pcr-home-testing-experience-of-blind-and-partially-sighted-people

23. UK Health Security Agency. COVID-19: general public testing behaviours [Internet]. 2023 Oct [cited 2024 May 28]. Available from: https://www.gov.uk/government/publications/lfd-tests-how-and-why-they-were-used-during-the-pandemic

24. UK Health Security Agency. Testing for COVID-19 using saliva: case studies in vulnerable settings [Internet]. 2023 Apr [cited 2024 May 28]. Available from: https://www.gov.uk/government/publications/testing-for-covid-19-using-saliva-case-studies-in-vulnerable-settings

25. UK Health Security Agency. Assessment of swabs available for testing children under 5 years of age [Internet]. 2023 Feb [cited 2024 May 28]. Available from: https://www.gov.uk/government/publications/covid-19-assessment-of-swabs-available-for-testing-children-under-5-years-of-age/assessment-of-swabs-available-for-testing-children-under-5-years-of-age

26. UK Health Security Agency. Mass asymptomatic COVID-19 testing in schools, colleges and HE institutions [Internet]. 2024 Mar [cited 2024 May 28]. Available from: https://www.gov.uk/government/publications/mass-asymptomatic-covid-19-testing-in-schools-colleges-and-he-institutions

27. Witzel TC, Gabriel MM, McCabe L, Weatherburn P, Gafos M, Speakman A, et al. Pilot phase of an internet-based RCT of HIVST targeting MSM and transgender people in England and Wales: advertising strategies and acceptability of the intervention. BMC Infect Dis [Internet]. 2019 Aug 8 [cited 2024 Mar 1];19(1):699. Available from: https://doi.org/10.1186/s12879-019-4247-1

28. Witzel TC, Bourne A, Burns F, Rodger A, McCabe L, Gabriel M, et al. HIV self-testing intervention experiences and kit usability: results from a qualitative study among men who have sex with men in the SELPHI (Self-Testing Public Health Intervention) randomized controlled trial in England and Wales. HIV Med [Internet]. 2020 [cited 2024 Mar 1];21(3):189–97. Available from: https://onlinelibrary.wiley.com/doi/abs/10.1111/hiv.12818

29. Witzel TC, Weatherburn P, Bourne A, Rodger AJ, Bonell C, Gafos M, et al. Exploring Mechanisms of Action: Using a Testing Typology to Understand Intervention Performance in an HIV Self-Testing RCT in England and Wales. Int J Environ Res Public Health [Internet]. 2020 Jan [cited 2024 Mar 1];17(2):466. Available from: https://www.mdpi.com/1660-4601/17/2/466

30. Witzel TC, Wright T, McCabe L, Gabriel MM, Wolton A, Gafos M, et al. Impact and acceptability of HIV self-testing for trans men and trans women: A mixed-methods subgroup analysis of the SELPHI randomised controlled trial and process evaluation in England and Wales. eClinicalMedicine [Internet]. 2021 Feb 1 [cited 2024 Mar 1];32. Available from: https://www.thelancet.com/journals/eclinm/article/PIIS2589-5370(20)30444-2/fulltext

31. Nicholls EJ, Samba P, McCabe L, Gafos M, Philips AN, Trevelion R, et al. Experiences of and attitudes towards HIV testing for Asian, Black and Latin American men who have sex with men (MSM) in the SELPHI (HIV Self-Testing Public Health Intervention) randomized controlled trial in England and Wales: implications for HIV self-testing. BMC Public Health [Internet]. 2022 Apr 22 [cited 2024 Mar 1];22(1):809. Available from: https://doi.org/10.1186/s12889-022-13189-7

32. Agustí C, Martínez-Riveros H, González V, Fernández-Rivas G, Díaz Y, Montoro-Fernandez M, et al. Feasibility of an online antigen self-testing strategy for SARS-CoV-2 addressed to health care and education professionals in Catalonia (Spain). The TESTA'T- COVID Project. PLOS ONE [Internet]. 2022 Sep 27 [cited 2024 May 24];17(9):e0275006. Available from: https://journals.plos.org/plosone/article?id=10.1371/journal.pone.0275006

33. Baetselier ID, Smet H, Abdellati S, Deken BD, Cuylaerts V, Reyniers T, et al. Evaluation of the 'Colli-Pee', a first-void urine collection device for self-sampling at home for the detection of sexually transmitted infections, versus a routine clinic-based urine collection in a one-to-one comparison study design: efficacy and acceptability among MSM in Belgium. BMJ Open [Internet]. 2019 Apr 1 [cited 2024 May 24];9(4):e028145. Available from: https://bmjopen.bmj.com/content/9/4/e028145

34. Camus C, Penaranda G, Khiri H, Camiade S, Molet L, Lebsir M, et al. Acceptability and efficacy of vaginal self-sampling for genital infection and bacterial vaginosis: A cross-sectional study. PLOS ONE [Internet]. 2021 Nov 18 [cited 2024 May 24];16(11):e0260021. Available from: https://journals.plos.org/plosone/article?id=10.1371/journal.pone.0260021

35. Castell S, Krause G, Schmitt M, Pawlita M, Deleré Y, Obi N, et al. Feasibility and acceptance of cervicovaginal self-sampling within the German National Cohort (Pretest 2). Bundesgesundheitsblatt - Gesundheitsforschung - Gesundheitsschutz [Internet]. 2014 Nov 1 [cited 2024 Feb 26];57(11):1270–6. Available from: https://doi.org/10.1007/s00103-014-2054-9

36. Colom-Cadena A, Martínez-Riveros H, Bordas A, Alonso-García L, Montoro-Fernández M, Romano-deGea P, et al. Feasibility of severe acute respiratory syndrome coronavirus 2 (SARS-CoV-2) antigen self-testing in school and summer camp attendees. Front Pediatr [Internet]. 2023 [cited 2024 Feb 26];10. Available from: https://www.frontiersin.org/articles/10.3389/fped.2022.975454

37. Fajardo E, Watson V, Kumwenda M, Usharidze D, Gogochashvili S, Kakhaberi D, et al. Usability and acceptability of oral-based HCV self-testing among key populations: a mixed-methods evaluation in Tbilisi, Georgia. BMC Infect Dis [Internet]. 2022 May 31 [cited 2024 Feb 26];22(1):510. Available from: https://doi.org/10.1186/s12879-022-07484-2

38. Flipse J, Rossen JWA, Wagenvoort GHJ. Self-collected gargle fluids and nasopharyngeal swabs as a strategy for molecular diagnostics of respiratory viruses. J Clin Virol Plus [Internet]. 2022 Nov 1 [cited 2024 Feb 26];2(4):100116. Available from: https://www.sciencedirect.com/science/article/pii/S2667038022000552

39. Grandahl M, Larsson M, Herrmann B. 'To be on the safe side': a qualitative study regarding users' beliefs and experiences of internet-based self-sampling for Chlamydia trachomatis and Neisseria gonorrhoeae testing. BMJ Open [Internet]. 2020 Dec 1 [cited 2024 Feb 26];10(12):e041340. Available from: https://bmjopen.bmj.com/content/10/12/e041340

40. Grandahl M, Mohammad J, Larsson M, Herrmann B. Users' Opinions of Internet-based Self-sampling Tests for Chlamydia trachomatis and Neisseria gonorrhoeae in Sweden. Acta Derm Venereol [Internet]. 2020 Nov 4 [cited 2024 Feb 26];100(18):1–6. Available from: https://medicaljournalssweden.se/actadv/article/view/1832

41. Haussig JM, Targosz A, Engelhart S, Herzhoff M, Prahm K, Buda S, et al. Feasibility study for the use of self-collected nasal swabs to identify pathogens among participants of a population-

based surveillance system for acute respiratory infections (GrippeWeb-Plus)—Germany, 2016. Influenza Other Respir Viruses [Internet]. 2019 [cited 2024 Mar 4];13(4):319–30. Available from: https://onlinelibrary.wiley.com/doi/abs/10.1111/irv.12644

42. Hoehl* S, Schenk* B, Rudych O, Göttig S, Foppa I, Kohmer N, et al. High-Frequency Self-Testing by Schoolteachers for Sars-Cov-2 Using a Rapid Antigen Test. Dtsch Ärztebl Int [Internet]. 2021 Apr [cited 2024 Mar 8];118(14):252–3. Available from: https://www.ncbi.nlm.nih.gov/pmc/articles/PMC8283684/

43. Iruzubieta P, Fernández-Lanas T, Rasines L, Cayon L, Álvarez-Cancelo A, Santos-Laso A, et al. Feasibility of large-scale population testing for SARS-CoV-2 detection by self-testing at home. Sci Rep [Internet]. 2021 May 10 [cited 2024 Mar 8];11(1):9819. Available from: https://www.nature.com/articles/s41598-021-89236-x

44. Lafort Y, Cornelissen L, Van Cauteren D, Verboven B, Drieskens S, Couvreur S, et al. Were SARS-CoV-2 self-tests used for their intended purpose? The experience in Belgium. BMC Health Serv Res [Internet]. 2023 Jun 29 [cited 2024 Mar 1];23(1):709. Available from: https://doi.org/10.1186/s12913-023-09704-0

45. Leenen J, Hoebe CJPA, Ackens RP, Posthouwer D, van Loo IHM, Wolffs PFG, et al. Pilot implementation of a home-care programme with chlamydia, gonorrhoea, hepatitis B, and syphilis self-sampling in HIV-positive men who have sex with men. BMC Infect Dis [Internet]. 2020 Dec 4 [cited 2024 Mar 1];20(1):925. Available from: https://doi.org/10.1186/s12879-020-05658-4

46. Lindner AK, Nikolai O, Rohardt C, Kausch F, Wintel M, Gertler M, et al. Diagnostic accuracy and feasibility of patient self-testing with a SARS-CoV-2 antigen-detecting rapid test. J Clin Virol [Internet]. 2021 Aug 1 [cited 2024 Feb 27];141:104874. Available from: https://www.sciencedirect.com/science/article/pii/S1386653221001414

47. Loo IHM van, Dukers-Muijrers NHTM, Heuts R, Sande MAB van der, Hoebe CJPA. Screening for HIV, hepatitis B and syphilis on dried blood spots: A promising method to better reach hidden high-risk populations with self-collected sampling. PLOS ONE [Internet]. 2017 Oct 20 [cited 2024 Mar 4];12(10):e0186722. Available from: https://journals.plos.org/plosone/article?id=10.1371/journal.pone.0186722

48. Loos J, Manirankunda L, Platteau T, Albers L, Fransen K, Vermoesen T, et al. Acceptability of a Community-Based Outreach HIV-Testing Intervention Using Oral Fluid Collection Devices and Web-Based HIV Test Result Collection Among Sub-Saharan African Migrants: A Mixed-Method Study. JMIR Public Health Surveill [Internet]. 2016 Aug 4 [cited 2024 Feb 27];2(2):e5519. Available from: https://publichealth.jmir.org/2016/2/e33

49. Marinos G, Lamprinos D, Georgakopoulos P, Oikonomou E, Zoumpoulis G, Garmpis N, et al. Evaluation of Knowledge, Attitudes and Practices Related to Self-Testing Procedure against COVID-19 among Greek Students: A Pilot Study. Int J Environ Res Public Health [Internet]. 2022 Jan [cited 2024 Feb 27];19(8):4559. Available from: https://www.mdpi.com/1660-4601/19/8/4559

50. Møller IJB, Utke AR, Rysgaard UK, Østergaard LJ, Jespersen S. Diagnostic performance, user acceptability, and safety of unsupervised SARS-CoV-2 rapid antigen-detecting tests performed at home. Int J Infect Dis [Internet]. 2022 Mar 1 [cited 2024 Mar 8];116:358–64. Available from: https://www.ijidonline.com/article/S1201-9712(22)00020-0/fulltext#seccesectitle0009

51. Nash SG, Maffeo M, Likatavicius G, Cosmaro L, Rudaitis K, Lapsinov A, et al. Acceptability and usability of HIV self-tests in two European countries: findings from surveys of clients at non-governmental organisations in Lithuania and Italy. BMC Infect Dis [Internet]. 2021 Sep 13 [cited 2024 Feb 27];21(2):844. Available from: https://doi.org/10.1186/s12879-021-06442-8

52. Prazuck T, Karon S, Gubavu C, Andre J, Legall JM, Bouvet E, et al. A Finger-Stick Whole-Blood HIV Self-Test as an HIV Screening Tool Adapted to the General Public. PLOS ONE [Internet]. 2016 Feb 16 [cited 2024 Mar 8];11(2):e0146755. Available from: https://journals.plos.org/plosone/article?id=10.1371/journal.pone.0146755

53. Prazuck T, Van JP, Sinturel F, Levray F, Elie A, Camera D, et al. Evaluation of the practicability of a finger-stick whole-blood SARS-Cov-2 self-test adapted for the general population. PLOS ONE [Internet]. 2021 Jan 28 [cited 2024 Mar 2];16(1):e0245848. Available from: https://journals.plos.org/plosone/article?id=10.1371/journal.pone.0245848

54. Prazuck T, Gravier A, Pires-Roteira D, Theillay A, Pallay S, Colin M, et al. Evaluation of a new "all in one" SARS-CoV-2 antigen-detecting rapid diagnostic test and self-test: Diagnostic performance and usability in child and adult populations. J Med Virol [Internet]. 2022 [cited 2024 May 24];94(9):4097–106. Available from: https://onlinelibrary.wiley.com/doi/abs/10.1002/jmv.27812

55. Prinsenberg T, Schinkel J, Zantkuijl P, Davidovich U, Prins M, van der Valk M. Internet-guided HCV-RNA testing: A promising tool to achieve hepatitis C micro-elimination among men who have sex with men. J Viral Hepat [Internet]. 2022 [cited 2024 Mar 3];29(8):677–84. Available from: https://onlinelibrary.wiley.com/doi/abs/10.1111/jvh.13706

56. Röhr F, Uellner F, Deckert A, Anders S, Burk R, Knop M, et al. From disgusting and complicated to simple and brilliant: Implementation perspectives and lessons learned from users and rejectors of mail-in SARS-CoV-2 gargle tests. Front Public Health [Internet]. 2023 [cited 2024 Mar 4];10. Available from: https://www.frontiersin.org/journals/public-health/articles/10.3389/fpubh.2022.1024525

57. Schuit E, Venekamp RP, Hooft L, Veldhuijzen IK, Bijllaardt W van den, Pas SD, et al. Diagnostic accuracy of covid-19 rapid antigen tests with unsupervised self-sampling in people with symptoms in the omicron period: cross sectional study. BMJ [Internet]. 2022 Sep 14 [cited 2024 May 22];378:e071215. Available from: https://www.bmj.com/content/378/bmj-2022-071215

58. Schuit E, Venekamp RP, Veldhuijzen IK, van den Bijllaardt W, Pas SD, Stohr JJJM, et al. Head-to-head comparison of the accuracy of saliva and nasal rapid antigen SARS-CoV-2 self-testing: cross-sectional study. BMC Med [Internet]. 2022 Oct 24 [cited 2024 May 22];20(1):406. Available from: https://doi.org/10.1186/s12916-022-02603-x

59. Sweeney-Reed CM, Wolff D, Hörnschemeyer S, Faßhauer H, Haase A, Schomburg D, et al. Feasibility of a surveillance programme based on gargle samples and pool testing to prevent SARS-CoV-2 outbreaks in schools. Sci Rep [Internet]. 2021 Sep 30 [cited 2024 Mar 8];11(1):19521. Available from: https://www.nature.com/articles/s41598-021-98849-1

60. Tonen-Wolyec S, Dupont R, Batina-Agasa S, Hayette MP, Bélec L. Capillary whole-blood IgG-IgM COVID-19 self-test as a serological screening tool for SARS-CoV-2 infection adapted to the general public. PLOS ONE [Internet]. 2020 Oct 15 [cited 2024 Mar 4];15(10):e0240779. Available from: https://journals.plos.org/plosone/article?id=10.1371/journal.pone.0240779

61. Tonen-Wolyec S, Dupont R, Awaida N, Batina-Agasa S, Hayette MP, Bélec L. Evaluation of the Practicability of Biosynex Antigen Self-Test COVID-19 AG+ for the Detection of SARS-CoV-2 Nucleocapsid Protein from Self-Collected Nasal Mid-Turbinate Secretions in the General Public in France. Diagnostics [Internet]. 2021 Dec [cited 2024 May 24];11(12):2217. Available from: https://www.mdpi.com/2075-4418/11/12/2217

62. Wachinger J, Schirmer M, Täuber N, McMahon SA, Denkinger CM. Experiences with opt-in, at-home screening for SARS-CoV-2 at a primary school in Germany: an implementation study. BMJ Paediatr Open [Internet]. 2021 Oct 1 [cited 2024 Mar 4];5(1):e001262. Available from: https://bmjpaedsopen.bmj.com/content/5/1/e001262

63. Weidlich S, Schellberg S, Scholten S, Schneider J, Lee M, Rothe K, et al. Evaluation of Self-Collected Versus Health Care Professional (HCP)-Performed Sampling and the Potential Impact on the Diagnostic Results of Asymptomatic Sexually Transmitted Infections (STIs) in High-Risk Individuals. Infect Dis Rep [Internet]. 2023 Oct [cited 2024 Mar 4];15(5):470–7. Available from: https://www.mdpi.com/2036-7449/15/5/47

64. Würstle S, Spinner CD, Voit F, Hoffmann D, Hering S, Weidlich S, et al. Self-sampling versus health care professional-guided swab collection for SARS-CoV-2 testing. Infection [Internet]. 2021 Oct 1 [cited 2024 Mar 4];49(5):927–34. Available from: https://doi.org/10.1007/s15010-021-01614-9

65. Altaf A, Pasha MSK, Wali MS, Majeed A. Acceptability and feasibility of intra-peer and social media approach for HIV self-testing among men who have sex with men in Karachi: lessons from a pilot study. J Pak Med Assoc [Internet]. 2023 Mar 6 [cited 2024 Aug 12];73(3):562–71. Available from: https://ojs.jpma.org.pk/

66. Arias M, Jang D, Gilchrist J, Luinstra K, Li J, Smieja M, et al. Ease, Comfort, and Performance of the HerSwab Vaginal Self-Sampling Device for the Detection of Chlamydia trachomatis and Neisseria gonorrhoeae. Sex Transm Dis. 2016 Feb;43(2):125–9.

67. Ben Moussa A, Belhiba O, Hajouji FZ, El Kettani A, Youbi M, Alami K, et al. Acceptability and usability of oral fluid-based HIV self-testing among female sex workers and men who have sex with men in Morocco. BMC Public Health [Internet]. 2022 Dec 5 [cited 2024 Aug 12];22(1):2266. Available from: https://doi.org/10.1186/s12889-022-14632-5

68. Biello KB, Horvitz C, Mullin S, Mayer KH, Scott H, Coleman K, et al. HIV self-testing and STI self-collection via mobile apps: experiences from two pilot randomized controlled trials of young men who have sex with men. mHealth [Internet]. 2021 Apr 20 [cited 2024 Aug 12];7(0). Available from: https://mhealth.amegroups.org/article/view/50249

69. Billups SJ, Fixen D, Johnson K, Wettergreen SA, Schilling LM. Patient Perception and Impact of Home Test Kits on Health Care Utilization for Urinary Tract Infection. Popul Health Manag [Internet]. 2024 Feb [cited 2024 Aug 12];27(1):44–8. Available from: https://www.liebertpub.com/doi/10.1089/pop.2023.0201

70. Boisvert Moreau M, Kintin FD, Atchekpe S, Batona G, Béhanzin L, Guédou FA, et al. HIV self-testing implementation, distribution and use among female sex workers in Cotonou, Benin: a qualitative evaluation of acceptability and feasibility. BMC Public Health [Internet]. 2022 Mar 26 [cited 2024 Aug 12];22(1):589. Available from: https://doi.org/10.1186/s12889-022-12917-3

71. Bresser M, Erhardt RM, Shanaube K, Simwinga M, Mahlatsi PA, Belus J, et al. Evaluation of COVID-19 antigen rapid diagnostic tests for self-testing in Lesotho and Zambia. PloS One. 2024;19(2):e0280105.

72. Brown W, Carballo-Diéguez A, John RM, Schnall R. Information, Motivation, and Behavioral Skills of High-Risk Young Adults to Use the HIV Self-Test. AIDS Behav. 2016 Sep;20(9):2000–9.

73. Bwalya C, Simwinga M, Hensen B, Gwanu L, Hang'andu A, Mulubwa C, et al. Social response to the delivery of HIV self-testing in households: experiences from four Zambian HPTN 071 (PopART) urban communities. AIDS Res Ther [Internet]. 2020 Jun 11 [cited 2024 Aug 13];17(1):32. Available from: https://doi.org/10.1186/s12981-020-00287-y

74. Bwana P, Ochieng' L, Mwau M. Performance and usability evaluation of the INSTI HIV self-test in Kenya for qualitative detection of antibodies to HIV. PLOS ONE [Internet]. 2018 Sep 13 [cited 2024 Jun 7];13(9):e0202491. Available from: https://journals.plos.org/plosone/article?id=10.1371/journal.pone.0202491

75. Chan HK, Sem X, Ivanova Reipold E, Pannir Selvam SBA, Salleh NA, Mohamad Gani AHB, et al. Usability and acceptability of oral fluid- and blood-based hepatitis C virus self-testing among the general population and men who have sex with men in Malaysia. PLOS Glob Public Health. 2024;4(1):e0001770.

76. Chapman KS, Gadkowski LB, Janelle J, Koehler-Sides G, Nelson JA. Automated Sexual History and Self-Collection of Extragenital Chlamydia and Gonorrhea Improve Detection of Bacterial Sexually Transmitted Infections in People with HIV. AIDS Patient Care STDs [Internet]. 2022 Nov [cited 2024 Aug 13];36(S2):104–10. Available from: https://www.liebertpub.com/doi/10.1089/apc.2022.0078

77. Chasco EE, Hoth AB, Cho H, Shafer C, Siegler AJ, Ohl ME. Mixed-Methods Evaluation of the Incorporation of Home Specimen Self-Collection Kits for Laboratory Testing in a Telehealth Program for HIV Pre-exposure Prophylaxis. AIDS Behav [Internet]. 2021 Aug 1 [cited 2024 Aug 13];25(8):2463–82. Available from: https://doi.org/10.1007/s10461-021-03209-9

78. Choko AT, Nanfuka M, Birungi J, Taasi G, Kisembo P, Helleringer S. A pilot trial of the peer-based distribution of HIV self-test kits among fishermen in Bulisa, Uganda. PLOS ONE [Internet]. 2018 Nov 29 [cited 2024 Aug 13];13(11):e0208191. Available from: https://journals.plos.org/plosone/article?id=10.1371/journal.pone.0208191

79. Chu VT, Schwartz NG, Donnelly MAP, Chuey MR, Soto R, Yousaf AR, et al. Comparison of Home Antigen Testing With RT-PCR and Viral Culture During the Course of SARS-CoV-2 Infection. JAMA Intern Med [Internet]. 2022 Jul 1 [cited 2024 Aug 13];182(7):701–9. Available from: https://doi.org/10.1001/jamainternmed.2022.1827

80. Dawkins M, Bishop L, Walker P, Otmaskin D, Ying J, Schmidt R, et al. Clinical Integration of a Highly Accurate Polymerase Chain Reaction Point-of-Care Test Can Inform Immediate Treatment Decisions for Chlamydia, Gonorrhea, and Trichomonas. Sex Transm Dis [Internet]. 2022 Apr [cited 2024 Aug 13];49(4):262. Available from: https://journals.lww.com/stdjournal/abstract/2022/04000/clinical_integration_of_a_highly_accurate.3.aspx

81. De Schacht C, Lucas C, Paulo P, Van Rompaey S, Fernando AN, Chinai JE, et al. Reaching Men and Young Adults in a Pharmacy-Based HIV Self-Testing Strategy: Results from an Acceptability

Study in Mozambique. AIDS Res Hum Retroviruses [Internet]. 2022 Aug [cited 2024 Aug 13];38(8):622–30. Available from: https://www.liebertpub.com/doi/10.1089/aid.2021.0116

82. Des Marais AC, Zhao Y, Hobbs MM, Barclay L, Brewer NT, Smith JS. Home Self-Collection by Mail to Test for Human Papillomavirus and Sexually Transmitted Infections. Obstet Gynecol [Internet]. 2018 Dec [cited 2024 Aug 28];132(6):1412–20. Available from: https://www.ncbi.nlm.nih.gov/pmc/articles/PMC6249061/

83. Dovel K, Shaba F, Offorjebe OA, Balakasi K, Nyirenda M, Phiri K, et al. Effect of facility-based HIV self-testing on uptake of testing among outpatients in Malawi: a cluster-randomised trial. Lancet Glob Health [Internet]. 2020 Feb 1 [cited 2024 Aug 13];8(2):e276–87. Available from: https://www.thelancet.com/journals/langlo/article/PIIS2214-109X(19)30534-0/fulltext

84. Dubé K, Agarwal H, Carter WB, Dee L, Taylor J, Roebuck C, et al. Participant experiences using novel home-based blood collection device for viral load testing in the HIV cure trials with analytical treatment interruptions. HIV Res Clin Pract [Internet]. 2022 Aug 2 [cited 2024 Aug 13];23(1):76–90. Available from: https://www.ncbi.nlm.nih.gov/pmc/articles/PMC9403870/

85. Edelstein ZR, Wahnich A, Purpura LJ, Salcuni PM, Tsoi BW, Kobrak PH, et al. Five Waves of an Online HIV Self-Test Giveaway in New York City, 2015 to 2018. Sex Transm Dis [Internet]. 2020 May [cited 2024 Aug 13];47(5S):S41. Available from: https://journals.lww.com/stdjournal/fulltext/2020/05001/five_waves_of_an_online_hiv_self_test_giveaway_in.9.aspx

86. Francis SC, Miiro G, Nakuya K, Rutakumwa R, Nakiyingi-Miiro J, Nabaggala G, et al. Self-Collection of Vaginal Swabs Among Adolescent Girls in a School-Setting in East Africa. Sex Transm Dis [Internet]. 2019 May [cited 2024 Aug 13];46(5):335. Available from: https://journals.lww.com/stdjournal/abstract/2019/05000/self_collection_of_vaginal_swabs_among_adolescent.10.aspx

87. Francis SC, Mthiyane TN, Baisley K, Mchunu SL, Ferguson JB, Smit T, et al. Prevalence of sexually transmitted infections among young people in South Africa: A nested survey in a health and demographic surveillance site. PLOS Med [Internet]. 2018 Feb 27 [cited 2024 Aug 13];15(2):e1002512. Available from: https://journals.plos.org/plosmedicine/article?id=10.1371/journal.pmed.1002512

88. Gaydos CA, Barnes M, Holden J, Silver B, Smith R, Hardick J, et al. Acceptability and feasibility of recruiting women to collect a self-administered vaginal swab at a pharmacy clinic for sexually transmissible infection screening. Sex Health [Internet]. 2020 Aug 24 [cited 2024 Aug 13];17(4):392–4. Available from: https://www.publish.csiro.au/sh/SH20077

89. González-Montalvo M del M, Dickson PF, Saber LB, Boehm RA, Phillips VL, Akiyama MJ, et al. Opinions of former jail residents about self-collection of SARS-CoV-2 specimens, paired with wastewater surveillance: A qualitative study rapidly examining acceptability of COVID-19 mitigation measures. PLOS ONE [Internet]. 2023 May 8 [cited 2024 Aug 13];18(5):e0285364. Available from: https://journals.plos.org/plosone/article?id=10.1371/journal.pone.0285364

90. Grésenguet G, Longo J de D, Tonen-Wolyec S, Bouassa RSM, Belec L. Acceptability and Usability Evaluation of Finger-Stick Whole Blood HIV Self-Test as An HIV Screening Tool Adapted to The General Public in The Central African Republic. Open AIDS J [Internet]. 2017 Nov 21 [cited 2024 Aug 13];11:101–18. Available from: https://www.ncbi.nlm.nih.gov/pmc/articles/PMC5730956/

91. Habel MA, Brookmeyer KA, Oliver-Veronesi R, Haffner MM. Creating Innovative Sexually Transmitted Infection Testing Options for University Students: The Impact of an STI Self-testing Program. Sex Transm Dis [Internet]. 2018 Apr [cited 2024 Aug 13];45(4):272. Available from: https://journals.lww.com/stdjournal/fulltext/2018/04000/creating_innovative_sexually_transmitted_infection.11.aspx

92. Haddad MS, Bifulco L, McIntosh J, Garcia MMC. Rectal specimen self-collection for chlamydia and gonorrhea screening: a cross-sectional feasibility study at a community health center. Pilot Feasibility Stud [Internet]. 2021 Nov 15 [cited 2024 Aug 13];7(1):208. Available from: https://doi.org/10.1186/s40814-021-00928-7

93. Harichund C, Kunene P, Moshabela M. Feasibility of HIV self-testing: experiences of people seeking HIV testing in rural and urban KwaZulu-Natal, South Africa. Afr J AIDS Res [Internet]. 2019 Apr 3 [cited 2024 Aug 13];18(2):115–22. Available from: https://doi.org/10.2989/16085906.2019.1621358

94. Harichund C, Karim QA, Kunene P, Simelane S, Moshabela M. Exploring factors that influence the integration of HIVST with HCT using a qualitative comparative cross-over design in KwaZulu-Natal, South Africa. Glob Public Health [Internet]. 2019 Sep 2 [cited 2024 Aug 14];14(9):1275–87. Available from: https://doi.org/10.1080/17441692.2019.1587638

95. Harichund C, Karim QA, Kunene P, Simelane S, Moshabela M. HIV self-testing as part of a differentiated HIV testing approach: exploring urban and rural adult experiences from KwaZulu-Natal, South Africa using a cross-over study design. BMC Public Health [Internet]. 2019 Jan 11 [cited 2024 Aug 14];19(1):53. Available from: https://doi.org/10.1186/s12889-018-6366-9

96. Hector J, Davies MA, Dekker-Boersema J, Aly MM, Abdalad CCA, Langa EBR, et al. Acceptability and performance of a directly assisted oral HIV self-testing intervention in adolescents in rural Mozambique. PLOS ONE [Internet]. 2018 Apr 5 [cited 2024 Aug 14];13(4):e0195391. Available from: https://journals.plos.org/plosone/article?id=10.1371/journal.pone.0195391

97. Herbert C, Broach J, Heetderks W, Qashu F, Gibson L, Pretz C, et al. Feasibility of At-Home Serial Testing Using Over-the-Counter SARS-CoV-2 Tests With a Digital Smartphone App for Assistance: Longitudinal Cohort Study. JMIR Form Res [Internet]. 2022 Oct 18 [cited 2024 Aug 14];6(10):e35426. Available from: https://formative.jmir.org/2022/10/e35426

98. Hergott DEB, Owalla TJ, Balkus JE, Apio B, Lema J, Cemeri B, et al. Feasibility of community at-home dried blood spot collection combined with pooled reverse transcription PCR as a viable and convenient method for malaria epidemiology studies. Malar J [Internet]. 2022 Jul 14 [cited 2024 Aug 14];21(1):221. Available from: https://doi.org/10.1186/s12936-022-04239-x

99. Hiransuthikul A, Janamnuaysook R, Himma L, Taya C, Amatsombat T, Chumnanwet P, et al. Acceptability and satisfaction towards self-collection for chlamydia and gonorrhoea testing among transgender women in Tangerine Clinic, Thailand: shifting towards the new normal. J Int AIDS Soc [Internet]. 2021 [cited 2024 Aug 14];24(9):e25801. Available from: https://onlinelibrary.wiley.com/doi/abs/10.1002/jia2.25801

100. Hoagland B, Torres TS, Bezerra DRB, Benedetti M, Pimenta C, Veloso VG, et al. High acceptability of PrEP teleconsultation and HIV self-testing among PrEP users during the COVID-19 pandemic in Brazil. Braz J Infect Dis [Internet]. 2021 Jan 1 [cited 2024 Aug 14];25(1):101037. Available from: https://www.sciencedirect.com/science/article/pii/S1413867020301641

101. Hogenson E, Jett-Goheen M, Gaydos CA. An Analysis of User Survey Data for an Internet Program for Testing for Sexually Transmitted Infections, I Want the Kit, in Maryland and Washington, DC. Sex Transm Dis [Internet]. 2019 Dec [cited 2024 Aug 14];46(12):768. Available from: https://journals.lww.com/stdjournal/abstract/2019/12000/an_analysis_of_user_survey_data_for_an_internet.3.aspx

102. Horvath KJ, Bwanika JM, Lammert S, Banonya J, Atuhaire J, Banturaki G, et al. HiSTEP: A Single-Arm Pilot Study of a Technology-Assisted HIV Self-testing Intervention in Kampala, Uganda. AIDS Behav [Internet]. 2022 Mar 1 [cited 2024 Aug 14];26(3):935–46. Available from: https://doi.org/10.1007/s10461-021-03449-9

103. Hsieh YH, Lewis MK, Viertel VG, Myer D, Rothman RE, Gaydos CA. Performance evaluation and acceptability of point-of-care Trichomonas vaginalis testing in adult female emergency department patients. Int J STD AIDS [Internet]. 2020 Dec 1 [cited 2024 Aug 14];31(14):1364–72. Available from: https://doi.org/10.1177/0956462420956532

104. Huang E, Marlin RW, Young SD, Medline A, Klausner JD. https://doi.org/10.1521/aeap.2016.28.4.341. Guilford; 2016 [cited 2024 Aug 14]. Using Grindr, a Smartphone Social-Networking Application, to Increase HIV Self-Testing Among Black and Latino Men Who Have Sex With Men in Los Angeles, 2014. Available from: https://guilfordjournals.com/doi/10.1521/aeap.2016.28.4.341

105. Hubbard SJ, Ma M, Wahnich A, Clarke A, Myers JE, Saleh LD. #Testathome: Implementing 2 Phases of a HIV Self-Testing Program Through Community-Based Organization Partnerships in New York City. Sex Transm Dis [Internet]. 2020 May [cited 2024 Aug 14];47(5S):S48. Available from: https://journals.lww.com/stdjournal/fulltext/2020/05001/_testathome__implementing_2_phases_of_a_hiv.10.aspx

106. Isabel S, Cohen-Silver J, Jung H, Tam B, Lota M, Sivilotti M, et al. Swish and gargle saliva sampling is a patient-friendly and comparable alternative to nasopharyngeal swabs to detect SARS-CoV-2 in outpatient settings for adults and children. Microbiol Spectr [Internet]. 2023 Oct 20 [cited 2024 May 22];11(6):e01723-23. Available from: https://journals.asm.org/doi/full/10.1128/spectrum.01723-23

107. Ivanova Reipold E, Fajardo E, Juma E, Bukusi D, Bermudez Aza E, Jamil MS, et al. Usability and acceptability of oral fluid hepatitis C self-testing among people who inject drugs in Coastal Kenya: a cross-sectional pilot study. BMC Infect Dis [Internet]. 2022 Sep 15 [cited 2024 Jun 10];22(1):738. Available from: https://bmcinfectdis.biomedcentral.com/articles/10.1186/s12879-022-07712-9

108. John SA, Cain D, Bradford-Rogers J, Rendina HJ, Grov C. Gay and Bisexual Men's Experiences Using Self-Testing Kits for HIV and Rectal and Urethral Bacterial Sexually Transmitted Infections: Lessons Learned from a Study With Home-Based Testing. Int J Sex Health [Internet]. 2019 Jul 3 [cited 2024 Aug 20];31(3):308–18. Available from: https://doi.org/10.1080/19317611.2019.1645075

109. Johnson C, Kumwenda M, Meghji J, Choko AT, Phiri M, Hatzold K, et al. 'Too old to test?': A life course approach to HIV-related risk and self-testing among midlife-older adults in Malawi. BMC Public Health [Internet]. 2021 Apr 3 [cited 2024 Aug 14];21(1):650. Available from: https://doi.org/10.1186/s12889-021-10573-7

110. Jolliff A, Werner NE, Barton HJ, Howell KD, Kelly MM, Morgen M, et al. Caregiver perceptions of in-home COVID-19 testing for children with medical complexity: a qualitative study. BMC Pediatr [Internet]. 2022 Sep 8 [cited 2024 Aug 14];22(1):533. Available from: https://doi.org/10.1186/s12887-022-03550-5

111. Matovu JBJ, Mayega RW, Nalubega S, Byakika-Tusiime J. Acceptability, Willingness to use and Preferred Distribution Models of Oral- Based HIV Self-Testing Kits among Key and Priority Populations Enrolled in HIV Pre-Exposure Prophylaxis Clinics in Central Uganda. A Mixed-Methods Cross-Sectional Study. Arch Clin Biomed Res [Internet]. 2022 Nov 22 [cited 2024 Aug 14];6(6):933–43. Available from: http://www.fotunejournals.com/acceptability-willingness-to-use-and-preferred-distribution-models-of-oral-based-hiv-selftesting-kits-among-key-and-priority-popul.html

112. Kra AK, Fotso AS, N'guessan KN, Geoffroy O, Younoussa S, Kabemba OK, et al. Can HIV self-testing reach first-time testers? A telephone survey among self-test end users in Côte d'Ivoire, Mali, and Senegal. BMC Infect Dis [Internet]. 2023 Sep 25 [cited 2024 Aug 14];22(1):972. Available from: https://doi.org/10.1186/s12879-023-08626-w

113. Kumwenda MK, Mavhu W, Lora WS, Chilongosi R, Sikwese S, Taegtmeyer M, et al. Feasibility and acceptability of a peer-led HIV self-testing model among female sex workers in Malawi: a qualitative study. BMJ Open [Internet]. 2021 Dec 1 [cited 2024 Aug 14];11(12):e049248. Available from: https://bmjopen.bmj.com/content/11/12/e049248

114. Kurth AE, Cleland CM, Chhun N, Sidle JE, Were E, Naanyu V, et al. Accuracy and Acceptability of Oral Fluid HIV Self-Testing in a General Adult Population in Kenya. AIDS Behav [Internet]. 2016 Apr 1 [cited 2024 Aug 14];20(4):870–9. Available from: https://doi.org/10.1007/s10461-015-1213-9

115. Ky-Zerbo O, Desclaux A, Boye S, Vautier A, Rouveau N, Kouadio BA, et al. Willingness to use and distribute HIV self-test kits to clients and partners: A qualitative analysis of female sex workers' collective opinion and attitude in Côte d'Ivoire, Mali, and Senegal. Womens Health [Internet]. 2022 Jan 1 [cited 2024 Aug 14];18:17455057221092268. Available from: https://doi.org/10.1177/17455057221092268

116. Ky-Zerbo O, Desclaux A, Boye S, Maheu-Giroux M, Rouveau N, Vautier A, et al. "I take it and give it to my partners who will give it to their partners": Secondary distribution of HIV self-tests by key populations in Côte d'Ivoire, Mali, and Senegal. BMC Infect Dis [Internet]. 2023 May 24 [cited 2024 Aug 20];22(1):970. Available from: https://doi.org/10.1186/s12879-023-08319-4

117. Lapsley R, Beima-Sofie K, Moraa H, Manyeki V, Mung'ala C, Kohler PK, et al. "They have given you the morale and confidence:" Adolescents and young adults want more community-based oral HIV self-testing options in Kenya. AIDS Care [Internet]. 2023 Mar [cited 2024 Aug 20];35(3):392–8. Available from: https://www.ncbi.nlm.nih.gov/pmc/articles/PMC9592677/

118. Lee GYL, Lim RBT. Are self-test kits still relevant post COVID-19 pandemic? Qualitative study on working adults' perceptions. Infect Dis Health [Internet]. 2024 May 1 [cited 2024 Aug 20];29(2):73–80. Available from: https://www.sciencedirect.com/science/article/pii/S2468045123001116

119. Lee RE, Todd M, Oh H, Han S, Santana M, Aguilar-Troncoso J, et al. Acceptability and Feasibility of Saliva-delivered PCR Coronavirus 2019 Tests for Young Children. Pediatrics [Internet]. 2023

Jul [cited 2024 Aug 20];152(Suppl 1):e2022060352D. Available from: https://www.ncbi.nlm.nih.gov/pmc/articles/PMC10312283/

120. LeRouge C, Durneva P, Lyon V, Thompson M. Health Consumer Engagement, Enablement, and Empowerment in Smartphone-Enabled Home-Based Diagnostic Testing for Viral Infections: Mixed Methods Study. JMIR MHealth UHealth [Internet]. 2022 Jun 30 [cited 2024 Aug 20];10(6):e34685. Available from: https://mhealth.jmir.org/2022/6/e34685

121. Lippman SA, Lane T, Rabede O, Gilmore H, Chen YH, Mlotshwa N, et al. High Acceptability and Increased HIV-Testing Frequency After Introduction of HIV Self-Testing and Network Distribution Among South African MSM. JAIDS J Acquir Immune Defic Syndr [Internet]. 2018 Mar 1 [cited 2024 Aug 20];77(3):279. Available from: https://journals.lww.com/jaids/abstract/2018/03010/high_acceptability_and_increased_hiv_testing.7.aspx

122. Lippman SA, Moran L, Sevelius J, Castillo LS, Ventura A, Treves-Kagan S, et al. Acceptability and Feasibility of HIV Self-Testing Among Transgender Women in San Francisco: A Mixed Methods Pilot Study. AIDS Behav [Internet]. 2016 Apr 1 [cited 2024 Aug 20];20(4):928–38. Available from: https://doi.org/10.1007/s10461-015-1236-2

123. Liu M, Zhu Y, Gao H, Li J. Examining Chinese Users' Feedback Comments on HIV Self-testing Kits From e-Commerce Platforms: Thematic and Content Analysis. J Med Internet Res [Internet]. 2022 Nov 30 [cited 2024 Aug 20];24(11):e38398. Available from: https://www.jmir.org/2022/11/e38398

124. Lora WS, Desmond N, Obasi A, Kumwenda M, Taegtmeyer M, Tolhurst R, et al. "I wanted evidence that my status had changed, so that is why I tested": experiences with HIV self-testing among female sex workers in Malawi. AIDS Care [Internet]. 2020 May 13 [cited 2024 Aug 20];32(sup2):206–13. Available from: https://doi.org/10.1080/09540121.2020.1739212

125. Luisi N. Use of COVIDTests.gov At-Home Test Kits Among Adults in a National Household Probability Sample — United States, 2022. MMWR Morb Mortal Wkly Rep [Internet]. 2023 [cited 2024 Aug 20];72. Available from: https://www.cdc.gov/mmwr/volumes/72/wr/mm7216a6.htm

126. Lyons CE, Coly K, Bowring AL, Liestman B, Diouf D, Wong VJ, et al. Use and Acceptability of HIV Self-Testing Among First-Time Testers at Risk for HIV in Senegal. AIDS Behav [Internet]. 2019 Sep 1 [cited 2024 Aug 20];23(2):130–41. Available from: https://doi.org/10.1007/s10461-019-02552-2

127. Mabonga E, Manabe YC, Elbireer A, Mbazira JK, Nabaggala MS, Kiragga A, et al. Prevalence and predictors of asymptomatic Chlamydia trachomatis and Neisseria gonorrhoeae in a Ugandan population most at risk of HIV transmission. Int J STD AIDS [Internet]. 2021 May 1 [cited 2024 Aug 20];32(6):510–6. Available from: https://doi.org/10.1177/0956462420979799

128. MacGowan RJ, Chavez PR, Gravens L, Wesolowski LG, Sharma A, McNaghten AD, et al. Pilot Evaluation of the Ability of Men Who Have Sex with Men to Self-Administer Rapid HIV Tests, Prepare Dried Blood Spot Cards, and Interpret Test Results, Atlanta, Georgia, 2013. AIDS Behav [Internet]. 2018 Jan 1 [cited 2024 Aug 20];22(1):117–26. Available from: https://doi.org/10.1007/s10461-017-1932-1

129. Majam M, Fischer A, Ivanova Reipold E, Rhagnath N, Msolomba V, Lalla-Edward ST. A Lay-User Assessment of Hepatitis C Virus Self-Testing Device Usability and Interpretation in

Johannesburg, South Africa. Diagnostics [Internet]. 2021 Mar [cited 2024 Aug 25];11(3):463. Available from: https://www.mdpi.com/2075-4418/11/3/463

130. Majam M, Mazzola L, Rhagnath N, Lalla-Edward ST, Mahomed R, Venter WDF, et al. Usability assessment of seven HIV self-test devices conducted with lay-users in Johannesburg, South Africa. PLOS ONE [Internet]. 2020 Jan 14 [cited 2024 Feb 26];15(1):e0227198. Available from: https://journals.plos.org/plosone/article?id=10.1371/journal.pone.0227198

131. Marley G, Kang D, Wilson EC, Huang T, Qian Y, Li X, et al. Introducing rapid oral–fluid HIV testing among high risk populations in Shandong, China: feasibility and challenges. BMC Public Health [Internet]. 2014 May 3 [cited 2024 Aug 20];14(1):422. Available from: https://doi.org/10.1186/1471-2458-14-422

132. Marley G, Fu G, Zhang Y, Li J, Tucker JD, Tang W, et al. Willingness of Chinese Men Who Have Sex With Men to Use Smartphone-Based Electronic Readers for HIV Self-testing: Web-Based Cross-sectional Study. J Med Internet Res [Internet]. 2021 Nov 19 [cited 2024 Aug 20];23(11):e26480. Available from: https://www.jmir.org/2021/11/e26480

133. Martínez-Pérez GZ, Shilton S, Salazar PSM, Oses PP, Torres-Slimming PA, Batheja D, et al. SARS-CoV-2 self-testing in Peru: a cross-sectional survey of values and attitudes of the general population. BMJ Open [Internet]. 2023 Jul 1 [cited 2024 Aug 20];13(7):e068980. Available from: https://bmjopen.bmj.com/content/13/7/e068980

134. Mavhu W, Makamba M, Hatzold K, Maringwa G, Takaruza A, Mutseta M, et al. Preferences for oral-fluid-based or blood-based HIV self-testing and provider-delivered testing: an observational study among different populations in Zimbabwe. BMC Infect Dis [Internet]. 2023 Oct 17 [cited 2024 Aug 25];22(1):973. Available from: https://doi.org/10.1186/s12879-023-08624-y

135. McMahon SA, Musoke DK, Wachinger J, Nakitende A, Amongin J, Nanyiri E, et al. Unintended uses, meanings, and consequences: HIV self-testing among female sex workers in urban Uganda. AIDS Care [Internet]. 2021 Oct 3 [cited 2024 Aug 20];33(10):1278–85. Available from: https://doi.org/10.1080/09540121.2020.1837722

136. Melnyk AI, Toal C, Glass Clark S, Bradley M. Home urinary tract infection testing: patient experience and satisfaction with polymerase chain reaction kit. Int Urogynecology J [Internet]. 2023 May 1 [cited 2024 Aug 20];34(5):1055–60. Available from: https://doi.org/10.1007/s00192-022-05309-z

137. Mkopi A, Korte JE, Lesslie V, diNapoli M, Mutiso F, Mwajubwa S, et al. Acceptability and uptake of oral HIV self-testing among rural community members in Tanzania: a pilot study. AIDS Care [Internet]. 2023 Sep 2 [cited 2024 Aug 20];35(9):1338–45. Available from: https://doi.org/10.1080/09540121.2023.2217376

138. Moradi G, Amini EE, Valipour A, Tayeri K, Kazerooni PA, Molaeipour L, et al. The study of feasibility and acceptability of using HIV self-tests in high-risk Iranian populations (FSWs, MSM, and TGs): a cross-sectional study. Harm Reduct J [Internet]. 2022 Jun 3 [cited 2024 Aug 20];19(1):61. Available from: https://doi.org/10.1186/s12954-022-00641-5

139. Mphande M, Campbell P, Hoffman RM, Phiri K, Nyirenda M, Gupta SK, et al. Barriers and facilitators to facility HIV self-testing in outpatient settings in Malawi: a qualitative study. BMC Public Health [Internet]. 2021 Dec 2 [cited 2024 Aug 20];21(1):2200. Available from: https://doi.org/10.1186/s12889-021-12213-6

140. Mujugira A, Nakyanzi A, Kasiita V, Kamusiime B, Nalukwago GK, Nalumansi A, et al. HIV self-testing and oral pre-exposure prophylaxis are empowering for sex workers and their intimate partners: a qualitative study in Uganda. J Int AIDS Soc [Internet]. 2021 [cited 2024 Aug 20];24(9):e25782. Available from: https://onlinelibrary.wiley.com/doi/abs/10.1002/jia2.25782

141. Mukora-Mutseyekwa F, Mundagowa PT, Kangwende RA, Murapa T, Tirivavi M, Mukuwapasi W, et al. Implementation of a campus-based and peer-delivered HIV self-testing intervention to improve the uptake of HIV testing services among university students in Zimbabwe: the SAYS initiative. BMC Health Serv Res [Internet]. 2022 Feb 18 [cited 2024 Aug 20];22(1):222. Available from: https://doi.org/10.1186/s12913-022-07622-1

142. Nambusi K, Gbolahan A, Maureen SM, Tsholofelo RT, Motswedi AM, Simani G, et al. Youth and healthcare workers' perspectives on the feasibility and acceptability of self-testing for HIV, Hepatitis and Syphilis among young people: Qualitative findings from a pilot study in Gaborone, Botswana. PLOS ONE [Internet]. 2023 Jul 20 [cited 2024 Aug 20];18(7):e0288971. Available from: https://journals.plos.org/plosone/article?id=10.1371/journal.pone.0288971

143. Nangendo J, Katahoire AR, Karamagi CA, Obeng-Amoako GO, Muwema M, Okiring J, et al. Uptake and perceptions of oral HIV self-testing delivered by village health teams among men in Central Uganda: A concurrent parallel mixed methods analysis. PLOS Glob Public Health [Internet]. 2023 Jun 14 [cited 2024 Aug 20];3(6):e0002019. Available from: https://journals.plos.org/globalpublichealth/article?id=10.1371/journal.pgph.0002019

144. Nelson JA, Zha P, Halawani M, Jones V. Evidence-Based Interventions Implemented into HIV Primary Care Clinics to Make Sexually Transmitted Infection Screening and Testing Routine: Outcomes of a Multi-Site Study. AIDS Patient Care STDs [Internet]. 2022 Nov [cited 2024 Aug 20];36(S2):92–103. Available from: https://www.liebertpub.com/doi/10.1089/apc.2022.0131

145. Ngoc BV, Majam M, Green K, Tran T, Hung MT, Que AL, et al. Acceptability, feasibility, and accuracy of blood-based HIV self-testing: A cross-sectional study in Ho Chi Minh City, Vietnam. PLOS Glob Public Health [Internet]. 2023 Feb 1 [cited 2024 Aug 20];3(2):e0001438. Available from: https://journals.plos.org/globalpublichealth/article?id=10.1371/journal.pgph.0001438

146. Ngure K, Heffron R, Mugo N, Thomson KA, Irungu E, Njuguna N, et al. Feasibility and acceptability of HIV self-testing among pre-exposure prophylaxis users in Kenya. J Int AIDS Soc [Internet]. 2017 [cited 2024 Aug 20];20(1):21234. Available from: https://onlinelibrary.wiley.com/doi/abs/10.7448/IAS.20.1.21234

147. Nguyen TT, Zeger WG, Wadman MC, Barksdale AN. Accuracy and Acceptance of a Self-Collection Model for Respiratory Tract Infection Diagnostics: A Concise Clinical Literature Review. J Emerg Nurs [Internet]. 2021 Sep 1 [cited 2024 Feb 21];47(5):798–806. Available from: https://www.sciencedirect.com/science/article/pii/S0099176721001094

148. O'Byrne P, Musten A, Ho N. HIV self-testing in the real world is acceptable for many: post-test participant feedback from the GetaKit study in Ottawa, Canada. J Res Nurs [Internet]. 2022 Dec 1 [cited 2024 Aug 20];27(8):757–64. Available from: https://doi.org/10.1177/17449871221137761

149. Ogale Y, Yeh PT, Kennedy CE, Toskin I, Narasimhan M. Self-collection of samples as an additional approach to deliver testing services for sexually transmitted infections: a systematic review and meta-analysis. BMJ Glob Health [Internet]. 2019 Apr 1 [cited 2024 Mar 1];4(2):e001349. Available from: https://gh.bmj.com/content/4/2/e001349

150. Oyaro P, Kwena Z, Bukusi EA, Baeten JM. Is HIV Self-Testing a Strategy to Increase Repeat Testing Among Pregnant and Postpartum Women? A Pilot Mixed Methods Study. JAIDS J Acquir Immune Defic Syndr [Internet]. 2020 Aug 1 [cited 2024 Aug 21];84(4):365. Available from: https://journals.lww.com/jaids/abstract/2020/08010/is_hiv_self_testing_a_strategy_to_increase_repeat.5.aspx

151. Pant Pai N, Bhargava M, Joseph L, Sharma J, Pillay S, Balram B, et al. Will an Unsupervised Self-Testing Strategy Be Feasible to Operationalize in Canada? Results from a Pilot Study in Students of a Large Canadian University. AIDS Res Treat [Internet]. 2014 [cited 2024 Aug 21];2014(1):747619. Available from: https://onlinelibrary.wiley.com/doi/abs/10.1155/2014/747619

152. Patel AV, Abrams SM, Gaydos CA, Jett-Goheen M, Latkin CA, Rothman RE, et al. Increasing HIV testing engagement through provision of home HIV self-testing kits for patients who decline testing in the emergency department: a pilot randomisation study. Sex Transm Infect [Internet]. 2019 Aug [cited 2024 Aug 22];95(5):358–60. Available from: https://www.ncbi.nlm.nih.gov/pmc/articles/PMC6294698/

153. Sri-Pathmanathan C, Nhamo D, Mamvuto T, Chapwanya G, Terris-Prestholt F, Mahaka I, et al. Syphilis self-testing to expand test uptake among men who have sex with men: a theoretically informed mixed methods study in Zimbabwe. Sex Transm Infect [Internet]. 2022 May 1 [cited 2024 Aug 22];98(3):197–202. Available from: https://sti.bmj.com/content/98/3/197

154. Peck RB, Lim JM, van Rooyen H, Mukoma W, Chepuka L, Bansil P, et al. What Should the Ideal HIV Self-Test Look Like? A Usability Study of Test Prototypes in Unsupervised HIV Self-Testing in Kenya, Malawi, and South Africa. AIDS Behav [Internet]. 2014 Jul 1 [cited 2024 Aug 22];18(4):422–32. Available from: https://doi.org/10.1007/s10461-014-0818-8

155. Phanuphak N, Anand T, Jantarapakde J, Nitpolprasert C, Himmad K, Sungsing T, et al. What would you choose: Online or Offline or Mixed services? Feasibility of online HIV counselling and testing among Thai men who have sex with men and transgender women and factors associated with service uptake. J Int AIDS Soc [Internet]. 2018 [cited 2024 Aug 22];21(S5):e25118. Available from: https://onlinelibrary.wiley.com/doi/abs/10.1002/jia2.25118

156. Phongphiew P, Songtaweesin WN, Paiboon N, Phiphatkhunarnon P, Srimuan P, Sowaprux T, et al. Acceptability of blood-based HIV self-testing among adolescents aged 15–19 years at risk of HIV acquisition in Bangkok. Int J STD AIDS [Internet]. 2021 Sep 1 [cited 2024 Aug 22];32(10):927–32. Available from: https://doi.org/10.1177/09564624211003742

157. Pierre G, Umutoni A, Dzinamarira T. A qualitative study on oral-fluid-based HIV self-testing experiences among men in Kigali, Rwanda. Pan Afr Med J [Internet]. 2020 Oct 8 [cited 2024 Aug 22];37(138). Available from: https://www.panafrican-med-journal.com//content/article/37/138/full

158. Rael CT, Kutner BA, Lopez-Rios J, Lentz C, Dolezal C, Balán IC. Experiences of Transgender Women Who Used a Dual HIV/Syphilis Rapid Self-test to Screen Themselves and Potential Sexual Partners (the SMARTtest Study). AIDS Behav [Internet]. 2022 Apr 1 [cited 2024 Aug 22];26(4):1229–37. Available from: https://doi.org/10.1007/s10461-021-03478-4

159. Rao A, Patil S, Nirmalkar A, Bagul R, Ghule U, Panchal N, et al. HIV oral self-screening test among HIV/STD/TB clinic attendees: A mixed-method pilot investigation examining merit for larger evaluation. Indian J Med Res [Internet]. 2022 Apr [cited 2024 Aug 22];155(3 & 4):403. Available from: https://journals.lww.com/ijmr/fulltext/2022/03000/hiv_oral_self_screening_test_among_hiv_std_tb.12.aspx

160. Reipold EI, Farahat A, Elbeeh A, Soliman R, Aza EB, Jamil MS, et al. Usability and acceptability of self-testing for hepatitis C virus infection among the general population in the Nile Delta region of Egypt. BMC Public Health [Internet]. 2021 Jun 22 [cited 2024 Aug 22];21(1):1188. Available from: https://doi.org/10.1186/s12889-021-11169-x

161. Long RX, You WZ, Dong MG, Jennifer M, Ming RK, Yan Z. Uptake of HIV Self-testing among Men Who have Sex with Men in Beijing, China:a Cross-sectional Study. Biomed Environ Sci [Internet]. 2017 Jun 1 [cited 2024 Aug 22];30(6):407–17. Available from: https://www.besjournal.com/en/article/doi/10.3967/bes2017.054

162. Rick F, Ishigami BI, Figueiroa FJ, Cahete LR de C, Humar RCH, Jesus R de, et al. Impact of COVID-19 on income, prevention attitudes, and access to healthcare among male clients in a Sexually Transmitted Infections clinic. Braz J Infect Dis [Internet]. 2021 Sep 1 [cited 2024 Aug 22];25(5):101617. Available from: https://www.sciencedirect.com/science/article/pii/S1413867021000866

163. Ritchwood TD, Selin A, Pettifor A, Lippman SA, Gilmore H, Kimaru L, et al. HIV self-testing: South African young adults' recommendations for ease of use, test kit contents, accessibility, and supportive resources. BMC Public Health [Internet]. 2019 Jan 29 [cited 2024 Aug 23];19(1):123. Available from: https://doi.org/10.1186/s12889-019-6402-4

164. Rocha DA, Azevedo MJN, Batista SJS, Beltrão ÊS, Moraes CO, Araújo AF, et al. T. vaginalis in riverside women in Amazonia, Brazil: an experience using the EVALYN® BRUSH vaginal self-collection device. J Infect Dev Ctries [Internet]. 2019 Nov 30 [cited 2024 Aug 23];13(11):1029–37. Available from: https://jidc.org/index.php/journal/article/view/32087075

165. Rochford H, Larsson L, Simms V, Mavodza C, Sigwadhi L, Dauya E, et al. Acceptability of self-collected vaginal swabs for sexually transmitted infection testing among youth in a community-based setting in Zimbabwe. Int J STD AIDS [Internet]. 2023 Jul 1 [cited 2024 Aug 23];34(8):574–80. Available from: https://doi.org/10.1177/09564624231152804

166. Wellcome Open Research | Open Access Publishing Platform [Internet]. 2020 [cited 2024 Aug 23]. Acceptability and feasibility of genital self-sampling for the ... Available from: https://wellcomeopenresearch.org/articles/5-61

167. Saberi P, Ming K, Shrestha I, Scott H, Thorson B, Liu A. Feasibility and Acceptability of Home-Collected Samples for Human Immunodeficiency Virus Preexposure Prophylaxis and Severe Acute Respiratory Syndrome Coronavirus 2 Laboratory Tests in San Francisco Primary Care Clinics. Open Forum Infect Dis [Internet]. 2022 Feb 1 [cited 2024 Aug 23];9(2):ofab657. Available from: https://doi.org/10.1093/ofid/ofab657

168. Sarkar A, Mburu G, Shivkumar PV, Sharma P, Campbell F, Behera J, et al. Feasibility of supervised self-testing using an oral fluid-based HIV rapid testing method: a cross-sectional, mixed method study among pregnant women in rural India. J Int AIDS Soc [Internet]. 2016

[cited 2024 Aug 23];19(1):20993. Available from: https://onlinelibrary.wiley.com/doi/abs/10.7448/IAS.19.1.20993

169. Schilling J, Moeller FG, Peterson R, Beltz B, Joshi D, Gartner D, et al. Testing the Acceptability and Usability of an AI-Enabled COVID-19 Diagnostic Tool Among Diverse Adult Populations in the United States. Qual Manag Healthc [Internet]. 2023 Mar [cited 2024 Aug 23];32(Supplement 1):S35. Available from: https://journals.lww.com/qmhcjournal/fulltext/2023/01001/testing_the_acceptability_and_usability_of_an.6.aspx

170. Schnall R, John RM, Carballo-Dieguez A. Do High-Risk Young Adults Use the HIV Self-Test Appropriately? Observations from a Think-Aloud Study. AIDS Behav [Internet]. 2016 Apr 1 [cited 2024 Aug 23];20(4):939–48. Available from: https://doi.org/10.1007/s10461-015-1240-6

171. Schuster JE, Potts J, Selvarangan R, Mast DK, Goldman JL, for the School TLC Study Group. A COVID-19 Testing Preference Study in Schools. Pediatrics [Internet]. 2023 Jul 1 [cited 2024 Aug 23];152(Supplement 1):e2022060352H. Available from: https://doi.org/10.1542/peds.2022-060352H

172. Sharma A, Gandhi M, Sallabank G, Merrill L, Stephenson R. Perceptions and Experiences of Returning Self-collected Specimens for HIV, Bacterial STI and Potential PrEP Adherence Testing among Sexual Minority Men in the United States. AIDS Behav [Internet]. 2023 [cited 2024 Aug 23];27(4):1091–105. Available from: https://www.ncbi.nlm.nih.gov/pmc/articles/PMC9466335/

173. Sievers BL, Klotzle J, Khan TV. Evaluation of feasibility and user acceptance of lateral-flow self-testing for viral illness in a residential treatment rehabilitation facility. Health Justice [Internet]. 2022 Feb 26 [cited 2024 Aug 23];10(1):10. Available from: https://doi.org/10.1186/s40352-022-00173-x

174. Smith KS, Kaldor JM, Hocking JS, Jamil MS, McNulty AM, Read P, et al. The acceptability and cost of a home-based chlamydia retesting strategy: findings from the REACT randomised controlled trial. BMC Public Health [Internet]. 2016 Jan 28 [cited 2024 Aug 23];16(1):83. Available from: https://doi.org/10.1186/s12889-016-2727-4

175. Smith P, Wallace M, Bekker LG. Adolescents' experience of a rapid HIV self-testing device in youth-friendly clinic settings in Cape Town South Africa: a cross-sectional community based usability study. J Int AIDS Soc [Internet]. 2016 [cited 2024 Aug 23];19(1):21111. Available from: https://onlinelibrary.wiley.com/doi/abs/10.7448/IAS.19.1.21111

176. Soto R, Paul L, Porucznik CA, Xie H, Stinnett RC, Briggs B, et al. Effectiveness of Self-Collected, Ambient Temperature–Preserved Nasal Swabs Compared to Samples Collected by Trained Staff for Genotyping of Respiratory Viruses by Shotgun RNA Sequencing: Comparative Study. JMIR Form Res [Internet]. 2023 Nov 24 [cited 2024 Aug 23];7(1):e32848. Available from: https://formative.jmir.org/2023/1/e32848

177. Strong LE, Middendorf I, Turner M, V DKE, Sama V, Mou J, et al. Usability of an At-Home Anterior Nares SARS-CoV-2 RT-PCR Sample Collection Kit: Human Factors Feasibility Study. JMIR Hum Factors [Internet]. 2021 Dec 14 [cited 2024 Aug 23];8(4):e29234. Available from: https://humanfactors.jmir.org/2021/4/e29234

178. Sullivan SP, Sullivan PS, Stephenson R. Acceptability and Feasibility of a Telehealth Intervention for STI Testing Among Male Couples. AIDS Behav [Internet]. 2021 Dec 1 [cited 2024 Aug 23];25(12):4029–43. Available from: https://doi.org/10.1007/s10461-021-03173-4

179. Tonen-Wolyec S, Batina-Agasa S, Muwonga J, Bouassa RSM, Tshilumba CK, Bélec L. GS-IN Acceptability, feasibility, and individual preferences of blood-based HIV self-testing in a population-based sample of adolescents in Kisangani, Democratic Republic of the Congo. PLOS ONE [Internet]. 2019 Jul 1 [cited 2024 Feb 21];14(7):e0218795. Available from: https://journals.plos.org/plosone/article?id=10.1371/journal.pone.0218795

180. Tonen-Wolyec S, Sarassoro A, Muwonga Masidi J, Twite Banza E, Nsiku Dikumbwa G, Maseke Matondo DM, et al. Field evaluation of capillary blood and oral-fluid HIV self-tests in the Democratic Republic of the Congo. PLoS ONE [Internet]. 2020 Oct 5 [cited 2024 Aug 23];15(10):e0239607. Available from: https://www.ncbi.nlm.nih.gov/pmc/articles/PMC7535027/

181. Tonen-Wolyec S, Djang'eing'a RM, Batina-Agasa S, Tshilumba CK, Masidi JM, Hayette MP, et al. Self-testing for HIV, HBV, and HCV using finger-stick whole-blood multiplex immunochromatographic rapid test: A pilot feasibility study in sub-Saharan Africa. PLOS ONE [Internet]. 2021 Apr 9 [cited 2024 Aug 23];16(4):e0249701. Available from: https://journals.plos.org/plosone/article?id=10.1371/journal.pone.0249701

182. Tun W, Vu L, Dirisu O, Sekoni A, Shoyemi E, Njab J, et al. Uptake of HIV self-testing and linkage to treatment among men who have sex with men (MSM) in Nigeria: A pilot programme using key opinion leaders to reach MSM. J Int AIDS Soc [Internet]. 2018 [cited 2024 Aug 25];21(S5):e25124. Available from: https://onlinelibrary.wiley.com/doi/abs/10.1002/jia2.25124

183. Ursini T, Scarso S, Mugassa S, Othman JB, Yussuph AJ, Ndaboine E, et al. Assessing the prevalence of Female Genital Schistosomiasis and comparing the acceptability and performance of health worker-collected and self-collected cervical-vaginal swabs using PCR testing among women in North-Western Tanzania: The ShWAB study. PLoS Negl Trop Dis [Internet]. 2023 Jul 6 [cited 2024 Aug 25];17(7):e0011465. Available from: https://journals.plos.org/plosntds/article?id=10.1371/journal.pntd.0011465

184. Valentine-Graves M, Hall E, Guest JL, Adam E, Valencia R, Shinn K, et al. At-home self-collection of saliva, oropharyngeal swabs and dried blood spots for SARS-CoV-2 diagnosis and serology: Post-collection acceptability of specimen collection process and patient confidence in specimens. PLOS ONE [Internet]. 2020 Aug 5 [cited 2024 Feb 21];15(8):e0236775. Available from: https://journals.plos.org/plosone/article?id=10.1371/journal.pone.0236775

185. Volk JE, Lippman SA, Grinsztejn B, Lama JR, Fernandes NM, Gonzales P, et al. Acceptability and feasibility of HIV self-testing among men who have sex with men in Peru and Brazil. Int J STD AIDS [Internet]. 2016 Jun 1 [cited 2024 Aug 25];27(7):531–6. Available from: https://doi.org/10.1177/0956462415586676

186. Wachinger J, Kibuuka Musoke D, Oldenburg CE, Bärnighausen T, Ortblad KF, McMahon SA. "But I Gathered My Courage": HIV Self-Testing as a Pathway of Empowerment Among Ugandan Female Sex Workers. Qual Health Res [Internet]. 2021 Feb 1 [cited 2024 Aug 25];31(3):443–57. Available from: https://doi.org/10.1177/1049732320978392

187. Wang X, Tang Z, Wu Z, Nong Q, Li Y. Promoting oral HIV self-testing via the internet among men who have sex with men in China: a feasibility assessment. HIV Med [Internet]. 2020 [cited 2024 Aug 25];21(5):322–33. Available from: https://onlinelibrary.wiley.com/doi/abs/10.1111/hiv.12830

188. Ware NC, Wyatt MA, Pisarski EE, Kamusiime B, Kasiita V, Nalukwago G, et al. How pregnant women living with HIV and their male partners manage men's HIV self-testing: qualitative analysis of an HIVST secondary distribution process in Kampala, Uganda. J Int AIDS Soc [Internet]. 2023 [cited 2024 Aug 25];26(1):e26050. Available from: https://onlinelibrary.wiley.com/doi/abs/10.1002/jia2.26050

189. Wilson KS, Mugo C, Katz DA, Manyeki V, Mungwala C, Otiso L, et al. High Acceptance and Completion of HIV Self-testing Among Diverse Populations of Young People in Kenya Using a Community-Based Distribution Strategy. AIDS Behav [Internet]. 2022 Mar 1 [cited 2024 Aug 25];26(3):964–74. Available from: https://doi.org/10.1007/s10461-021-03451-1

190. Wirtz AL, Naing S, Mon SHH, Paing AZ, Mon EK, Thu KH, et al. High acceptability of HIV self-testing in a randomized trial among transgender women and men who have sex with men, Myanmar. AIDS Care [Internet]. 2022 Apr 3 [cited 2024 Aug 25];34(4):421–9. Available from: https://doi.org/10.1080/09540121.2021.2005763

191. Wixted D, Neighbors CE, Pieper CF, Wu A, Kingsbury C, Register H, et al. Comparison of a Blood Self-Collection System with Routine Phlebotomy for SARS-CoV-2 Antibody Testing. Diagnostics [Internet]. 2022 Aug [cited 2024 Aug 25];12(8):1857. Available from: https://www.mdpi.com/2075-4418/12/8/1857

192. Xu W, Reipold EI, Zhao P, Tang W, Tucker JD, Ong JJ, et al. HCV Self-Testing to Expand Testing: A Pilot Among Men Who Have Sex With Men in China. Front Public Health [Internet]. 2022 May 31 [cited 2024 Aug 25];10. Available from: https://www.frontiersin.org/journals/public-health/articles/10.3389/fpubh.2022.903747/full

193. Yan H, Yang H, Raymond HF, Li J, Shi L en, Huan X, et al. Experiences and correlates of HIV self-testing among men who have sex with men in Jiangsu Province, China. AIDS Behav [Internet]. 2015 Mar [cited 2024 Aug 25];19(3):485–91. Available from: https://www.ncbi.nlm.nih.gov/pmc/articles/PMC4359059/

194. Zhang Y, Guy RJ, Smith KS, Jamil MS, Prestage G, Applegate TL, et al. Sustaining success: a qualitative study of gay and bisexual men's experiences and perceptions of HIV self-testing in a randomized controlled trial. BMC Public Health [Internet]. 2021 Nov 9 [cited 2024 Aug 25];21(1):2048. Available from: https://doi.org/10.1186/s12889-021-12011-0

195. Arias M, Jang D, Gilchrist J, Luinstra K, Li J, Smieja M, et al. Ease, Comfort, and Performance of the HerSwab Vaginal Self-Sampling Device for the Detection of Chlamydia trachomatis and Neisseria gonorrhoeae. Sex Transm Dis. 2016 Feb;43(2):125–9.

196. Ogale YP, Grabowski MK, Nabakka P, Ddaaki W, Nakubulwa R, Nakyanjo N, et al. Self-collected samples as an additional option for STI testing in low-resource settings: a qualitative study of acceptability among adults in Rakai, Uganda. BMJ Open [Internet]. 2023 Nov 1 [cited 2024 Aug 21];13(11):e073241. Available from: https://bmjopen.bmj.com/content/13/11/e073241

197. Ivanova Reipold E, Fajardo E, Juma E, Bukusi D, Bermudez Aza E, Jamil MS, et al. Usability and acceptability of oral fluid hepatitis C self-testing among people who inject drugs in Coastal

Kenya: a cross-sectional pilot study. BMC Infect Dis [Internet]. 2022 Sep 15 [cited 2024 Aug 14];22(1):738. Available from: https://doi.org/10.1186/s12879-022-07712-9

198. Tonen-Wolyec S, Batina-Agasa S, Muwonga J, Bouassa RSM, Tshilumba CK, Bélec L. Acceptability, feasibility, and individual preferences of blood-based HIV self-testing in a population-based sample of adolescents in Kisangani, Democratic Republic of the Congo. PLOS ONE [Internet]. 2019 Jul 1 [cited 2024 Aug 24];14(7):e0218795. Available from: https://journals.plos.org/plosone/article?id=10.1371/journal.pone.0218795

199. Des Marais AC, Zhao Y, Hobbs MM, Barclay L, Brewer NT, Smith JS. Home Self-Collection by Mail to Test for Human Papillomavirus and Sexually Transmitted Infections. Obstet Gynecol [Internet]. 2018 Dec [cited 2024 Aug 13];132(6):1412–20. Available from: https://www.ncbi.nlm.nih.gov/pmc/articles/PMC6249061/

200. Schuster JE, Potts J, Selvarangan R, Mast DK, Goldman JL, for the School TLC Study Group. A COVID-19 Testing Preference Study in Schools. Pediatrics [Internet]. 2023 Jul 1 [cited 2024 Aug 23];152(Supplement 1):e2022060352H. Available from: https://doi.org/10.1542/peds.2022-060352H
